# Supplementary material for: Hypoxia promotes osteogenesis by facilitating acetyl‐CoA‐mediated mitochondrial–nuclear communication
Source: EMBO J. 2022 Oct 24;41(23):e111239. doi: 10.15252/embj.2022111239 (PMC9713713; doi:10.15252/embj.2022111239)
Supplement: Supplementary file 1 — Expanded View Figures PDF [file EMBJ-41-e111239-s008.pdf]

Expanded View Figures

Figure EV1. Quality control measurements of sequencing libraries.

A, B Insert the size distribution of each ATAC-seq library (two libraries per oxygen condition—A) and mapping statistics for each individual replicate (B).
C, D Correlation matrices of H3K27ac ChIP-seq libraries (C) and H3K4me3 ChIP-seq libraries (D).
E Heatmap of H3K4me3 abundance between hypoxia- and normoxia-cultured MSCs.

Data information: $n = 2$ biologically independent experiments.

▶

 © 2022 The Authors

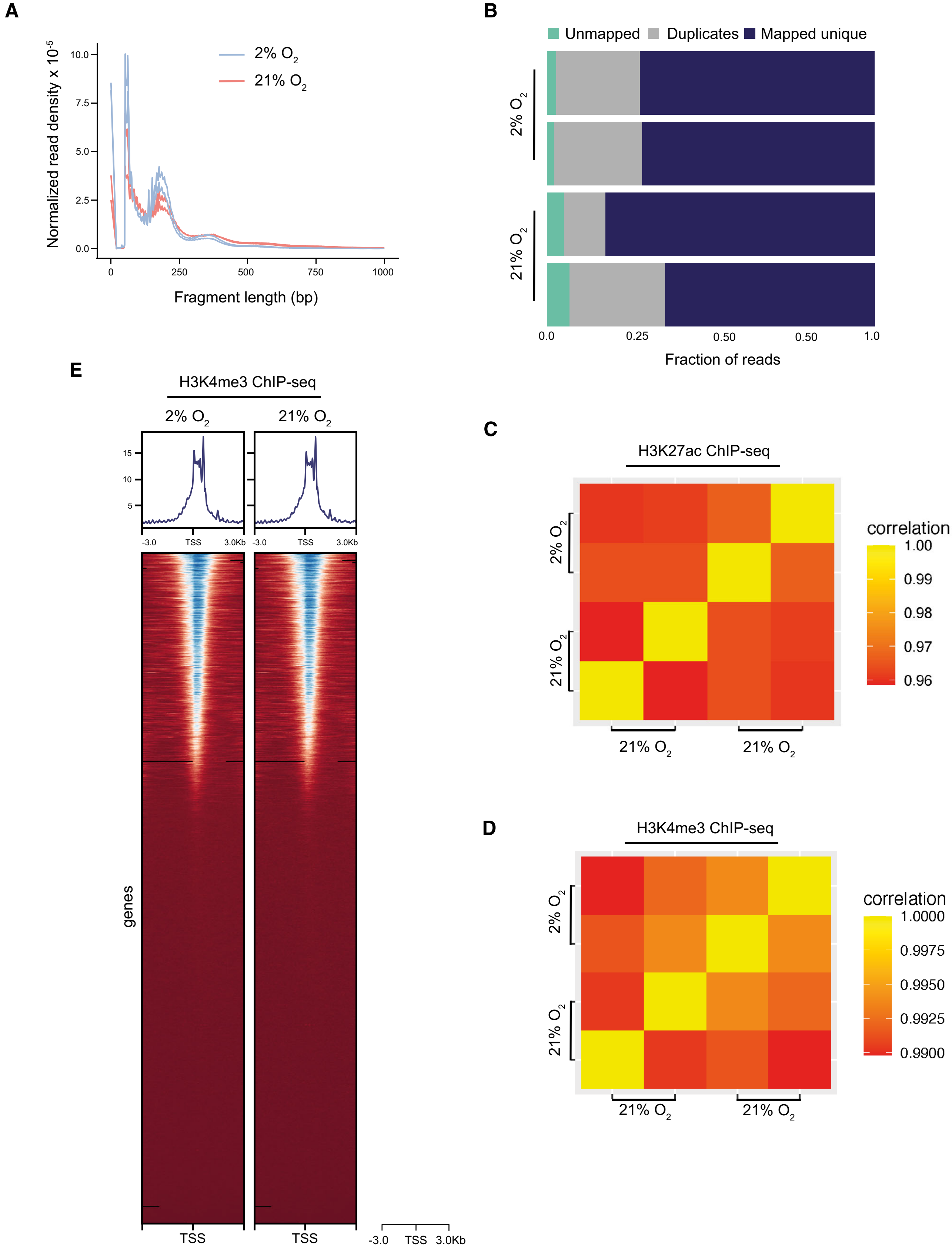
A
Normalized read density x 10^-5
10.0
7.5
5.0
2.5
0.0
0
250
500
750
1000
Fragment length (bp)
2% O2
21% O2
B
Unmapped
Duplicates
Mapped unique
2% O2
21% O2
0.0
0.25
0.50
0.50
1.0
Fraction of reads
C
H3K27ac ChIP-seq
2% O2
21% O2
21% O2
21% O2
correlation
1.00
0.99
0.98
0.97
0.96
D
H3K4me3 ChIP-seq
2% O2
21% O2
21% O2
21% O2
correlation
1.0000
0.9975
0.9950
0.9925
0.9900
E
H3K4me3 ChIP-seq
2% O2
21% O2
15
10
5
-3.0
TSS
3.0Kb
genes
TSS
TSS
-3.0
TSS
3.0Kb

Figure EV1.

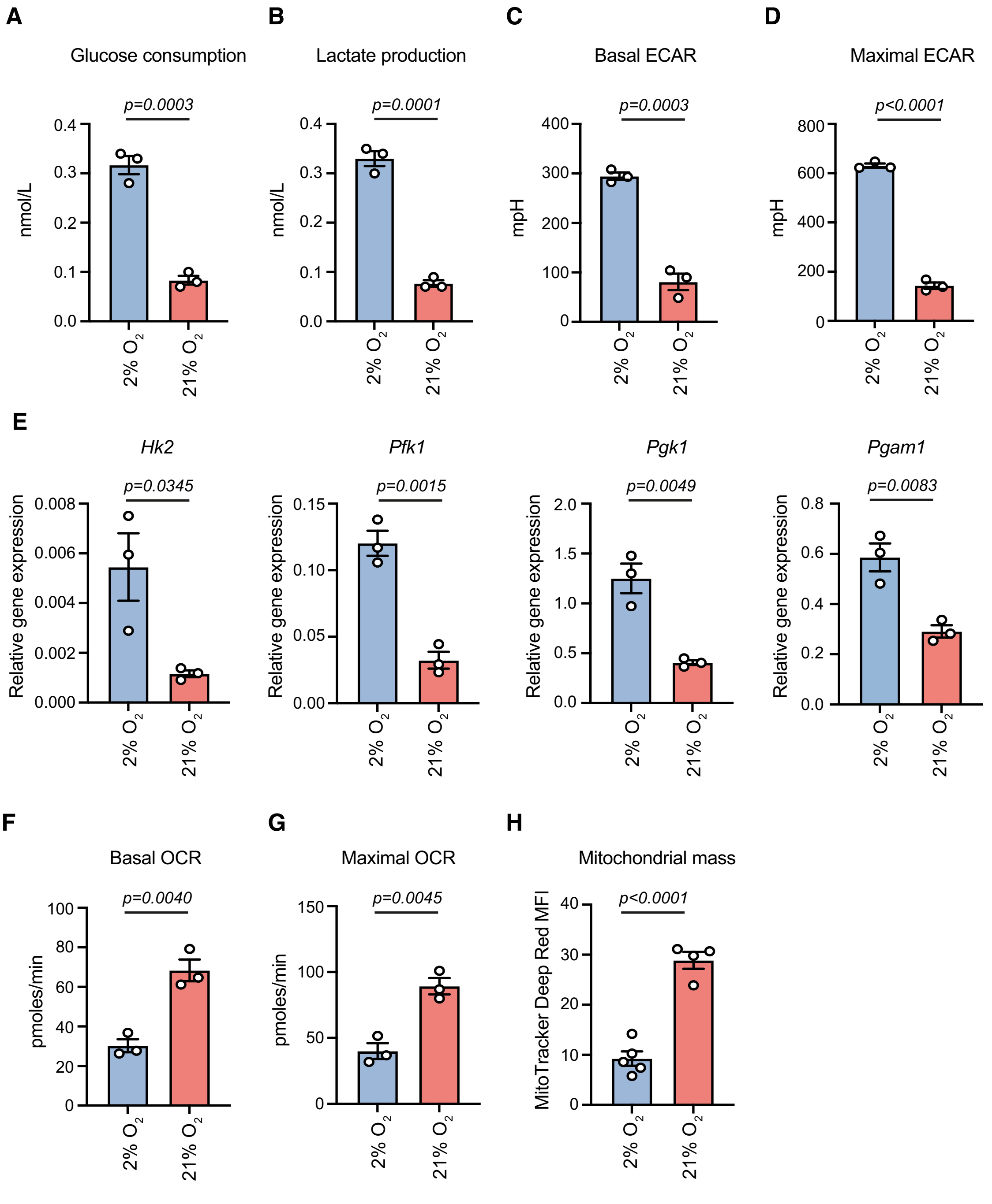

Figure EV2. Metabolic profiling of hypoxic and normoxic MSCs.

A, B Glucose consumption (A) and lactate production (B) were measured in the media of hypoxia- and normoxia-cultured cells using the Vi-Cell MetaFLEX instrument. *n* = 3 biologically independent experiments.

C, D Basal (C) and maximal (D) ECAR in hypoxia- and normoxia-cultured MSCs. *n* = 3 biologically independent experiments.

E qRT-PCR analysis of glycolytic genes in hypoxic and normoxic cells. *β-actin* was used as an internal control for normalization. *n* = 3 biologically independent experiments.

F, G Basal (F) and maximal (G) OCR in hypoxia- and normoxia-cultured MSCs. *n* = 3 biologically independent experiments.

H MFI of hypoxia- and normoxia-cultured cells after staining with the MitoTracker Deep Red FM dye. *n* = 4 biologically independent experiments.

Data information: Results are shown as mean ± SEM and statistical significance was determined using a two-sided unpaired *t*-test.
Source data are available online for this figure.

© 2022 The Authors

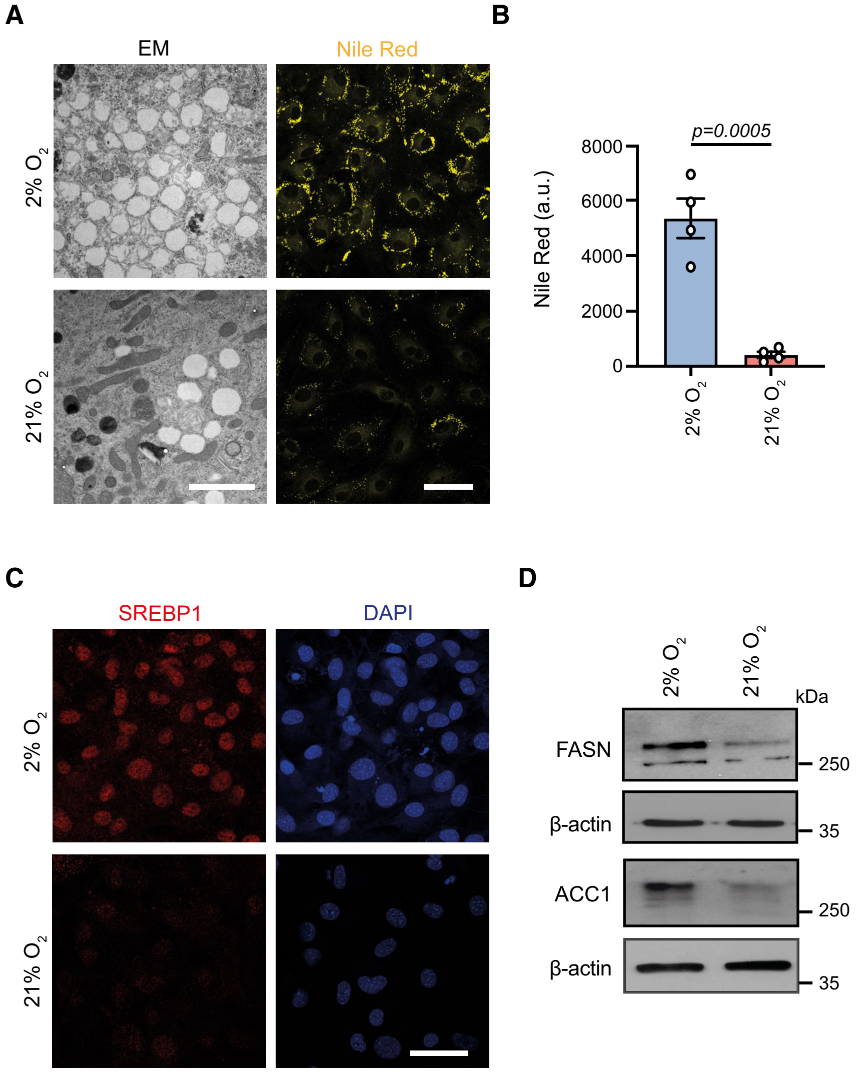

Figure EV3. Impaired lipogenesis of normoxia-cultured MSCs is not due to lower CiC levels.

A, B Representative images (A) and quantification of lipid droplets after observing cells under the electron microscope (left) and after staining lipids with Nile Red (right-B). Scale bars, 2 μm for electron microscopy images and 50 μm for confocal images. $n = 4$ biologically independent experiments and merged results are shown in (B). Results are shown as mean ± SEM and statistical significance was determined using a two-sided unpaired t-test.

C Representative images of hypoxia- and normoxia-cultured cells after immunostaining against SREBP1. Scale bar, 50 μm.

D Representative immunoblots against FASN and ACC1 in hypoxia- and normoxia-cultured cells. β-actin was used as a loading control. $n = 3$ biologically independent experiments.

Source data are available online for this figure.

© 2022 The Authors

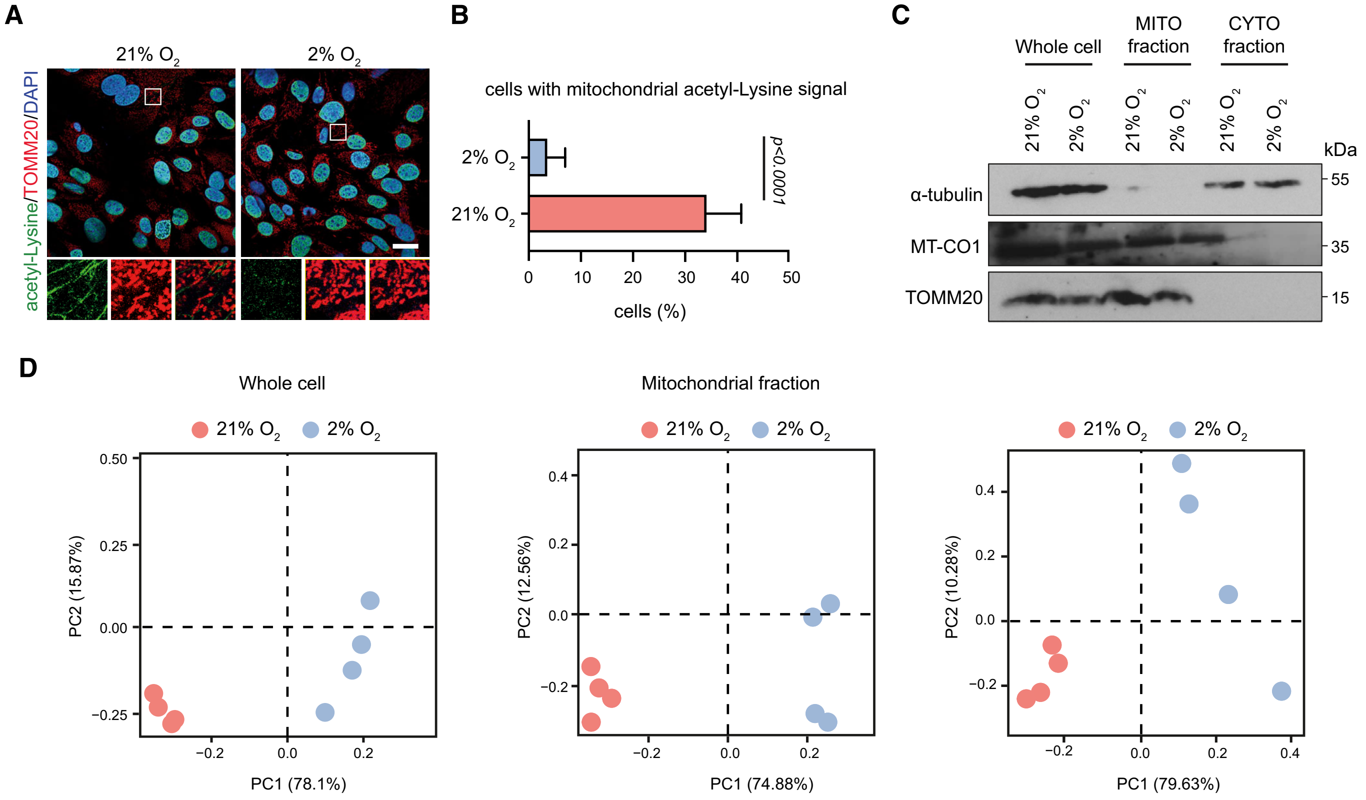

Figure EV4. Quality control measurements of commercial MSCs fractionation.

A, B Representative images after immunostaining of normoxic and hypoxic commercially available MSCs against acetyl-lysine and TOMM20 (A) and assessment of cells (%) with mitochondrial acetyl-lysine signal localization (B). Nuclei were stained with DAPI. Quantification of cells (%) with mitochondrial acetyl-lysine signal in (B) from $n = 201$ hypoxic and $n = 205$ normoxic individual cells from a representative experiment of two biologically independent experiments is shown in (B). Results are shown as mean ± SEM and statistical significance was determined using a two-sided unpaired t-test. In magnified insets, the intensity of the acetyl-lysine signal was adjusted similarly to all samples for visualization purposes. Scale bar, 25 μm.

C Representative immunoblots against the mitochondrial proteins TOMM20 and MT-CO1 and the cytosolic protein α-tubulin in whole lysates, mitochondrial fractions and cytosolic fractions of normoxia- and hypoxia-cultured MSCs. $n = 4$ biologically independent experiments.

D Principal component analysis (PCA) plot showing clustering of normoxia- and hypoxia-cultured cells after metabolite extraction from the whole cell, mitochondrial and cytosolic fractions. $n = 4$ biologically independent experiments.

Source data are available online for this figure.

 © 2022 The Authors

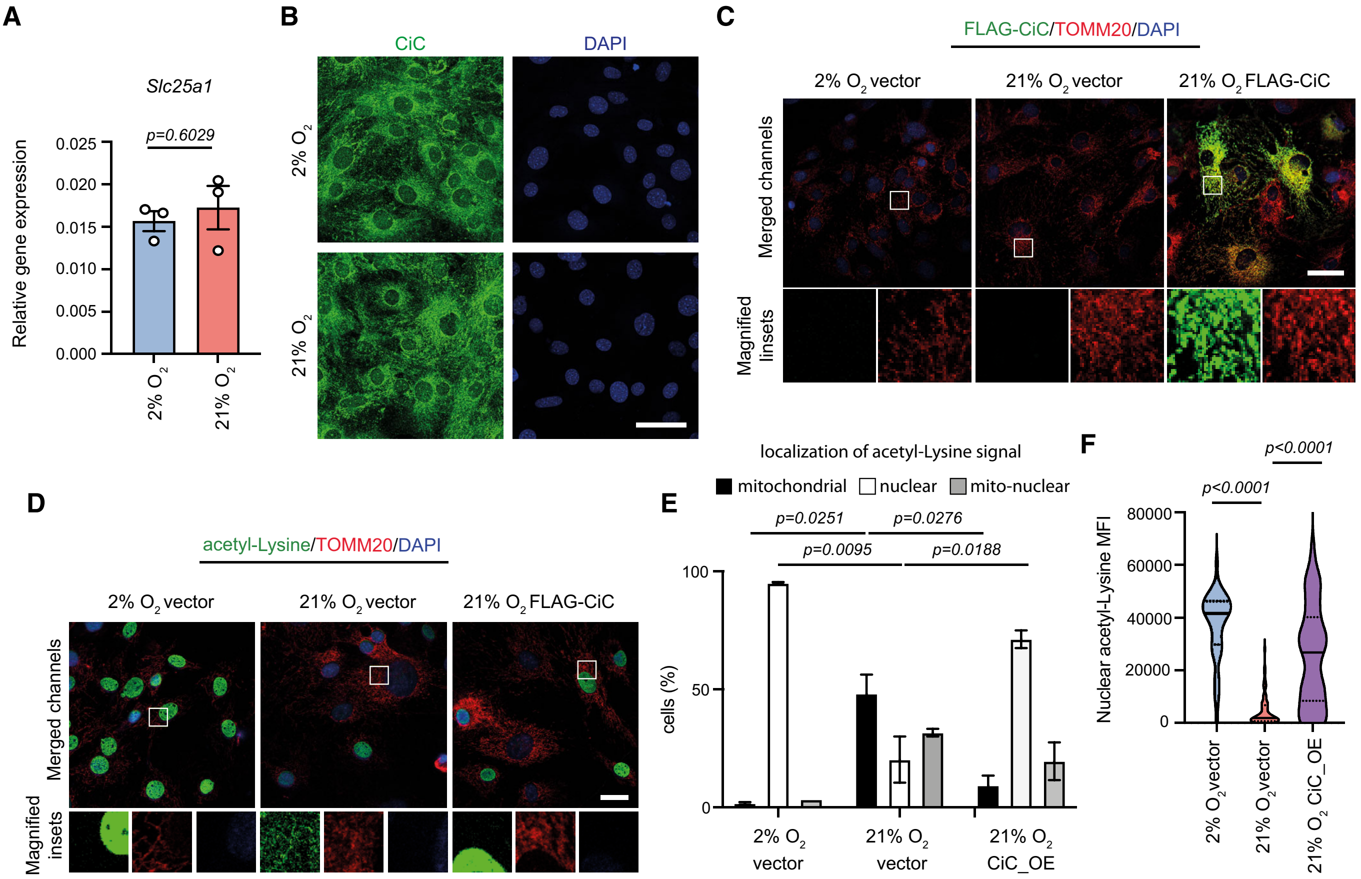

Figure EV5. Impaired CiC function in normoxic MSCs.

A qRT-PCR analysis of *Slc25a1*, which encodes citrate carrier. *β-actin* was used as an internal control for normalization. $n = 3$ biologically independent experiments. Results are shown as mean ± SEM and statistical significance was determined using a two-sided unpaired *t*-test.

B Representative images of hypoxia- and normoxia-cultured cells after immunostaining against CiC. Scale bar, 50 µm.

C Representative images after staining hypoxic and normoxic cells using an anti-FLAG antibody. Cells were transfected with either a vector plasmid or a FLAG-CiC-expressing plasmid. TOMM20 was used as a counterstain for mitochondria to confirm proper localization of the exogenously expressed CiC-FLAG protein, as shown in the magnified inset. Transfection and all downstream experiments were done for $n = 2$ biologically independent experiments. Scale bar; 50 µm.

D–F Representative images after staining cells used in (C) against acetyl-lysine and TOMM20 (D), assessment of the localization, as described above (E), and quantification of nuclear acetyl-lysine signal MFI (F). Nuclei were stained with DAPI. Quantification of nuclear acetyl-lysine MFI from $n = 43$ hypoxic, $n = 63$ normoxic and $n = 38$ normoxic_CiC OE individual cells from a representative experiment of two biologically independent experiments is shown in (F). Results are shown as mean ± SEM and statistical significance was determined with ordinary one-way ANOVA, using the Holm–Sidak's multiple-comparisons test in Panels (E) and (F). The distribution of data points in (F) is shown as a violin plot, where the mean is indicated by a solid line and the quartiles are indicated with dashed lines. In magnified insets, the intensity of the acetyl-lysine signal was adjusted similarly to all samples, for visualization purposes. Scale bar, 25 µm.

Source data are available online for this figure.

© 2022 The Authors
