# Supplementary material for: Hypoxia promotes osteogenesis by facilitating acetyl‐CoA‐mediated mitochondrial–nuclear communication
Source: EMBO J. 2022 Oct 24;41(23):e111239. doi: 10.15252/embj.2022111239 (PMC9713713; doi:10.15252/embj.2022111239)
Supplement: Supplementary file 3 — Source Data for Expanded View [file EMBJ-41-e111239-s003.zip › Figure EV3.pdf]

Panel EV3B: Nile red MFI

2% O2	$21\%\ O_2$
5930	310
4919	680.2
3592	164.7
6932	511.25

Table Analyzed	all replicates	
Column B	$21\%\ O_2$	
vs.	vs,	
Column A	2% O2	
Unpaired t test		
P value		0.0005
P value summary	***	
Significantly different (P < 0.05)?	Yes	
One- or two-tailed P value?	Two-tailed	
t, df	t=6,817, df=6	
How big is the difference?		
Mean of column A		5343
Mean of column B		416.5
Difference between means (B - A) ± SEM	-4927 ± 722,8	
95% confidence interval	-6695 to -3158	
R squared (eta squared)		0.8856
F test to compare variances		
F, DFn, Dfd	39,90, 3, 3	
P value		0.0129
P value summary	*	
Significantly different (P < 0.05)?	Yes	
Data analyzed		
Sample size, column A		4
Sample size, column B		4
