# Supplementary material for: Hypoxia promotes osteogenesis by facilitating acetyl‐CoA‐mediated mitochondrial–nuclear communication
Source: EMBO J. 2022 Oct 24;41(23):e111239. doi: 10.15252/embj.2022111239 (PMC9713713; doi:10.15252/embj.2022111239)
Supplement: Supplementary file 3 — Source Data for Expanded View [file EMBJ-41-e111239-s003.zip › Figure EV2.pdf]

Panel EV2A: Glucose consumption

2% O2	21% O2
0.33	0.07
0.28	0.08
0.34	0.1
Table Analyzed	Glucose consumption
Column B	21% o2
vs.	vs,
Column A	2% o2
Unpaired t test	
P value	0.0003
P value summary	***
Significantly different (P < 0.05)?	Yes
One- or two-tailed P value?	Two-tailed
t, df	t=11,36, df=4
How big is the difference?	
Mean of column A	0.3167
Mean of column B	0.08333
Difference between means (B - A) ± SEM	-0,2333 ± 0,02055
95% confidence interval	-0,2904 to -0,1763
R squared (eta squared)	0.9699
F test to compare variances	
F, DFn, Dfd	4,429, 2, 2
P value	0.3684
P value summary	ns
Significantly different (P < 0.05)?	No
Data analyzed	
Sample size, column A	3
Sample size, column B	3

Panel EV2B: Lactate production

2% O2	21% O2
0.35	0.07
0.3	0.07
0.34	0.09
Table Analyzed	lactate production
Column B	21% o2
vs.	vs,
Column A	2% o2
Unpaired t test	
P value	0.0001
P value summary	***
Significantly different (P < 0.05)?	Yes
One- or two-tailed P value?	Two-tailed
t, df	t=15,20, df=4
How big is the difference?	
Mean of column A	0.33
Mean of column B	0.07667
Difference between means (B - A) ± SEM	-0,2533 ± 0,01667
95% confidence interval	-0,2996 to -0,2071
R squared (eta squared)	0.983
F test to compare variances	
F, DFn, Dfd	5,250, 2, 2
P value	0.32
P value summary	ns
Significantly different (P < 0.05)?	No
Data analyzed	
Sample size, column A	3
Sample size, column B	3

Panel EV2C: Basal ECAR

2% O2	21% O2
282.4	104.1
308.3	48.41
293	89.74
Table Analyzed	Glycolysis
Column A	**2% O_2**
vs.	vs,
Column B	**21% O_2**
Unpaired t test	
P value	0.0003
P value summary	***
Significantly different (P < 0.05)?	Yes
One- or two-tailed P value?	Two-tailed
t, df	t=11,68, df=4
How big is the difference?	
Mean of column A	294.6
Mean of column B	80.75
Difference between means (A - B) ± SEM	213,8 ± 18,31
95% confidence interval	163,0 to 264,6
R squared (eta squared)	0.9715
F test to compare variances	
F, DFn, Dfd	4,931, 2, 2
P value	0.3372
P value summary	ns
Significantly different (P < 0.05)?	No
Data analyzed	
Sample size, column A	3
Sample size, column B	3

Panel EV2D: Maximal ECAR

2% O_2	21% O_2
622.6	123.7
623.6	138.5
647.5	168.1
Table Analyzed	Glycolytic Capacity
Column A	**2% O_2**
vs.	vs,
Column B	**21% O_2**
Unpaired t test	
P value	<0,0001
P value summary	****
Significantly different (P < 0.05)?	Yes
One- or two-tailed P value?	Two-tailed
t, df	t=31,71, df=4
How big is the difference?	
Mean of column A	631.2
Mean of column B	143.4
Difference between means (A - B) ± SEM	487,8 ± 15,38
95% confidence interval	445,1 to 530,5
R squared (eta squared)	0.996
F test to compare variances	
F, DFn, Dfd	2,572, 2, 2
P value	0.5599
P value summary	ns
Significantly different (P < 0.05)?	No
Data analyzed	
Sample size, column A	3
Sample size, column B	3

Panel EV2E: RT-qPCR analysis of glycolytic genes

Hk2

2% O_2	21% O_2	
0.00751		0.001384263
0.00289		0.001180271
0.00595		0.000909062
Table Ana	hk2	
Column B	21% O_2	
vs.	vs,	
Column A	2% O_2	
Unpaired t test		
P value		0.0345
P value su	*	
Significan	Yes	
One- or tv	Two-tailed	
t, df	t=3,149, df=4	
How big is the difference?		
Mean of c		0.005451
Mean of c		0.001158
Difference	-0,004293 ± 0,001363	
95% confi	-0,008078 to -0,0005079	
R squarec		0.7126
F test to compare variances		
F, DFn, D	97,11, 2, 2	
P value		0.0204
P value su	*	
Significan	Yes	
Data analyzed		
Sample si		3
Sample si		3

Pfk1

2% O_2	21% O_2	
0.1169		0.02936009
0.10584		0.02330312
0.13806		0.04439887
Table Ana	pfk1	
Column B	21% O_2	
vs.	vs,	
Column A	2% O_2	
Unpaired t test		
P value		0.0015
P value su	**	
Significan	Yes	
One- or tv	Two-tailed	
t, df	t=7,751, df=4	
How big is the difference?		
Mean of c		0.1203
Mean of c		0.03235
Difference	-0,08791 ± 0,01134	
95% confi	-0,1194 to -0,05642	
R squarec		0.9376
F test to compare variances		
F, DFn, D	2,271, 2, 2	
P value		0.6114
P value su	ns	
Significan	No	
Data analyzed		
Sample si		3
Sample si		3

Pgk1

2% O_2	21% O_2	
1.47768		0.4042538
0.97265		0.3660214
1.30134		0.4485477
Table Ana	pgk1	
Column B	21% O_2	
vs.	vs,	
Column A	2% O_2	
Unpaired t test		
P value		0.0049
P value su	**	
Significan	Yes	
One- or tv	Two-tailed	
t, df	t=5,633, df=4	
How big is the difference?		
Mean of c		1.251
Mean of c		0.4063
Difference	-0,8443 ± 0,1499	
95% confi	-1,260 to -0,4281	
R squarec		0.888
F test to compare variances		
F, DFn, D	38,52, 2, 2	
P value		0.0506
P value su	ns	
Significan	No	
Data analyzed		
Sample si		3
Sample si		3

Pgam1

2% O_2	21% O_2	
0.67206		0.2825674
0.48185		0.2534899
0.6043		0.3368084
Table Ana	pgam1	
Column B	21% O_2	
vs.	vs,	
Column A	2% O_2	
Unpaired t test		
P value		0.0083
P value su	**	
Significan	Yes	
One- or tv	Two-tailed	
t, df	t=4,856, df=4	
How big is the difference?		
Mean of c		0.5861
Mean of c		0.291
Difference	-0,2951 ± 0,06078	
95% confi	-0,4639 to -0,1264	
R squarec		0.8549
F test to compare variances		
F, DFn, D	5,197, 2, 2	
P value		0.3227
P value su	ns	
Significan	No	
Data analyzed		
Sample si		3
Sample si		3

Panel EV2F: Basal OCR

2% O_2	21% O_2	
36.762		79.234
26.298		61.212
27.714		64.74
Table Ana	BASAL OCR	
Column B	21% O_2	
vs.	vs,	
Column A	2% O_2	
Unpaired t test		
P value		0.004
P value su	**	
Significan	Yes	
One- or tv	Two-tailed	
t, df	t=5,945, df=4	
How big is the difference?		
Mean of c		30.26
Mean of c		68.4
Difference	38,14 ± 6,415	
95% confi	20,33 to 55,95	
R squarec		0.8983
F test to compare variances		
F, DFn, D	2,830, 2, 2	
P value		0.5221
P value su	ns	
Significan	No	
Data analyzed		
Sample si		3
Sample si		3

Panel EV2G: Maximal OCR

2% O_2	21% O_2	
51.594		86.936
31.814		79.984
36.776		100.9425
Table Ana	MAXIMAL OCR	
Column B	21% O_2	
vs.	vs,	
Column A	2% O_2	
Unpaired t test		
P value		0.0045
P value su	**	
Significan	Yes	
One- or tv	Two-tailed	
t, df	t=5,750, df=4	
How big is the difference?		
Mean of c		40.06
Mean of c		89.29
Difference	49,23 ± 8,561	
95% confi	25,46 to 73,00	
R squarec		0.8921
F test to compare variances		
F, DFn, D	1,076, 2, 2	
P value		0.9634
P value su	ns	
Significan	No	
Data analyzed		
Sample si		3
Sample si		3

Panel EV2H: Mitochondrial mass

2% O_2	21% O_2	
10.246		23.895
5.772		29.793
7.413		31.119
14.194		30.674
8.578		
Table Ana	MitoTracker	
Column B	21% O_2	
vs.	vs,	
Column A	2% O_2	
Unpaired t test		
P value	<0,0001	
P value su	****	
Significan	Yes	
One- or tv	Two-tailed	
t, df	t=8,924, df=7	
How big is the difference?		
Mean of c		9.241
Mean of c		28.87
Difference	19,63 ± 2,200	
95% confi	14,43 to 24,83	
R squarec		0.9192
F test to compare variances		
F, DFn, D	1,093, 3, 4	
P value		0.8966
P value su	ns	
Significan	No	
Data analyzed		
Sample si		5
Sample si		4
