# Supplementary material for: Hypoxia promotes osteogenesis by facilitating acetyl‐CoA‐mediated mitochondrial–nuclear communication
Source: EMBO J. 2022 Oct 24;41(23):e111239. doi: 10.15252/embj.2022111239 (PMC9713713; doi:10.15252/embj.2022111239)
Supplement: Supplementary file 3 — Source Data for Expanded View [file EMBJ-41-e111239-s003.zip › Figure EV5.pdf]

Panel EV5A: qRT-PCR of Slc25a1

2% O_2	21% O_2
0.01705888	0.01910375
0.01332242	0.01220261
0.01663078	0.0204749

Table Analyzed	CiC white	
Column B	21% O_2	
vs.	vs,	
Column A	2% O_2	
Unpaired t test		
P value		0.6029
P value summary	ns	
Significantly different (P < 0.05)?	No	
One- or two-tailed P value?	Two-tailed	
t, df	t=0,5640, df=4	
How big is the difference?		
Mean of column A		0.01567
Mean of column B		0.01726
Difference between means (B - A) ± SEM	0,001590 ± 0,002819	
95% confidence interval	-0,006237 to 0,009416	
R squared (eta squared)		0.07366
F test to compare variances		
F, DFn, Dfd	4,701, 2, 2	
P value		0.3508
P value summary	ns	
Significantly different (P < 0.05)?	No	
Data analyzed		
Sample size, column A		3
Sample size, column B		3

Panel EV5E:scoring of acetyl_lysine signal (% cells)

nuclear

2% O2	21% O2	21% O2 CiC OE
94.5652	10.4166667	67.5
95.5556	30	75

Number o	1
Number o	3
Alpha	0.05

Holm-Šídá	Mean Diff,	Below threshold?	Summary	Adjusted P Value	
2% o2 vs.	74.85	Yes	**	0.0095	A-B
2% o2 vs.	23.81	No	ns	0.0691	A-C
21% o2 vs	-51.04	Yes	*	0.0188	B-C

Test detai	Mean 1	Mean 2	Mean Diff,	SE of diff,	n1	n2	t	DF
2% o2 vs.	95.06	20.21	74.85	8.571	2	2	8.734	3
2% o2 vs.	95.06	71.25	23.81	8.571	2	2	2.778	3
21% o2 vs	20.21	71.25	-51.04	8.571	2	2	5.955	3

mitochondrial

2% O2	21% O2	!1% O2 cic
2.17391	56.25	5
1.11111	40	13.4615

Number o	1
Number o	3
Alpha	0.05

Holm-Šídá	Mean Diff,	Below thre	Summary	Adjusted P Value	
2% o2 vs.	-46.48	Yes	*	0.0251	A-B
2% o2 vs.	-7.588	No	ns	0.3857	A-C
21% o2 vs	38.89	Yes	*	0.0276	B-C

Test detai	Mean 1	Mean 2	Mean Diff,	SE of diff,	n1	n2	t	DF
2% o2 vs.	1.643	48.13	-46.48	7.492	2	2	6.204	3
2% o2 vs.	1.643	9.231	-7.588	7.492	2	2	1.013	3
21% o2 vs	48.13	9.231	38.89	7.492	2	2	5.191	3

Panel EV5F:Nuclear MFI

2% O2	21% O2	21% O_2 CiC
42391	13891	30958
8905	255	19823
36510	4771	30039
29749	14978	44323
16503	683	12826
38799	6801	44388
9985	20897	33512
31410	28039	39066
26666	6793	15204
28054	1539	1769
47353	2725	34778
25907	671	53796
25461	831	2656
1479	19122	2593
13896	6734	2898
42852	7300	55799
18490	3385	50992
39453	552	1789
41660	8073	13180
46999	10168	29178
45546	547	13468
30526	8056	493
49564	2464	32759
41620	1758	8561
45085	1033	54509
42443	2000	52599
44150	1263	24339
33413	13811	32030
39765	1557	28950
49323	557	34979
47647	705	24583
52909	1706	29
43298	1391	1397
43171	2897	8179
46898	369	65037
49617	399	43494
60946	166	10115
46233	1178	14851
43695	1607	
43391	60	
38333	1598	
35191	5216	
48666	1726	
	3428	
	682	
	601	
	3185	
	291	
	2188	
	581	
	5419	
	871	
	1760	
	408	
	2288	
	1053	
	10580	
	7413	
	1875	
	5121	
	2919	
	9925	
	16338	

Number of families	1
Number of comparisons per family	3
Alpha	0.05

Holm-Šídák's multiple comparisons test	Mean Diff,	Below threshold?	Summary	Adjusted P Value	
2% O2 vs. 21% O2	32510	Yes	****	<0,0001	A-B
2% O2 vs. 21% O_2 CiC	11544	Yes	****	<0,0001	A-C
21% O2 vs. 21% O_2 CiC	-20966	Yes	****	<0,0001	B-C

Test details	Mean 1	Mean 2	Mean Diff,	SE of diff,	n1	n2	t	DF
2% O2 vs. 21% O2	37069	4559	32510	2492	43	63	13.04	141
2% O2 vs. 21% O_2 CiC	37069	25525	11544	2805	43	38	4.115	141
21% O2 vs. 21% O_2 CiC	4559	25525	-20966	2588	63	38	8.102	141
