# Supplementary material for: Hypoxia promotes osteogenesis by facilitating acetyl‐CoA‐mediated mitochondrial–nuclear communication
Source: EMBO J. 2022 Oct 24;41(23):e111239. doi: 10.15252/embj.2022111239 (PMC9713713; doi:10.15252/embj.2022111239)
Supplement: Supplementary file 3 — Source Data for Expanded View [file EMBJ-41-e111239-s003.zip › Figure EV4.pdf]

Panel EV4B: commercial MSCs with mitochondrial acetyl-Lysine signal (%)

2% O2	21% O2
46.6	0
34.7	0
26.6	6.6
33.3	3.8
35.4	9.09
28.5	3.5
44.4	5.5
28.5	0
31.5	
31.8	

Table Analyzed	Data 1
Column B	2% O_2
vs.	vs,
Column A	21% O_2
Unpaired t test	
P value	<0,0001
P value summary	****
Significantly different (P < 0.05)?	Yes
One- or two-tailed P value?	Two-tailed
t, df	t=11,79, df=16
How big is the difference?	
Mean of column A	34.13
Mean of column B	3.561
Difference between means (B - A) ± SEM	-30,57 ± 2,592
95% confidence interval	-36,06 to -25,07
R squared (eta squared)	0.8968
F test to compare variances	
F, DFn, Dfd	3,772, 9, 7
P value	0.0939
P value summary	ns
Significantly different (P < 0.05)?	No
Data analyzed	
Sample size, column A	10
Sample size, column B	8
