# Supplementary material for: Hypoxia promotes osteogenesis by facilitating acetyl‐CoA‐mediated mitochondrial–nuclear communication
Source: EMBO J. 2022 Oct 24;41(23):e111239. doi: 10.15252/embj.2022111239 (PMC9713713; doi:10.15252/embj.2022111239)
Supplement: Supplementary file 4 — Source Data for Figure 1 [file EMBJ-41-e111239-s006.pdf]

Pnael 1C: Oil Red O quantification

2% O_2	21% O_2
0.05735	0.05705
0.05665	0.06505
0.06255	0.07845
0.07555	0.06335
0.06565	0.05665

Column B	**21% O_2**	
vs.	vs,	
Column A	**2% O_2**	
Unpaired t test		
P value		0.9174
P value summary	ns	
Significantly different (P < 0.05)?	No	
One- or two-tailed P value?	Two-tailed	
t, df	t=0,1070, df=8	
How big is the difference?		
Mean of column A		0.06355
Mean of column B		0.06411
Difference between means (B - A) ± SEM	0,0005600 ± 0,005235	
95% confidence interval	-0,01151 to 0,01263	
R squared (eta squared)		0.001428
F test to compare variances		
F, DFn, Dfd	1,328, 4, 4	
P value		0.7903
P value summary	ns	
Significantly different (P < 0.05)?	No	
Data analyzed		
Sample size, column A		5
Sample size, column B		5

Panel 1E: Alizarin Red S qauntification

2% O_2		21% O_2	R-2% O_2			
	86755	3438	4159			
	96475	2183	1788			
	62851	6310	2527			
		1426	1999			
		2396	4708			
Number of families		1				
Number of comparisons per family		3				
Alpha		0.05				
Holm-Šídák's multiple comparisons test	Mean Diff,		Below thre	Summary		Adjusted P Value
2% O2 vs. 21% O_2		78876	Yes	****		<0,0001
2% O2 vs. R-2% O_2		78991	Yes	****		<0,0001
21% O_2 vs. R-2% O_2		114.4	No	ns		0.9821
Test details	Mean 1		Mean 2	Mean Diff,		SE of diff,
2% O2 vs. 21% O_2		82027	3151		78876	5752
2% O2 vs. R-2% O_2		82027	3036		78991	5752
21% O_2 vs. R-2% O_2		3151	3036		114.4	4981

Figure 1G: GO enrichment analysis RNA-seq

Cytoplasmic ribosomal proteins	19.51
Vasculature development	9.18
Canonical Wnt signaling	8
Ossification	7.61
Elastic fibre formation	7.01
Mesenchymal cell differentiation	6.33
ECM-receptor interaction	6.18
Org. hydr. compound metabolic process	6.13
Reg. of epithelial cel proliferation	5.58
Non-canonical Wnt signaling	5.57
