# Supplementary material for: Hypoxia promotes osteogenesis by facilitating acetyl‐CoA‐mediated mitochondrial–nuclear communication
Source: EMBO J. 2022 Oct 24;41(23):e111239. doi: 10.15252/embj.2022111239 (PMC9713713; doi:10.15252/embj.2022111239)
Supplement: Supplementary file 5 — Source Data for Figure 3 [file EMBJ-41-e111239-s009.pdf]

Pnael 3b: Histone SILAC-MS 2% O2 vs 21% O2

H2AK5ac	0.91130869
H2AK9ac	0.04455503
H2BK5ac	0.64571903
H2BK12ac	0.54985095
H2BK15ac	0.7281806
H2BK20ac	0.21062584
H3K9ac	0.79703967
H3K9me2	-0.4505237
H3K9me3	0.01838693
H3K14ac	0.62041159
H3K18ac	0.59407685
H3K23ac	0.16899285
H3K27me2	0.09505032
H3K36me2	-0.0058238
H3K79me	-0.1561435
H4K5ac	0.27984169
H4K8ac	0.25789954
H4K12ac	-0.0196673
H4K16ac	0.41557926
H4K20me2	0.26918458
H4K91me2	-0.0333945

Pnael 3d: Histone H3 acetylation

2% O_2	21% O_2
4.42054	3.95940452
4.17101	1.96456287
3.43212	3.47506399
4.30296	2.92628623
3.06123	3.80443937
6.11727	4.10739939
5.45845	3.41221109
4.87597	4.08569933
3.72555	4.99542696
3.73152	2.29679262
5.22216	2.97058414
2.77896	4.47707035
4.20997	4.18478192
2.76351	4.73287309
2.90293	3.19652696
2.46136	4.53217269
5.17148	5.14965247
2.99741	4.83118029
3.75272	3.28552267
4.34567	3.30964018
4.20433	4.63717602
5.38329	4.064712
3.50261	3.10322896
4.17682	4.17929268
3.20477	3.69021521
2.1244	3.94087714
3.62471	3.79841918
4.38952	3.71993293
4.09951	2.61793638
3.49689	2.5229247
3.92883	0.95043278
3.22512	3.105694
5.0384	1.85241937
4.33011	3.82072214
4.84357	4.30685488
4.37248	2.89618725
5.9454	1.84129924
3.53103	3.32390194
3.904	3.24031537
2.9607	2.9024518
5.53221	2.84222376
5.4376	2.50290631
3.85997	3.87049336
3.78452	3.71498937
2.1242	1.40494997
5.04937	3.25184711
3.82954	2.92822838
3.59638	3.20735019
3.83934	3.4958266
6.73673	2.76154392
6.03851	3.00280233
5.29051	3.19226928
4.88569	2.96842439
2.89904	3.79405822
4.79868	3.25631107
5.14959	2.78022966
5.36237	
3.83647	
5.15907	
3.70112	
6.07756	
5.0889	
6.45624	
4.61968	
5.04754	
5.68656	
4.74114	

Table Ana	H3ac/H3	
Column B	21% O_2	
vs.	vs,	
Column A	2% O_2	
Unpaired t test		
P value	<0,0001	
P value su	****	
Significan	Yes	
One- or tw	Two-tailed	
t, df	t=5,257, df=121	
How big is the difference?		
Mean of c		4.311
Mean of c		3.378
Difference	-0,9324 ± 0,1773	
95% confi	-1,283 to -0,5813	
R squared		0.186
F test to compare variances		
F, DFn, D	1,478, 66, 55	
P value		0.1375
P value su	ns	
Significan	No	
Data analyzed		
Sample si		67
Sample si		56

Panel 3f: decreased K27ac in 21% O2

nent-based	25.4462
ystem dev	17.1865
on of cell a	13.8398
Wnt signa	12.5041
of cell dev	11.8663

Panel 3F: increased K27ac in 21% O2

phosphor	16.8932
nent-based	15.4716
n of MAPK	11.6451
ellular resp	11.087
GTPase	9.90711

Panel 3I: Acetyl-CoA

2% O_2	21% O_2
0.00072	0.000942608
0.00062	0.001094103
0.00067	0.000782986

Table Ana	Acetyl-CoA	
Column A	**2% O_2**	
vs.	vs,	
Column B	**21% O_2**	
Unpaired t test		
P value		0.047
P value su	*	
Significan	Yes	
One- or tw	Two-tailed	
t, df	t=2,836, df=4	
How big is the difference?		
Mean of c		0.0006731
Mean of c		0.0009399
Difference	-0,0002668 ± 9,406e-005	
95% confi	-0,0005279 to -5,633e-006	
R squared		0.6679
F test to compare variances		
F, DFn, D	10,35, 2, 2	
P value		0.1762
P value su	ns	
Significan	No	
Data analyzed		
Sample si		3
Sample si		3
