# Supplementary material for: Hypoxia promotes osteogenesis by facilitating acetyl‐CoA‐mediated mitochondrial–nuclear communication
Source: EMBO J. 2022 Oct 24;41(23):e111239. doi: 10.15252/embj.2022111239 (PMC9713713; doi:10.15252/embj.2022111239)
Supplement: Supplementary file 6 — Source Data for Figure 4 [file EMBJ-41-e111239-s004.pdf]

Panel 4B:scoring of acetyl_lysine signal (% cells)

mitochondrial		nuclear	
**2% O2**	**21% O2**	**2% O2**	**21% O2**
0	26.41	77.9	13.2
2.7	16.32	79.72	40.81
6.66	45.58	73.33	11.76

Table Analyzed	mitochondrial		Table Analyzed	nuclear	
Column B	21% o2		Column B	21% o2	
vs.	vs,		vs.	vs,	
Column A	2% o2		Column A	2% o2	
Unpaired t test			Unpaired t test		
P value		0.0403	P value		0.0047
P value summary	*		P value summary	**	
Significantly different (P < 0.05)?	Yes		Significantly different (P < 0.05)?	Yes	
One- or two-tailed P value?	Two-tailed		One- or two-tailed P value?	Two-tailed	
t, df	t=2,992, df=4		t, df	t=5,711, df=4	
How big is the difference?			How big is the difference?		
Mean of column A		3.12	Mean of column A		76.98
Mean of column B		29.44	Mean of column B		21.92
Difference between means (B - A) ± SEM	26,32 ± 8,796		Difference between means (B - A) ± SEM	-55,06 ± 9,642	
95% confidence interval	1,894 to 50,74		95% confidence interval	-81,83 to -28,29	
R squared (eta squared)		0.6911	R squared (eta squared)		0.8907
F test to compare variances			F test to compare variances		
F, DFn, Dfd	19,69, 2, 2		F, DFn, Dfd	24,73, 2, 2	
P value		0.0967	P value		0.0777
P value summary	ns		P value summary	ns	
Significantly different (P < 0.05)?	No		Significantly different (P < 0.05)?	No	
Data analyzed			Data analyzed		
Sample size, column A		3	Sample size, column A		3
Sample size, column B		3	Sample size, column B		3

Panel 4D: levels of acetyl-CoA and citrate in whole cell, mitochondrial and nuclear fractions

Whole cell_acetyl-CoA		Whole cell_citrate		CYTO_acetyl-CoA		CYTO_citrate		MITO_acetyl-CoA		MITO_citrate	
**21% O_2**	**2% O_2**	**21% O_2**	**2% O_2**	**21% O_2**	**2% O_2**	**21% O_2**	**2% O_2**	**21% O_2**	**2% O_2**	**21% O_2**	**2% O_2**
7553200	5587638.001	3.2E+08	109846078	96966.2	289217.2039	30946240.56	53450877.81	2182538.55	981621.586	4.48E+08	92024198.6
7297216	5209784.087	3.2E+08	138407003	144652	234202.2112	30003994.1	53070837.1	2580773.01	1502054.931	5.03E+08	91421337.82
6546487	5197422.034	4.2E+08	94407529.28	112608	289878.0417	35589002.99	35095465.28	2012234.41	1406243.123	7.04E+08	119838556.7
6213978	5473932.581	2.7E+08	107479831.8	114449	304259.5691	41560792.89	30083827.3	2249896.92	1218532.542	6.03E+08	121692778

Table Ana	Whole cell acetyl-CoA		Table Ana	Whole cell citrate	
Column B	2% O_2		Column B	2% O_2	
vs.	vs,		vs.	vs,	
Column A	21% O_2		Column A	21% O_2	
Unpaired t test			Unpaired t test		
P value		0.0034	P value		0.0005
P value su	**		P value su	***	
Significan	Yes		Significan	Yes	
One- or tv	Two-tailed		One- or tv	Two-tailed	
t, df	t=4,677, df=6		t, df	t=6,796, df=6	
How big is the difference?			How big is the difference?		
Mean of c		6902720	Mean of c		333698290
Mean of c		5367194	Mean of c		112535111
Difference	-1535526 ± 328330		Difference	-221163180 ± 32543575	
95% confi	-2338921 to -732131		95% confi	-300794439 to -141531921	
R squarec		0.7847	R squarec		0.885
F test to compare variances			F test to compare variances		
F, DFn, D	10,39, 3, 3		F, DFn, D	11,33, 3, 3	
P value		0.086	P value		0.0765
P value su	ns		P value su	ns	
Significan	No		Significan	No	
Data analyzed			Data analyzed		
Sample si		4	Sample si		4
Sample si		4	Sample si		4

Table Ana	CYTO AC-COA		Table Analyzed	CYTO CITRATE	
Column B	2% O_2		Column B	2% O_2	
vs.	vs,		vs.	vs,	
Column A	21% O_2		Column A	21% O_2	
Unpaired t test			Unpaired t test		
P value		0.0001	P value		0.2506
P value su	***		P value summary	ns	
Significan	Yes		Significantly different (P < 0.0	No	
One- or tv	Two-tailed		One- or two-tailed P value?	Two-tailed	
t, df	t=8,821, df=6		t, df	t=1,271, df=6	
How big is the difference?			How big is the difference?		
Mean of c		117169	Mean of column A		34525008
Mean of c		279389	Mean of column B		42925252
Difference	162221 ± 18391		Difference between means (B	8400244 ± 6606884	
95% confi	117220 to 207221		95% confidence interval	-7766218 to 24566706	
R squarec		0.9284	R squared (eta squared)		0.2122
F test to compare variances			F test to compare variances		
F, DFn, D	2,406, 3, 3		F, DFn, Dfd	5,245, 3, 3	
P value		0.4898	P value		0.2068
P value su	ns		P value summary	ns	
Significan	No		Significantly different (P < 0.0	No	
Data analyzed			Data analyzed		
Sample si		4	Sample size, column A		4
Sample si		4	Sample size, column B		4

Table Analyz	MITO AC-COA		Table Anal	MITO CITRATE	
Column B	2% O_2		Column B	2% O_2	
vs.	vs,		vs.	vs,	
Column A	21% O_2		Column A	21% O_2	
Unpaired t test			Unpaired t test		
P value		0.001	P value		0.0002
P value sumn	**		P value su	***	
Significantly c	Yes		Significantl	Yes	
One- or two-t	Two-tailed		One- or tw	Two-tailed	
t, df	t=5,920, df=6		t, df	t=8,033, df=6	
How big is the difference?			How big is the difference?		
Mean of colu		2256361	Mean of cc		564354627
Mean of colu		1277113	Mean of cc		106244218
Difference be	-979248 ± 165417		Difference	-458110409 ± 57031310	
95% confider	-1384008 to -574487		95% confid	-597660998 to -318559821	
R squared (e		0.8538	R squared		0.9149
F test to compare variances			F test to compare variances		
F, DFn, Dfd	1,078, 3, 3		F, DFn, Df	45,17, 3, 3	
P value		0.9523	P value		0.0108
P value sumn	ns		P value su	*	
Significantly c	No		Significantl	Yes	
Data analyzed			Data analyzed		
Sample size,		4	Sample siz		4
Sample size,		4	Sample siz		4
