# Supplementary material for: Hypoxia promotes osteogenesis by facilitating acetyl‐CoA‐mediated mitochondrial–nuclear communication
Source: EMBO J. 2022 Oct 24;41(23):e111239. doi: 10.15252/embj.2022111239 (PMC9713713; doi:10.15252/embj.2022111239)
Supplement: Supplementary file 7 — Source Data for Figure 5 [file EMBJ-41-e111239-s005.pdf]

Panel 5C:Nile Red ±acetate and BTA

2% O2	21% O2	2% O2 BTA	21% O_2 ACETATE
5295	693	2847	1604
4455	851	1873	2423
3958	633	3097	1660
5332	575	2926	1012
5962	267	2835	

Number of families	1
Number of comparisons per family	6
Alpha	0.05

Holm-Šídák's multiple comparisons test	Mean Diff,	Below threshold?	Summary	Adjusted P Value	
2% O2 vs. 21% O2	4397	Yes	****	<0,0001	A-B
2% O2 vs. 2% O2 BTA	2285	Yes	****	<0,0001	A-C
2% O2 vs. 21% O_2 ACETATE	3326	Yes	****	<0,0001	A-D
21% O2 vs. 2% O2 BTA	-2112	Yes	****	<0,0001	B-C
21% O2 vs. 21% O_2 ACETATE	-1071	Yes	*	0.023	B-D
2% O2 BTA vs. 21% O_2 ACETATE	1041	Yes	*	0.023	C-D

Test details	Mean 1	Mean 2	Mean Diff,	SE of diff,	n1	n2	t	DF
2% O2 vs. 21% O2	5000	603.8	4397	351.2	5	5	12.52	15
2% O2 vs. 2% O2 BTA	5000	2716	2285	351.2	5	5	6.506	15
2% O2 vs. 21% O_2 ACETATE	5000	1675	3326	372.5	5	4	8.928	15
21% O2 vs. 2% O2 BTA	603.8	2716	-2112	351.2	5	5	6.013	15
21% O2 vs. 21% O_2 ACETATE	603.8	1675	-1071	372.5	5	4	2.875	15
2% O2 BTA vs. 21% O_2 ACETATE	2716	1675	1041	372.5	5	4	2.794	15

Panel 5E:scoring of acetyl_lysine signal (% cells) ±BTA, acetate

Mitochondrial

2% O_2	2% O2 BTA	21% O2	% O2 ACETATE
13.6	51.1	53.9	8.9
1.1	47.6	62.5	9.2
5.4	50	88.8	3.5

Number of families	1
Number of comparisons per family	6
Alpha	0.05

Holm-Šídák's multiple comparisons test	Mean Diff,	Below threshold?	Summary	Adjusted P Value	
2% O_2 vs. 2% O2 BTA	-42.87	Yes	**	0.0027	A-B
2% O_2 vs. 21% O2	-61.7	Yes	***	0.0003	A-C
2% O_2 vs. 21% O2 ACETATE	-0.5	No	ns	0.9517	A-D
2% O2 BTA vs. 21% O2	-18.83	No	ns	0.0908	B-C
2% O2 BTA vs. 21% O2 ACETATE	42.37	Yes	**	0.0027	B-D
21% O2 vs. 21% O2 ACETATE	61.2	Yes	***	0.0003	C-D

Test details	Mean 1	Mean 2	Mean Diff,	SE of diff,	n1	n2	t	DF
2% O_2 vs. 2% O2 BTA	6.7	49.57	-42.87	8.005	3	3	5.355	8
2% O_2 vs. 21% O2	6.7	68.4	-61.7	8.005	3	3	7.708	8
2% O_2 vs. 21% O2 ACETATE	6.7	7.2	-0.5	8.005	3	3	0.06246	8
2% O2 BTA vs. 21% O2	49.57	68.4	-18.83	8.005	3	3	2.353	8
2% O2 BTA vs. 21% O2 ACETATE	49.57	7.2	42.37	8.005	3	3	5.293	8
21% O2 vs. 21% O2 ACETATE	68.4	7.2	61.2	8.005	3	3	7.646	8

Nuclear

2% O2	2% O2 BTA	21% O2	21% O2 ACETATE
57.9	19	13.4	62.6
66.6	15.3	12.5	48
72.5	16.6	0	48.2

Number of famili	1
Number of comp	6
Alpha	0.05

Holm-Šídák's mu	Mean Diff,	Below threshold?	Summary	Adjusted P Value		
2% O2 vs. 2% O:	48.7	Yes	***		0.0001	A-B
2% O2 vs. 21% (	57.03	Yes	****	<0,0001		A-C
2% O2 vs. 21% (	12.73	No	ns		0.0982	A-D
2% O2 BTA vs. 2	8.333	No	ns		0.1704	B-C
2% O2 BTA vs. 2	-35.97	Yes	***		0.0006	B-D
21% O2 vs. 21%	-44.3	Yes	***		0.0002	C-D

Test details	Mean 1	Mean 2	Mean Diff,	SE of diff,	n1	n2	t	DF
2% O2 vs. 2% O:	65.67	16.97	48.7	5.533	3	3	8.802	8
2% O2 vs. 21% (	65.67	8.633	57.03	5.533	3	3	10.31	8
2% O2 vs. 21% (	65.67	52.93	12.73	5.533	3	3	2.301	8
2% O2 BTA vs. 2	16.97	8.633	8.333	5.533	3	3	1.506	8
2% O2 BTA vs. 2	16.97	52.93	-35.97	5.533	3	3	6.501	8
21% O2 vs. 21%	8.633	52.93	-44.3	5.533	3	3	8.007	8

Panel 5F: MFI of nuclear acetyl-Lysine signal

2% O2	2% O_2 BTA	21% O2	21% O_2 ACETATE
60.308	53.184	1.793	22.152
42.675	59.939	2.478	40.771
63.782	1.815	27.474	9.656
47.598	12.867	0.683	62.74
69.167	45.729	3.247	57.751
13.709	36.638	58.849	43.701
58.909	12.716	5.351	64.271
30.299	7.221	38.423	71.731
48.279	28.152	1.471	66.7
0.783	56.191	15.896	63.872
16.186	28.029	66.859	59.307
17.492	27.687	53.903	55.256
46.096	18.315	47.706	52.92
35.755	1.59	2.394	22.566
23.26	6.61	12.071	67.976
50.548	31.351	1.846	62.185
72.389	33.039	50.458	49.991
70.274	2.455	30.648	64.301
39.001	3.475	61.174	57.743
48.887	13.722	12.16	67.378
53.373	45.781	45.908	60.532
39.696	6.065	6.748	24.322
34.695	10.318	61.787	61.375
51.799	2.525	56.636	57.918
37.208	8.574	5.624	54.305
23.323	7.103	10.02	54.701
23.555	2.526	27.944	62.746
38.286	3.7	56.726	58.713
5.461	21.433	5.443	58.711
67.083	10.822	5.176	52.508
55.571	21.429	36.194	70.1
27.851	51.251	11.289	57.384
60.163	16.246	51.093	70.88
55.854	17.531	34.41	52.781
51.811	18.415	66.593	9.31
67.934	3.93	3.143	40.001
11.591	18.189	67.55	42.727
44.282	10.781	6.877	53.425
48.74	56.445	24.013	58.618
44.126	19.214	2.702	43.229
35.009	19.728	48.726	46.946
49.897	28.03	21.761	46.506
43.468	36.633	7.067	48.737
46.724	17.442	9.181	51.306
42.536	21.283		43.514
38.623	3.278		46.034
49.37	58.057		47.593
39.937	48.01		44.374
50.471	14.676		45.645
42.531	45.14		39.906
43.255	68.728		37.333
39.208	29.762		43.745
38.485	16.963		44.037
48.788	8.725		49.049
49.571	33.979		46.911
35.234	2.082		40.956
68.52	50.037		47.741
41.092	34.684		50.889
72.837	6.101		44.954
71.567	24.844		47.3
62.773	13.684		36.663
71.118	53.998		38.07
67.597	0.651		53.225
68.567	43.328		
8.877	2.757		
47.973	58.045		
63.387	63.285		
	6.898		
	25.008		
	6.261		
	49.637		

Number of f	1
Number of c	6
Alpha	0.05

Holm-Šídák	Mean Diff,	Below thres	Summary	Adjusted P Value	
2% O2 vs. 2	21.01	Yes	****	<0,0001	A-B
2% O2 vs. 2	18.77	Yes	****	<0,0001	A-C
2% O2 vs. 2	-4.677	No	ns	0.2697	A-D
2% O_2 BTA	-2.242	No	ns	0.5225	B-C
2% O_2 BTA	-25.69	Yes	****	<0,0001	B-D
21% O2 vs.	-23.45	Yes	****	<0,0001	C-D

Test details	Mean 1	Mean 2	Mean Diff,	SE of diff,	n1	n2	t	DF
2% O2 vs. 2	45.3	24.29	21.01	3.108	67	71	6.76	241
2% O2 vs. 2	45.3	26.53	18.77	3.541	67	44	5.301	241
2% O2 vs. 2	45.3	49.98	-4.677	3.202	67	63	1.461	241
2% O_2 BTA	24.29	26.53	-2.242	3.501	71	44	0.6404	241
2% O_2 BTA	24.29	49.98	-25.69	3.158	71	63	8.133	241
21% O2 vs.	26.53	49.98	-23.45	3.585	44	63	6.54	241

Panel 5H: Alizarin Red ± acetate

2% O_2	21% O_2	21% O2 acetate
92723	6864	58822
92022	18096	62990
95008	27970	57605
71988	12386	44162
77799	25562	

Number of families	1
Number of comparisons per family	3
Alpha	0.05

Holm-Šídák's multiple comparisons test	Mean Diff,	Below threshold?	Summary	Adjusted P Value	
2% O_2 vs. 21% O_2	67732	Yes	****	<0,0001	A-B
2% O_2 vs. 21% O_2 acetate	30013	Yes	***	0.0005	A-C
21% O_2 vs. 21% O_2 acetate	-37719	Yes	***	0.0002	B-C

Test details	Mean 1	Mean 2	Mean Diff,	SE of diff,	n1	n2	t	DF
2% O_2 vs. 21% O_2	85908	18176	67732	5839	5	5	11.6	11
2% O_2 vs. 21% O_2 acetate	85908	55895	30013	6193	5	4	4.846	11
21% O_2 vs. 21% O_2 acetate	18176	55895	-37719	6193	5	4	6.09	11
