# Supplementary material for: Hypoxia promotes osteogenesis by facilitating acetyl‐CoA‐mediated mitochondrial–nuclear communication
Source: EMBO J. 2022 Oct 24;41(23):e111239. doi: 10.15252/embj.2022111239 (PMC9713713; doi:10.15252/embj.2022111239)
Supplement: Supplementary file 8 — PDF+ [file EMBJ-41-e111239-s002.pdf]

Article

SOURCE DATA | TRANSPARENT PROCESS | OPEN ACCESS

THE EMBO JOURNAL

Hypoxia promotes osteogenesis by facilitating acetyl-CoA-mediated mitochondrial–nuclear communication

Andromachi Pouikli[1,2], Monika Maleszewska[1,†], Swati Parekh[1], Ming Yang[2], Chrysa Nikopoulou[1], Juan Jose Bonfiglio[3,‡], Constantine Mylonas[1,§], Tonantzi Sandoval[1], Anna-Lena Schumacher[4], Yvonne Hinze[5], Ivan Matic[2,3], Christian Frezza[2] & Peter Tessarz[1,2,*]

Abstract

Bone-derived mesenchymal stem cells (MSCs) reside in a hypoxic niche that maintains their differentiation potential. While hypoxia (low oxygen concentration) was reported to critically support stem cell function and osteogenesis, the molecular events triggering changes in stem cell fate decisions in response to normoxia (high oxygen concentration) remain elusive. Here, we study the impact of normoxia on mitochondrial–nuclear communication during stem cell differentiation. We show that normoxia-cultured murine MSCs undergo profound transcriptional alterations which cause irreversible osteogenesis defects. Mechanistically, high oxygen promotes chromatin compaction and histone hypo-acetylation, particularly on promoters and enhancers of osteogenic genes. Although normoxia induces metabolic rewiring resulting in elevated acetyl-CoA levels, histone hypo-acetylation occurs due to the trapping of acetyl-CoA inside mitochondria owing to decreased citrate carrier (CiC) activity. Restoring the cytosolic acetyl-CoA pool remodels the chromatin landscape and rescues the osteogenic defects. Collectively, our results demonstrate that the metabolism–chromatin–osteogenesis axis is perturbed upon exposure to high oxygen levels and identifies CiC as a novel, oxygen-sensitive regulator of the MSC function.

Keywords histone acetylation; hypoxia; mesenchymal stem cells; metabolism; osteogenesis

Subject Categories Chromatin, Transcription & Genomics; Metabolism; Stem Cells & Regenerative Medicine

DOI 10.15252/embj.2022111239 | Received 22 March 2022 | Revised 17 September 2022 | Accepted 23 September 2022 | Published online 24 October 2022

The EMBO Journal (2022) 41: e111239

Introduction

Mesenchymal stem cells (MSCs) are somatic stem cells that reside in various embryonic and adult tissues and can differentiate into bone, fat, cartilage, tendon and other organ progenitor cells, contributing to the organization and maintenance of tissue integrity (Gomez-Salazar *et al*, 2020). Given their role in maintaining tissue homeostasis, it is not surprising that MSCs have been extensively used in regenerative medicine, as a promising tool during stem cell therapies (Parekkadan & Milwid, 2010). However, their clinical use requires extended *in vitro* expansion, which alters their physiological properties. More precisely, the absence of niche molecular signals and the lack of interaction with neighbouring cells have been found to affect several parameters of the stem cell behaviour during *in vitro* culture of MSCs (Gomez-Salazar *et al*, 2020). Furthermore, oxygen concentration has a dramatic impact on stem cell biology; indeed, the MSC niche within the medullary cavity of the bone is characterized by low oxygen tension, ranging from 1.3 to 4.2% O_2 (Spencer *et al*, 2014). However, MSCs used in research studies as well as in therapeutics are usually cultured under normoxic conditions, i.e. 21% O_2, which significantly affects their activity (Parekkadan & Milwid, 2010). In fact, several reports have demonstrated that high oxygen impacts stem cell fate decisions and impairs the commitment of MSCs to specific lineages (Fehrer *et al*, 2007; Buravkova *et al*, 2014). However, we still lack a comprehensive understanding of the molecular, cell-intrinsic mechanisms linking oxygen concentration to MSC function.

Cells rewire their metabolism in response to oxygen availability, and these metabolic changes influence the stem cell differentiation capacity (Leijten *et al*, 2014); in turn, efficient differentiation of MSCs into specific cell types is directly associated with oxygen levels and requires profound metabolic rearrangements, such as

1 Max Planck Research Group “Chromatin and Ageing”, Max Planck Institute for Biology of Ageing, Cologne, Germany
2 Cologne Excellence Cluster on Stress Responses in Ageing-Associated Diseases (CECAD), Cologne, Germany
3 Research Group “Proteomics and ADP-Ribosylation Signaling”, Max Planck Institute for Biology of Ageing, Cologne, Germany
4 FACS & Imaging Core Facility, Max Planck Institute for Biology of Ageing, Cologne, Germany
5 Metabolomics Core Facility, Max Planck Institute for Biology of Ageing, Cologne, Germany
*Corresponding author. Tel: +49 221 37970680; E-mail: ptessarz@age.mpg.de
†Present address: CareDx, Inc., San Francisco, CA, USA
‡Present address: Roche Pharma Research and Early Development, Munich, Germany
§Present address: Novartis Institutes for BioMedical Research, Cambridge, MA, USA

© 2022 The Authors. Published under the terms of the CC BY 4.0 license.

down-regulation of glycolysis and induction of oxidative phosphorylation (OxPhos) (Shyh-Chang *et al*, 2013). Furthermore, metabolism is tightly linked to the epigenome, via central metabolites that serve as co-factors and substrates for epigenetic enzymes (Lu & Thompson, 2012; Etchegaray & Mostoslavsky, 2016; Dai *et al*, 2020). Therefore, the oxygen-driven changes in cell metabolism could dramatically alter the epigenome of stem cells. Notably, the epigenetic landscape determines the transcriptional output, which regulates stem cell fate decisions and controls stem cell identity. Thus, characterizing in depth that the metabolism–chromatin–stemness axis could help develop clinical strategies to intervene in MSC fate decisions, aiming at enhancing their therapeutic potential.

Here, we used bone-derived MSCs to investigate the role of oxygen in the regulation of stem cell fate decisions. We found that high oxygen levels lead to defective osteogenesis and impaired MSC function due to changes in the transcriptional profile. Mechanistically, high oxygen concentration induces epigenetic alterations characterized by chromatin compaction and histone hypo-acetylation, particularly on promoters and enhancers of osteogenic genes. Despite the oxygen-driven metabolic rewiring which results in the production of high acetyl-CoA levels, this remains trapped inside the mitochondria of normoxia-cultured cells, leading to histone hypo-acetylation. Strikingly, accumulation of acetyl-CoA within mitochondria occurs due to impaired citrate carrier (CiC) activity and re-distribution of mitochondrial acetyl-CoA to the cytosol, either via exogenous CiC overexpression or by acetate supplementation, restores chromatin plasticity and the osteogenic differentiation potential. Remarkably, similar changes were observed in aged MSCs, suggesting that high oxygen mimics the physiological process of ageing with regards to the acetyl-CoA-mediated mito-nuclear communication in the regulation of stem cell differentiation (Pouikli *et al*, 2021).

Results

Normoxia impairs osteogenesis by altering the MSC transcriptome

In order to investigate the role of oxygen concentration in the metabolism–chromatin–stem cell fate axis, we used MSCs freshly isolated from the bone tissue. We isolated and cultured MSCs based on a protocol that allowed purification of a highly homogeneous and tri-lineage potent population of MSCs which expressed increased levels of the Sca-1 and PDGFRa markers, maintained throughout the *in vitro* cell culture period (Pouikli *et al*, 2021). The sorted MSC population was then divided into two groups; one was maintained at hypoxic conditions, whereas we shifted the other one to normoxia, as outlined in Fig 1A. In order to allow cells to adapt in the high oxygen environment, we performed all experiments 7 days after transferring cells to normoxia, thus focusing on the long-term effects of normoxia in stem cell function. Culturing cells under normoxic conditions did not impact their adipogenic differentiation capacity (Fig 1B and C). However, we found that normoxia-cultured cells exhibited profound defects in their osteogenic differentiation potential (Fig 1D and E), indicating that normoxia promoted skewed adipogenic differentiation at the expense of osteogenesis. Furthermore, we demonstrated that normoxia impaired osteogenesis permanently, since shifting MSCs back to hypoxia, after culture under normoxic conditions for 7 days, did not rescue the impaired osteogenic differentiation (Fig 1D and E). Interestingly, the transcriptomic profile of MSCs was also heavily influenced by the high oxygen levels, with normoxia-cultured cells clustering separately from those maintained permanently under hypoxic conditions (Fig 1F). In line to reduced osteogenesis under normoxic conditions, GO enrichment analysis revealed that the down-regulated genes in normoxia were involved in processes and signalling cascades associated with bone development and osteogenesis (Fig 1G). These findings suggest that high oxygen levels induced profound alterations in the transcriptional output of MSCs leading to reduced osteogenesis.

High oxygen levels lead to global chromatin compaction

Given that chromatin architecture is involved in the regulation of gene transcription, we next sought to investigate whether the normoxia-driven changes in the transcriptional profile were dictated by alterations in the chromatin landscape. Interestingly, two recent reports showed profound changes in chromatin accessibility following a switch in oxygen levels (Li *et al*, 2020; Batie *et al*, 2022). Since these two studies report opposite effects of hypoxia on chromatin accessibility, we next performed ATAC sequencing (ATAC-seq) on hypoxia- and normoxia-cultured cells (Fig EV1A and B) to investigate how oxygen availability alters the chromatin accessibility profile of MSCs. PCA analysis showed that cells were clearly distinct and separated based exclusively on the oxygen conditions where they were cultured (Fig 2A). Furthermore, we identified, in total 81,393 accessible sites of which 23,571 changed significantly (FDR < 0.01) in accessibility upon shift to atmospheric oxygen (Fig 2B); in particular, 7,857 sites became more open, whereas 15,714 sites became more compact. Thus, we reported that normoxia led to global chromatin compaction in bone-derived MSCs (Fig 2C). We further analysed differences in the chromatin structure and plotted accessibility over the transcription start site (TSS) as a metaplot. We observed that gene promoters of cells exposed to high oxygen levels exhibited a strong decrease in chromatin accessibility (Fig 2D). To validate this result, we employed NucleoATAC, an algorithm that allows the precise mapping of nucleosomes from ATAC-seq datasets (Schep *et al*, 2015). Plotting nucleosome occupancy over the TSS of all protein-coding genes confirmed that atmospheric oxygen resulted in higher nucleosome density around the promoter region, but more importantly, confirmed the reduced chromatin accessibility at the TSS (Fig 2E). Feature distribution analysis, applying promoter proximity criteria to call gene promoters and using previously published enhancer regions in MSCs (Pouikli *et al*, 2021) to identify potential enhancers, confirmed that the vast majority of closing peaks in normoxia-cultured cells were found in gene regulatory elements, i.e. promoters and enhancers (Fig 2F). This strong decrease in chromatin accessibility at the TSS was also visible on a single gene level. For instance, promoters of genes important for osteogenesis, such as *Wnt1*, *Bglap*, *Bmp2* and *Dlx3*, showed decreased chromatin accessibility in normoxia-cultured cells (Fig 2G). Overall, osteogenic promoters were dramatically remodelled in response to high oxygen levels, indicating that the transcriptional and functional osteogenesis defects were regulated on the chromatin level.

 © 2022 The Authors

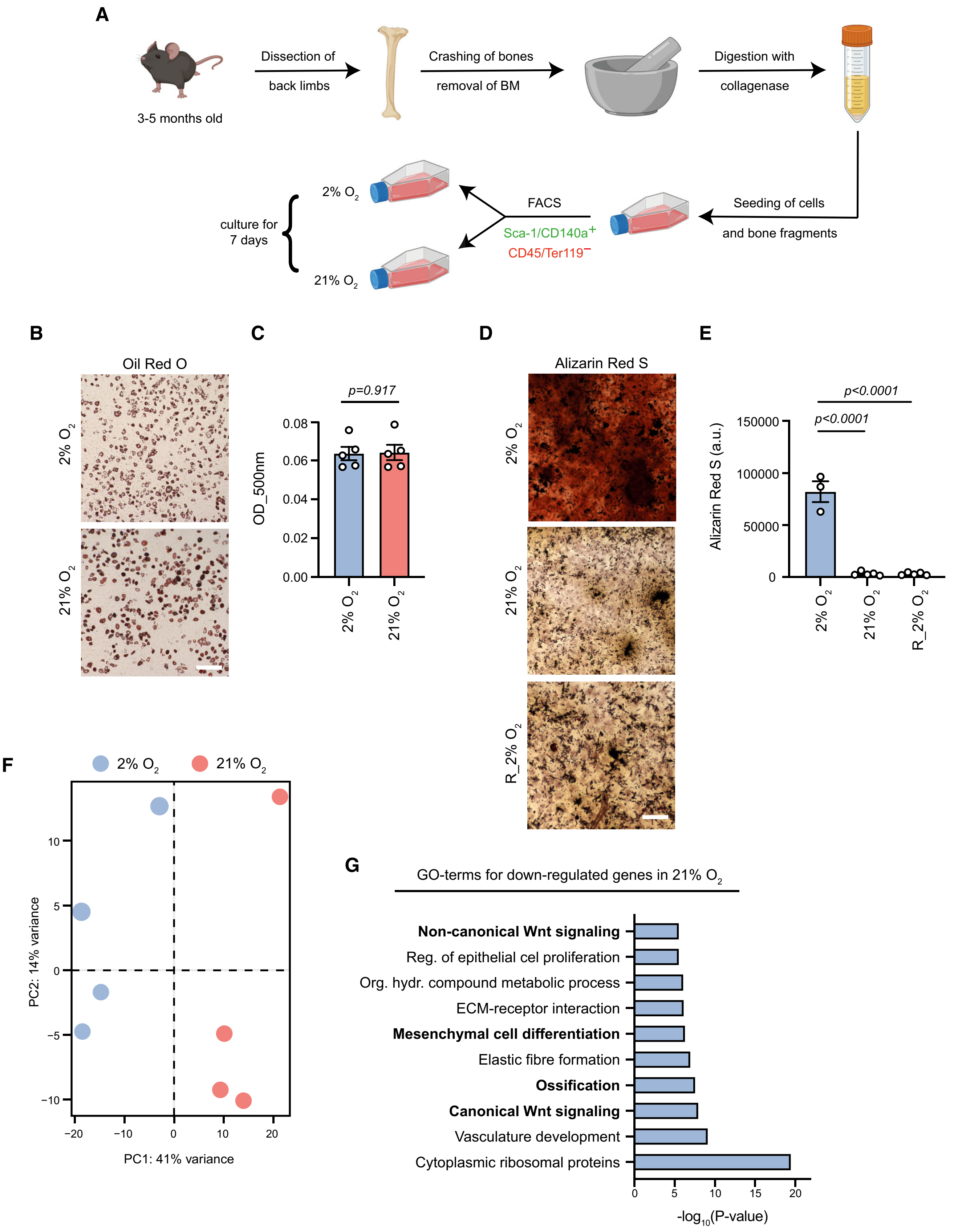
A
3-5 months old
Dissection of
back limbs
Crashing of bones
removal of BM
Digestion with
collagenase
Seeding of cells
and bone fragments
FACS
Sca-1/CD140a+
CD45/Ter119−
2% O2
21% O2
culture for
7 days
B
Oil Red O
2% O2
21% O2
C
p=0.917
OD_500nm
D
Alizarin Red S
R_2% O2
E
p<0.0001
p<0.0001
Alizarin Red S (a.u.)
F
PC1: 41% variance
PC2: 14% variance
G
GO-terms for down-regulated genes in 21% O2
Non-canonical Wnt signaling
Reg. of epithelial cel proliferation
Org. hydr. compound metabolic process
ECM-receptor interaction
Mesenchymal cell differentiation
Elastic fibre formation
Ossification
Canonical Wnt signaling
Vasculature development
Cytoplasmic ribosomal proteins
-log10(P-value)

Figure 1.

◀ **Figure 1. Normoxia changes the transcriptional output to suppress osteogenesis.**

A Schematic representation demonstrating the isolation protocol and the culture conditions of bone MSCs isolated from the back limbs of young (~3–5 months old) mice. After collection of the limbs, clean bones were cut into small pieces, which were then treated with collagenase for 1 h at 37°C. Cells and bone fragments were seeded in flasks and incubated for 10 days under 2% O_2. On day 10, cell sorting was performed using flow cytometry and selecting the $CD45^{-}$/$Ter\text{-}119^{-}$/$Sca\text{-}1^{+}$/$CD140a^{+}$ mesenchymal stem cell population. The isolated population was then split into two groups: one was transferred back to hypoxia, whereas the other one was shifted to normoxia. Cells were cultured under these conditions for 7 days.

B, C Representative images (B) and quantification (C) of Oil Red O staining of hypoxia- and normoxia-cultured cells, 9 days after induction of adipogenesis. $n = 5$ biologically independent replicates. Results are shown as mean ± SEM and statistical significance was determined using a two-sided unpaired *t*-test.

D, E Representative images (D) and quantification (E) of Alizarin Red S staining of hypoxic, normoxic and reversed hypoxic (R_2% O_2) cells, 12 days after induction of osteogenesis. Cells were exposed to 21% O_2 for 7 days and then moved back to 2% O_2, where osteogenesis was induced after 4 days. $n = 3$ for hypoxic cells and $n = 5$ biologically independent experiments for normoxic and reversed-hypoxic cells, and merged results are shown in (E). Results are shown as mean ± SEM and statistical significance was determined with ordinary one-way ANOVA, using Holm–Sidak's multiple-comparisons test.

F Principal component analysis (PCA) plot showing clustering of hypoxia- and normoxia-cultured cells after RNA-seq. $n = 4$ biologically independent replicates.

G GO enrichment analysis for down-regulated genes upon exposure to normoxia, as identified by RNA-seq.

Data information: Scale bars, 500 μm.
Source data are available online for this figure.

Normoxia results in decreased histone acetylation on enhancers of osteogenic genes

To further investigate the molecular underpinnings of the normoxia-associated chromatin compaction, we compared histone acetylation levels between hypoxia- and normoxia-cultured cells. Histone acetylation neutralizes the positive charge of the side chain of histones and weakens the contact between histones and DNA (Tessarz & Kouzarides, 2014). Thus, it plays a fundamental role in the regulation of chromatin accessibility. Therefore, we investigated if alterations in the histone acetylation profile contributed to normoxia-induced changes in chromatin accessibility. We initially followed an unbiased approach and performed quantitative SILAC–mass spectrometry analysis (SILAC–MS), as outlined in Fig 3A, using heavy-labelled commercially available MSCs as SILAC spike-in standard. Although the coverage of the detected modifications was not extensive due to the low cell number, our results demonstrated that histone methylation remained largely unaffected (Fig 3B) except for H3K9me2, a transcriptional repressive mark which was increased in normoxic cells, in accordance with their compacted chromatin state. By contrast, we found that normoxic MSCs displayed a strong decrease in the acetylation of several histone residues (Fig 3B). In line with this, the culture of MSCs under normoxic conditions led to lower histone H3 acetylation levels, as evidenced by immunofluorescence experiments (Fig 3C and D).

Given the strong impact of high oxygen levels on enhancer accessibility (Fig 2F) and the tight link between histone acetylation and chromatin accessibility, we next studied the oxygen-induced changes in the regulatory elements of the affected genes. First, we used hypoxia- and normoxia-cultured cells to generate ChIP-seq data for H3K27 acetylation (H3K27ac) and H3K4 tri-methylation (H3K4me3), two marks associated with active promoters and enhancers (Fig EV1C–E). H3K27ac distribution was dynamically remodelled upon shift of cells to normoxia, with specific DNA regions either gaining or losing H3K27ac signal (Fig 3E). In contrast, H3K4me3 distribution was not affected by the change in the oxygen concentration (Fig EV1D and E). Focusing on the H3K27ac mark whose abundance changed in response to high oxygen levels, we next annotated genes based on the "closest distance" method (Moore *et al*, 2020). Interestingly, loss of H3K27ac signal in normoxia-cultured MSCs was associated with genes involved in MSC function. More precisely, genes whose promoters and enhancers lost H3K27ac mark were associated with skeletal development and Wnt signalling (Fig 3F), while the increase in the H3K27ac abundance was found close to genes involved in signalling cascades, such as the MAP kinase and the Rho GTPase cycle pathways (Fig 3G). An example of a gene involved in osteogenesis which lost both chromatin accessibility and H3K27ac abundance on potential enhancer in normoxia is shown for *Sp7* (Fig 3H). Together our data demonstrate that high oxygen resulted in histone hypo-acetylation and chromatin condensation which led to lower osteogenic differentiation potential.

Normoxia rewires energy metabolism favouring acetyl-CoA production

Our findings regarding lower histone acetylation in normoxia-cultured MSCs prompted us to investigate how the histone acetylation profile was established in hypoxic and normoxic cells and whether other cellular processes were involved in this phenotype. Thus, we next sought to characterize the metabolic profile of hypoxia- and normoxia-cultured MSCs. Consistent with previous reports, we showed that hypoxia-cultured cells relied heavily on glycolysis for energy production (Fig EV2A–E), whereas high oxygen resulted in stimulation of the mitochondrial activity and up-regulation of OxPhos (Fig EV2F–H). Unexpectedly, we found that this profound metabolic rewiring led to increased total acetyl-CoA levels in normoxic MSCs (Fig 3I). Given that the acetyl-CoA availability directly influences levels of histone acetylation, this result was in contradiction to the histone hypo-acetylation observed in MSCs from normoxia-cultured cells. Therefore, we set out to investigate further where the higher levels of acetyl-CoA originated from and what they were used for. One main metabolic pathway that heavily generates and consumes acetyl-CoA is lipid metabolism. To understand if the lipid content changed in response to high oxygen levels, we stained cells with Nile Red to visualize neutral lipids. We found that normoxic MSCs exhibited a much weaker lipid staining, indicating lower content of neutral lipids (Fig EV3A and B). This observation was also confirmed with electron microscopy data that showed a strong decrease in the amount of lipid droplets (LDs) in normoxic MSCs (Fig EV3A). The lower LD content could be explained both by lower fatty acid biosynthesis and by increased

 © 2022 The Authors

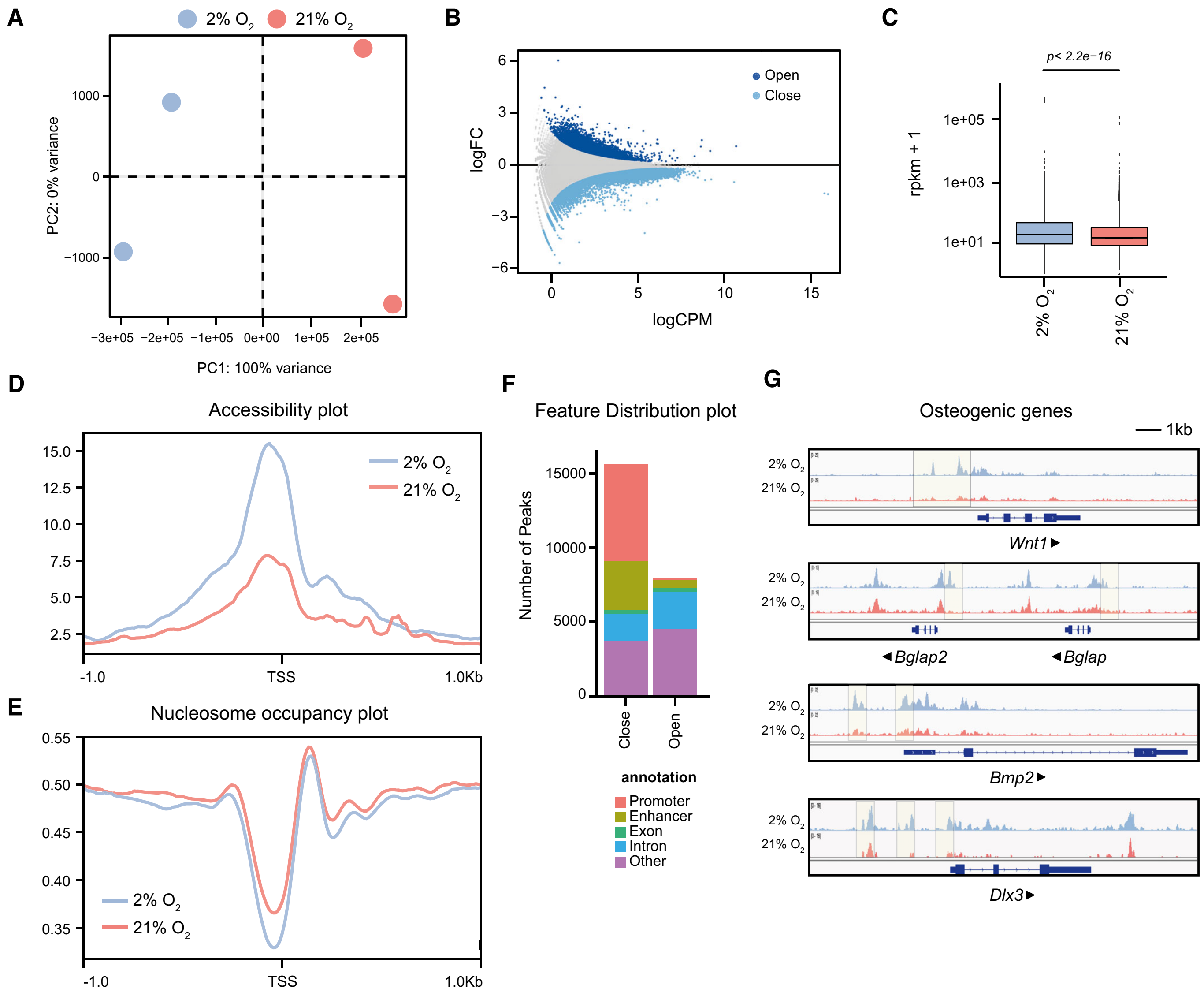

Figure 2. Normoxia leads to reduced chromatin accessibility.

A Principal component analysis (PCA) plot showing clustering of hypoxia- and normoxia-cultured cells after ATAC-seq.

B MA plot showing opening and closing peaks upon shift to high oxygen, as determined by ATAC-seq.

C Overall genome accessibility expressed as RPKM values measured by ATAC-seq. The y-axis is scaled to log10 for visualization purposes (n = 81,393). Boxplots consist of the median (central line), the 25th and the 75th percentiles (box) and the highest/lowest value within 1.5 × interquartile range of the box (whiskers).

D Metaplot of ATAC-seq reads over the TSS of all protein-coding genes.

E NucleoATAC metaplot to map the position of all nucleosomes around the TSS of all protein-coding genes.

F Feature distribution plot showing that most differentially accessible sites fall into promoters and enhancers.

G Integrative genomics viewer (IGV) browser views showing ATAC-seq read density in hypoxia- and normoxia-cultured MSCs, near the *Wnt1*, *Bglap*, *Bmp2* and *Dlx3* gene promoters. Shaded regions demonstrate accessibility differences between hypoxia- and normoxia-cultured cells. Arrows indicate the direction of transcription.

Data information: n = 2 biologically independent experiments.

lipid consumption through β-oxidation. Focusing on potential alterations in the lipid biogenesis pathway and comparing the levels of enzymes involved in the *de novo* synthesis of fatty acids, we showed that the protein levels of the master regulator of lipogenesis SREBP1 and of the key-lipogenic enzymes FASN and ACC1 were strongly decreased in normoxic cells (Fig EV3C and D). This result suggested that acetyl-CoA entrance into the lipid biogenesis pathway was inhibited in response to high oxygen levels. In sum, our findings demonstrate that normoxia influenced central metabolic pathways, leading to increased production of acetyl-CoA which accumulated in the cells.

Acetyl-CoA is trapped inside the mitochondria of normoxic cells

Despite the fact that normoxic cells contained higher total acetyl-CoA levels (Fig 3I), they exhibited global loss of histone acetylation

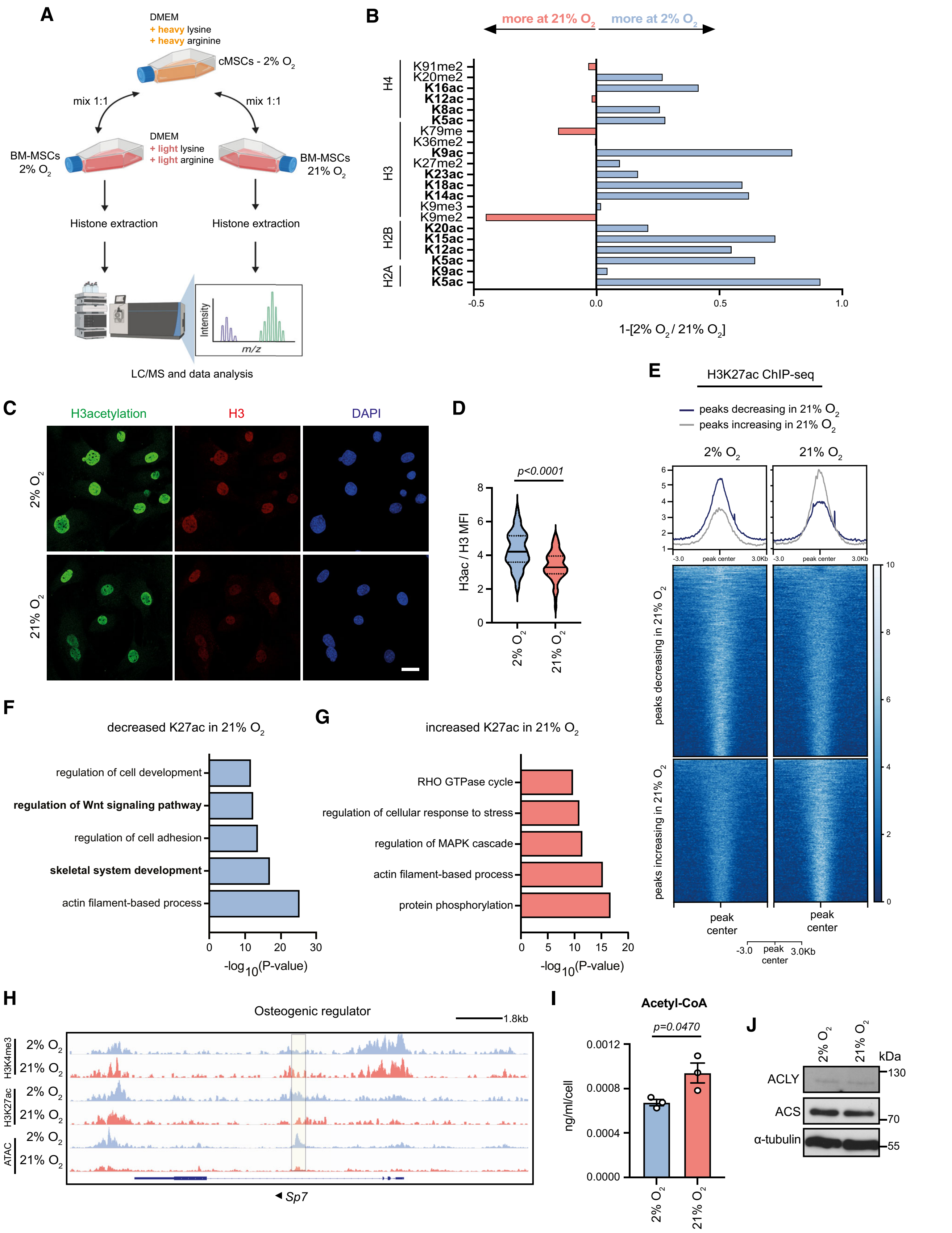

Figure 3.

 © 2022 The Authors

Figure 3. Normoxia results in histone hypo-acetylation on enhancers of osteogenic genes.

A, B Schematic representation demonstrating the protocol followed in the SILAC-MS experiment, where hypoxia- and normoxia-cultured cells were mixed with heavy-labelled commercial MSCs and were then subjected to histone extraction and liquid chromatography–mass spectrometry (LC/MS) analysis (A). Levels of the detected histone modifications in hypoxic and normoxic MSCs (B). $n = 2$ biologically independent experiments.

C, D Representative images (C) and quantification (D) of mean fluorescence intensity (MFI) after immunostaining against H3ac and H3 of hypoxic and normoxic cells. The mean fluorescence intensity (MFI) of histone H3 was used as internal control for normalization. Nuclei were stained with DAPI. Quantification of H3ac/H3 MFI from $n = 67$ hypoxic and $n = 56$ normoxic individual cells from a representative experiment of three biologically independent experiments is shown in (D). Distribution of data points in (D) is shown as a violin plot, where the mean is indicated by a solid line and the quartiles are indicated with dashed lines. Results are shown as mean ± SEM and statistical significance was determined using a two-sided unpaired *t*-test.

E Heatmap of H3K27ac density, centred around peak centre in hypoxia- and normoxia-cultured cells. Regions that lose or gain H3K27ac are shown for each condition. $n = 2$ biologically independent experiments.

F, G GO enrichment analysis based on nearest genes of H3K27ac peaks as shown in (E), depending on either lost or gained H3K27ac signal.

H Integrative genomics viewer (IGV) browser views showing ATAC-seq, H3K4me3 and H3K27ac read density in hypoxia- and normoxia-cultured MSCs, likely in the *Sp7* gene enhancer. The shaded region demonstrates differences in H3K27ac and ATAC-seq read abundance between hypoxia- and normoxia-cultured cells. Arrow indicates the direction of transcription.

I LC/MS analysis of acetyl-CoA in hypoxia- and normoxia-cultured cells. $n = 3$ biologically independent experiments. Results are shown as mean ± SEM and statistical significance was determined using a two-sided unpaired *t*-test.

J Representative immunoblots for ACS and ACLY in hypoxia- and normoxia-cultured cells. A-tubulin was used as a loading control. $N = 3$ biologically independent experiments.

Data information: Scale bar, 25 μm.
Source data are available online for this figure.

(Fig 2). Therefore, we then investigated how this apparent contradiction could be explained. One potential reason for the reduced histone acetylation in normoxia-cultured cells could be a decrease in the cytosolic pool of acetyl-CoA. However, we found that levels of the enzymes ATP-citrate lyase (ACLY) and acetyl-CoA synthetase (ACS), which are both involved in the cytoplasmic/nuclear generation of acetyl-CoA (Wellen *et al*, 2009; Mews *et al*, 2017), were stable upon switch of MSCs to normoxic conditions (Fig 3J). Hence, we speculated that in normoxia-cultured cells, acetyl-CoA might be trapped inside mitochondria leading to an impaired cytosolic/nuclear acetyl-CoA pool that could in turn result in histone hypo-acetylation. To test this hypothesis, we stained hypoxia- and normoxia-cultured cells with the acetyl-lysine antibody, which recognizes all acetylated lysine residues, and we used TOMM20 as a counterstain for mitochondria. Of note, when acetyl-CoA accumulates in mitochondria in excessive amounts, it promotes the non-enzymatic acetylation of mitochondrial proteins (James *et al*, 2017). Surprisingly, we found that upon culture of MSCs under high oxygen levels, mitochondria accumulated more acetylated proteins and the signal of the acetyl-lysine antibody shifted from the nucleus to mitochondria (Fig 4A and B), suggesting that in normoxia-cultured MSCs acetyl-CoA is trapped within the mitochondria. To further validate this finding, we performed metabolomic analysis after subcellular fractionation. Notably, primary bone-derived MSCs represent a rare stem cell population and are isolated in numbers insufficient for big-scale experiments. Therefore, for our metabolomic analysis we used commercially available MSCs, which have adapted to normoxia. Of note, when cultured under normoxic conditions, these cells displayed impaired flux of acetyl-CoA from mitochondria to the cytosol/nucleus (Fig EV4A and B), similarly to primary MSCs. Using these cells, we followed a published protocol outlined in Fig 4C, which allows rapid subcellular fractionation coupled with metabolite extraction and immunoblotting (Lee *et al*, 2019). Importantly, since we were interested in measuring acetyl-CoA and citrate, both of which are labile molecules that can be easily diffused during the fractionation process, this was the fastest protocol available for quick—yet efficient—subcellular fractionation. Indeed, this method yielded "pure" fractions while maintaining mitochondrial quality, as confirmed by immunoblot analysis against TOMM20, α-tubulin and MT-CO1 proteins (Fig EV4C). Methanol:acetonitrile-based extraction of metabolites and downstream LC/MS analysis revealed that the metabolic profiles of normoxic and hypoxic subcellular fractions were largely different, with normoxic samples clustering separately from hypoxic (Fig EV4D). This approach also showed that normoxia-cultured MSCs, although they contained higher total levels of acetyl-CoA and citrate, exhibited lower cytosolic and increased mitochondrial levels of these two metabolites (Fig 4D). Therefore, our findings suggest that normoxia-cultured MSCs exhibited strong differences in the subcellular acetyl-CoA distribution, which was higher inside mitochondria, confirming the acetyl-CoA trapping inside these organelles.

Low CiC activity leads to acetyl-CoA accumulation in the mitochondria of normoxic MSCs

Recently, we demonstrated that aged MSCs exhibit a similar phenotype to normoxic MSCs and we identified that lower levels of citrate carrier (CiC) are responsible for the impaired export of acetyl-CoA from the mitochondria to the cytosol (Pouikli *et al*, 2021). Therefore, we next compared CiC levels between hypoxia- and normoxia-cultured cells. Expression levels of the CiC-encoding gene, *Slc25a1*, and CiC protein levels were comparable between the two oxygen conditions (Fig EV5A and B), suggesting that CiC levels were not influenced by oxygen concentration and thus are not involved in the described phenotype. However, we next investigated whether changes in the acetyl-CoA-exporting CiC function could be responsible for the trapping of acetyl-CoA within mitochondria. For this, we initially modulated pharmacologically the CiC activity, either by supplementing normoxic cells with the acetyl-CoA precursor sodium acetate or by inhibiting the CiC function in hypoxic cells using the BTA inhibitor (Fig 5A). We found that BTA treatment led to repression of lipogenesis (Fig 5B and C) and accumulation of acetyl-CoA within the mitochondria of hypoxic cells (Fig 5D–F), thus mimicking

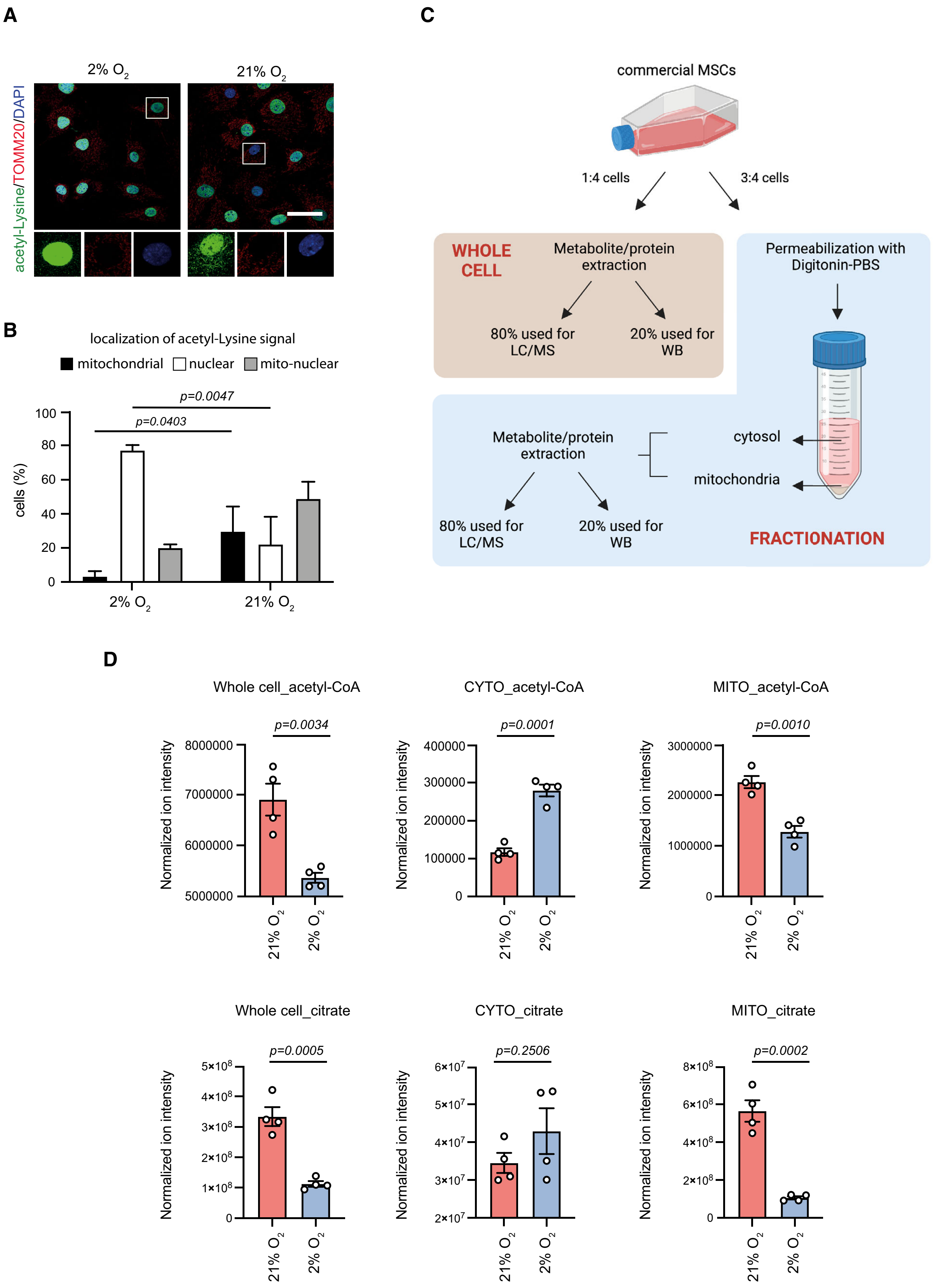

Figure 4.

© 2022 The Authors

Figure 4. Trapping of acetyl-CoA inside mitochondria of normoxia-cultured MSCs.

A, B Representative images after immunostaining of hypoxia- and normoxia-cultured cells against acetyl-lysine and TOMM20 (A), and assessment of the acetyl-lysine signal localization, after manual assignment into three categories: exclusively nuclear, exclusively mitochondrial and nuclear/mitochondrial (B). Nuclei were stained with DAPI. N = 3 biologically independent experiments. Results are shown as mean ± SEM and statistical significance was determined using a two-sided unpaired *t*-test. In magnified insets, the intensity of the acetyl-lysine signal was adjusted similarly to all samples, for visualization purposes.

C, D Schematic representation demonstrating the protocol followed to isolate mitochondrial and cytosolic fractions of normoxia- and hypoxia-cultured commercial MSCs followed by metabolite extraction (C) and levels of acetyl-CoA and citrate measured in each fraction of normoxic and hypoxic cells (D). n = 4 biologically independent experiments. Results are shown as mean ± SEM and statistical significance was determined using a two-sided unpaired *t*-test.

Data information: Scale bar, 25 μm.
Source data are available online for this figure.

normoxia. By contrast, acetate supplementation of normoxic cells was sufficient to restore lipid biogenesis and nuclear localization of acetyl-CoA (Fig 5B–F), reverting the effects of high oxygen levels and mimicking hypoxic conditions.

To critically test whether CiC was indeed the mechanistic target leading to acetyl-CoA accumulation in mitochondria, we expressed the FLAG-tagged CiC exogenously in normoxic cells, using lentiviral transduction. Overexpressed CiC localized properly to mitochondria, as evidenced by the co-localization between the FLAG-tagged CiC protein and the mitochondrial membrane protein TOMM20 (Fig EV5C). Strikingly, we found that exogenous CiC overexpression in normoxic cells was sufficient to rescue the acetyl-CoA export and the cytosolic/nuclear acetyl-CoA abundance (Fig EV5D–F), highlightening the central role of CiC in the maintenance of the cytosolic acetyl-CoA levels.

Together, our results strongly suggest that impaired CiC activity in normoxia-cultured MSCs was responsible for the acetyl-CoA trapping within mitochondria.

Decreased CiC activity leads to osteogenic defects in normoxic MSCs

Last, we sought to investigate the functional consequences of the normoxia-induced loss of CiC activity. Given that the efficient export of acetyl-CoA from mitochondria to the cytosol is indispensable for lipogenesis, which is in turn required for membrane synthesis during proliferation and efficient commitment to osteocytes, we speculated that the decrease in CiC function was responsible for the observed changes in osteogenesis. To test this hypothesis, we pretreated normoxic cells with acetate and we then induced osteogenic differentiation. Impressively, acetate pre-treatment of normoxic cells rescued their ability to differentiate into osteoblasts (Fig 5G and H). Importantly, given that acetate was not present during the differentiation process and that the pre-treatment was sufficient to increase the osteogenic differentiation potential, our data suggest that pretreatment with acetate reset the chromatin landscape to make it more permissive for differentiation.

Collectively, our results show that the metabolism–chromatin–stemness axis is heavily affected by the oxygen levels and identify CiC as a novel, oxygen-sensitive regulator of MSC function.

Discussion

In the present study, we focused on the flux of acetyl-CoA from mitochondria to the nucleus, and we investigated how normoxia-associated changes in the flow of acetyl-CoA affect stem cell function. The data presented here suggest a model (Fig 6) whereby normoxia-driven changes in the chromatin structure lead to transcriptional alterations that are responsible for the decreased osteogenic potential of MSCs cultured under high oxygen conditions. We show that upon shifting cells from low to high oxygen, there is a switch in the subcellular localization of acetyl-CoA, which affects the epigenetic landscape. In particular, normoxic MSCs exhibit compartmentalized acetyl-CoA localization in mitochondria, as a result of impaired export to the cytosol, due to lower CiC activity. Strikingly, re-establishing the cytosolic acetyl-CoA pool, via acetate supplementation or through exogenous CiC overexpression, restores histone acetylation, and thus chromatin plasticity, and is sufficient to improve the impaired osteogenic capacity of normoxia-cultured MSCs. Hence, our findings highlight the fundamental role of CiC in the regulation of the metabolism–chromatin–osteogenesis axis in response to high oxygen levels.

The cellular environment plays a critical role in the regulation of stem cell fate decisions. Focusing on the role of oxygen tension on the metabolism–epigenome interplay with regards to stem cell differentiation, we report a negative impact of normoxia on the osteogenic potential of MSCs, which is in line with previous studies (Fehrer *et al*, 2007; Pattappa *et al*, 2013; Buravkova *et al*, 2014; Leijten *et al*, 2014). A recent report also highlighted that oxygen influences the metabolic state of MSCs and thus elicits changes in stem cell lineage commitment (Leijten *et al*, 2014), suggesting that the cellular energetic profile can be coupled to specific stem cell fate decisions. Consistent with this model, we identify that normoxia-cultured cells display reduced glycolysis (Fig EV2A–E), which is usually associated with stemness and enhanced differentiation capacity. However, beyond the normoxia-driven metabolic alterations, we demonstrate that osteogenesis is also impaired due to epigenetic remodelling which leads to chromatin compaction and histone hypo-acetylation on promoters and enhancers of osteogenic genes (Figs 2 and 3). This, in turn, results in lower expression of these genes and irreversible defects in osteogenesis (Fig 1D and E). Next to the impact on protein acetylation and lipogenesis, efficient export of citrate from mitochondria to the cytosol is required for the formation of cytosolic α-ketoglutarate (α-KG). Given that α-KG is a cofactor for histone demethylases, this branch of metabolism might also indirectly impact histone methylation levels and stem cell activity, as described previously (Morganti *et al*, 2020). In line with this idea, we did observe increased H3K9me2 levels in normoxic MSCs (Fig 3B). Whether changes in metabolism occur prior to the establishment of epigenetic alterations and the extent to which each of these two pathways contributes to the reduced MSCs function is definitely worth further investigation. However, establishing a causal relationship might be extremely challenging due to the tight connection between chromatin and metabolism.

© 2022 The Authors

It has been shown that CiC function can be influenced by lysine acetylation. In particular, K97 acetylation increases CiC activity as it stabilizes binding of its substrate citrate (Palmieri *et al*, 2015). However, several high-throughput studies have revealed many more potential acetylation (e.g. K255; Weinert *et al*, 2013) and phosphorylation (e.g. S156; Tsai *et al*, 2015) sites within CiC, suggesting that CiC activity can be strongly modulated by post-translational modifications (PTMs). Therefore, we speculate that although CiC levels are not impacted by high oxygen, CiC function might be compromised by specific normoxia-induced PTMs, leading to acetyl-CoA trapping within the mitochondria. Supporting our conclusions about reduced CiC activity in response to high oxygen levels, pharmacological or genetic modulation of CiC function, using the BTA inhibitor or exogenous CiC overexpression, respectively, directly influences histone acetylation and osteogenesis (Figs 5 and EV5). However, it is worth highlightening that normoxia represents a stress condition for bone MSCs, strongly affecting many cellular processes, including energy metabolism, and the oxygen-induced alterations in various biological functions could altogether lead to the phenotypes described here.

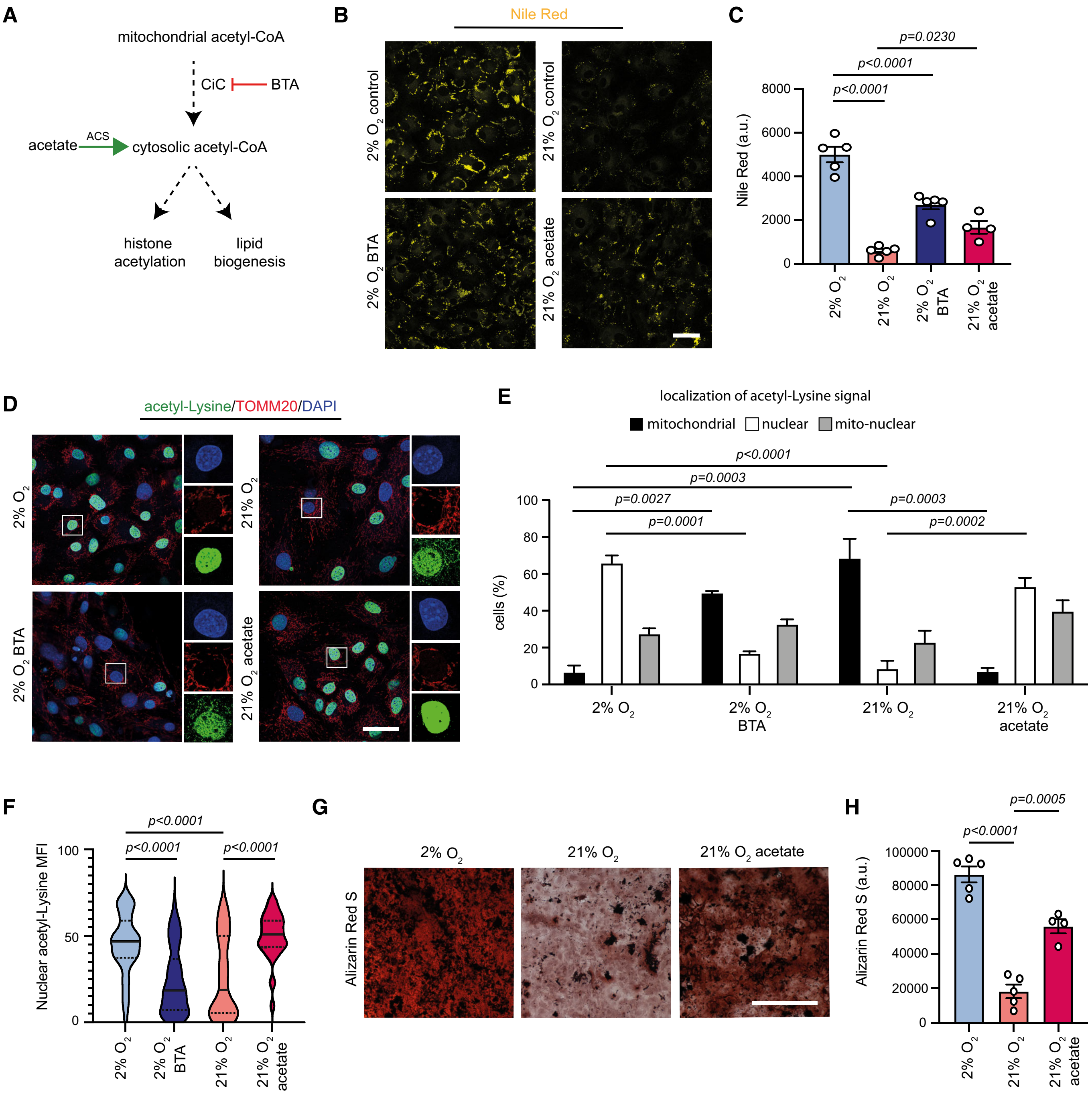

Figure 5.

© 2022 The Authors

◀ **Figure 5. Low CiC exporting activity in normoxia-cultured MSCs.**

A Schematic representation illustrating the different pathways generating cytosolic acetyl-CoA, its potential routes in the cytosol and the compounds used to inhibit (BTA) or bypass (acetate) CiC activity.

B, C Representative images (B) and quantification (C) of Nile Red staining of hypoxic control and BTA-treated cells and normoxic control and acetate-treated cells. Treatments were done for 2 days with 1 mM BTA or 5 mM acetate. $n = 3$ biologically independent experiments and the results of one representative experiment are shown. Scale bar, 25 µm. Results are shown as mean ± SEM and statistical significance was determined with ordinary one-way ANOVA, using Holm–Sidak's multiple-comparisons test.

D–F Representative images after immunostaining of hypoxic control and BTA-treated cells and normoxic control and acetate-treated cells against acetyl-lysine and TOMM20 (D), assessment of the acetyl-lysine signal localization, as described above (E), and quantification of nuclear acetyl-lysine MFI (F). Nuclei were stained with DAPI. Quantification of nuclear acetyl-lysine MFI from $n = 67$ hypoxic, $n = 71$ hypoxic_BTA, $n = 44$ normoxic and $n = 63$ normoxic_acetate individual cells from a representative experiment of three biologically independent experiments is shown in (F). Results are shown as mean ± SEM and statistical significance was determined with ordinary one-way ANOVA, using Holm–Sidak's multiple-comparisons test. The distribution of data points in (F) is shown as a violin plot, where the mean is indicated by a solid line and the quartiles are indicated with dashed lines. In magnified insets, the intensity of the acetyl-lysine signal was adjusted similarly to all samples, for visualization purposes. Scale bar, 75 µm.

G, H Representative images and quantification of Alizarin Red S staining of control hypoxic and normoxic cells and normoxic acetate-treated cells, 12 days after induction of osteogenesis. Merged results of $n = 5$ biologically independent experiments for hypoxic and normoxic cells and $n = 4$ biologically independent experiments for acetate-treated normoxic cells are shown in (H). Results are shown as mean ± SEM and statistical significance was determined with ordinary one-way ANOVA, using Holm–Sidak's multiple-comparisons test. Scale bar, 500 µm.

Source data are available online for this figure.

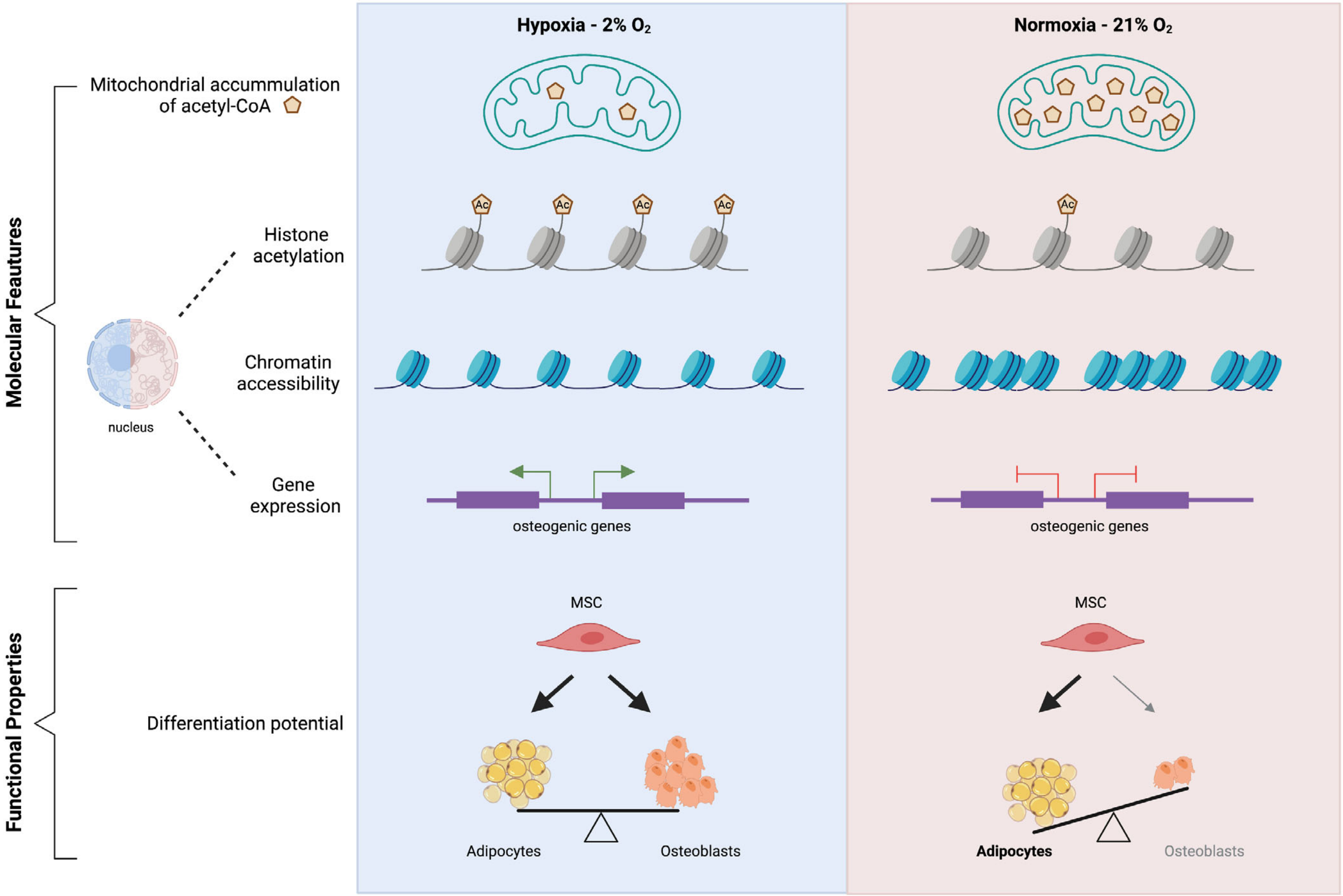

Figure 6. Model summarising the effects of normoxia on MSCs function.

Under hypoxic conditions, mitochondrial acetyl-CoA is efficiently exported to the cytosol and can be used to acetylate histones, leading to a plastic chromatin state, which ensures balanced adipogenic and osteogenic differentiation. Under high oxygen tension, acetyl-CoA remains trapped within mitochondria due to low CiC activity, leading to histone hypo-acetylation, chromatin compaction and a shift in the differentiation potential favouring adipogenesis at the expense of osteogenesis.

Strikingly, acetate supplementation of cells cultured under high oxygen is not only able to rescue histone acetylation levels but also restores the osteogenic differentiation potential—similar to the situation observed in ageing MSCs (Pouikli *et al*, 2021). Nonetheless, the impact of high oxygen on stem cell biology is much stronger than that of physiological ageing, as both chromatin accessibility and osteogenesis were more severely compromised under normoxic conditions. Notably, we performed these experiments after 7 days of exposure to normoxia; while this is a time point when metabolic alterations have been already established, it might not be sufficient for the reset of other cellular processes. Importantly, MSCs isolated from mouse and human tissues, which have adapted to atmospheric oxygen conditions, are proficient to differentiate into adipocytes, osteocytes and chondrocytes (Morikawa *et al*, 2009; Houlihan *et al*, 2012; Yang *et al*, 2018). However, it is clear from the data reported here and previously published work that the differentiation potential is strongly enhanced under low oxygen conditions (Basciano *et al*, 2011; Georgi *et al*, 2015).

Of note, murine and human MSCs exhibit different extent of sensitivity to oxygen levels. Under low oxygen conditions, MEFs proliferate over an extended period of time. By contrast, at atmospheric oxygen levels, MEFs undergo senescence after a few doublings, whereas some cells escape this cell cycle arrest and start proliferating, as an immortal cell line (Parrinello *et al*, 2003). This spontaneous immortalization correlates with the accumulation of DNA damage. Importantly, it is also known that mouse cells are more prone to acquire DNA lesions under normoxic conditions than cells derived from humans (Parrinello *et al*, 2003). Although we have not observed immortalization of MSCs, our findings indicate that an increase in oxygen concentration leads to phenotypes resembling physiological ageing. This raises the intriguing question of whether we investigate *de facto* aged cells in a lot of today's models that we consider physiological and identify oxygen concentration as a parameter that should be carefully considered when planning experiments using somatic stem cells as well as during stem cell therapies.

Materials and Methods

Reagents and Tools table

Reagent/resource	Reference or source	Identifier or catalog number
**Experimental models**		
C57BL/6N (M. musculus)	Charles River Germany, bred in house (Transgenic Core Facility of MPI for Biology of Ageing, Cologne, Germany)	Strain code: 027
Primary bone-mesenchymal stem cells (MSCs)	Isolated as described on methods	
Commercial bone-mesenchymal stem cells (MSCs)	Cyagen	MUBMX-01001
HEK293T cells	ATCC	
**Recombinant DNA**		
pLVX-Puro	Clontech	632164
pMD2.G	Addgene	12259
pspAX2	Addgene	12260
pLVX-puromycin-CiC	Generated for this paper; details on methods	
**Antibodies**		
Rabbit anti-acetyl-Lysine, Polyclonal (1:400)	Cell Signaling Technology	#9441S
Rabbit anti-FASN, Polyclonal (1:1,000)	Cell Signaling Technology	#3189S
Rabbit anti-AceCS1, Monoclonal (1:1,000)	Cell Signaling Technology	#3658T
Rabbit anti-FLAG, Monoclonal (1:500)	Cell Signaling Technology	#14793
Anti-rabbit IgG HRP-linked (1:2,000)	Cell Signaling Technology	#7074S
Anti-mouse IgG HRP-linked (1:2,000)	Cell Signaling Technology	#7076S
Mouse anti-histone H3, Monoclonal (1:200)	Cell Signaling Technology	#14269
Rabbit anti-ACC1, Polyclonal (1:1,000)	ProteinTech	#21923-1-AP
Rabbit anti-ACLY, Polyclonal (1:1,000)	ProteinTech	#15421-1-AP
Rabbit anti-Citrate carrier, Polyclonal (1:500)	ProteinTech	#15235-1-AP
Mouse anti-TOMM20, Monoclonal (1:200)	Sigma Aldrich	WH0009804M1

 © 2022 The Authors

Reagents and Tools table (continued)

Reagent/resource	Reference or source	Identifier or catalog number
Rabbit anti-H3K4me3, Polyclonal (1:100)	Active Motif	#39159
Rabbit anti-H3K27ac, Polyclonal (1:100)	Active Motif	#39133
Rabbit anti-histone H3ac, Polyclonal (1:500)	Active Motif	#39139
Mouse anti-SREBP1, Monoclonal (1:1,000)	Santa Cruz Biotechnology	sc-13551
Mouse anti-α-tubulin, Monoclonal (1:1,000)	Abcam	#7291
Anti-Rabbit IgG Alexa Fluor 488, Polyclonal (1:500)	ThermoFischer Scientific	#A21206
Anti-mouse IgG Alexa fluor 568, Polyclonal (1:500)	ThermoFischer Scientific	#A11031
Rat anti-CD140a-APC, Monoclonal (1:1,000)	eBioscience	#17140181
Rat anti-Sca-1-FITC, Monoclonal (1:1,000)	eBioscience	#11598185
Rat anti-Terr-119-PE, Monoclonal (1:1,000)	eBioscience	#12592182
Rat anti-CD45-PE, Monoclonal (1:1,000)	Life Technologies	#A16325
**Oligos used for RT-qPCR analysis (all from Sigma Aldrich)**	**Forward primer**	**Reversed primer**
Slc25a1 (encoding citrate carrier)	5′ GGAGAGGACTATTGTGCGGTCT 3′	5′ CCCGTGGAAAAATCCTCGGTAC 3′
Hk2	5′ GTGGCTAGAGCTCGGGATC 3′	5′ TTTCCAGTCGCCCAACATCT 3′
Pgk1	5′ GCGCCACCTTCTACTCCTCC 3′	5′ CTTCCATTTGTCACGTCCTG 3′
Pfk1	5′ ACCGAATCCTGAGTAGCAAG 3′	5′ GTAGCCTCACAGACTGGTTC 3′
Pgam1	5′ ATCAGCAAGGATCGCAGGTA 3′	5′ TTCATTCCAGAAGGGCAGTG 3′
β-actin	5′ CTGCGCTGGTCGTCG 3′	5′ CACGATGGAGGGGAATACAG 3′
**Chemicals, enzymes and other reagents**		
7-AAAD	Invitrogen	A1310
Alizarin Red S	Sigma Aldrich	A5533
Beta-glycerophosphate	Sigma Aldrich	G9422
BTA	Sigma Aldrich	B4201
Collagenase	Sigma Aldrich	C9407
Dexamethasone	Sigma Aldrich	D4902
Digitonin	Millipore	300410
IBMX	Sigma Aldrich	15879
Indomethacin	Sigma Aldrich	17378
Insulin	Sigma Aldrich	16634
ITS	Sigma Aldrich	I1884
L-ascorbic acid	Sigma Aldrich	A8960
L-proline	Sigma Aldrich	P5607
MitoTracker	ThermoFischer Scientific	M22426
Nile Red	Sigma Aldrich	N3013
Oil Red O	Sigma Aldrich	O0625
Polybrene	Santa Cruz	sc-134220
Puromycin	Gibco	A11138
Roti-Mount FluorCare mounting medium	ROTH	HP20.1
Sodium acetate	Invitrogen	AM9740
Sodium butyrate	Sigma Aldrich	156-54-7
Sodium pyruvate	Gibco	11360
UltraComp compensation beads	Invitrogen	1222242
**Software**		
BD FACS Diva	Version 8.0.1	
Bowtie2		

Reagents and Tools table (continued)

Reagent/resource	Reference or source	Identifier or catalog number
Compound Discoverer software	Version 3.2	
Ensembl	GRCm38.91	
FeatureCounts	Version 1.32.4	
Fiji	Version 2.1.0/1.53c	
FlowJo	Version10.7.2	
GraphPad Prism	Version 9.0.0	
Integrative Genomic Viewer (IGV)	Version 2.7.2	
Leica Application Suite X	3.5.7.23225	
LightCycler® 96	SW1.1 Inc	
MaxQuant	Version 1.5.3.17	
Primer3 web	Version 4.1.0	
R	Version 3.5.1	
Samtools	Version 1.9	
STAR	Version 2.6.1a	
TargetLynx Software (Waters)		
TraceFinder software	Version 5.0	
Wave	Version 2.4	
zUMIs	Version 2.2.1	
**Other**		
BCA Protein Assay Kit	ThermoFischer Scientific	23225
Direct-zol RNA MiniPrep - RNA extraction kit	Zymoresearch	R2050
MaximaTM H Minus reverse transcriptase kit	ThermoScientific	EP0751
NEBNext Ultra II RNA Library Prep Kit	Illumina	E7770
Nextera XT DNA Library Prep Kit	New England Biolabs	M2200S
Quick Ligation kit	Illumina	20034210
SeaHorse XF Glycolysis Stress Test kit	Agilent Technologies	103020
Seahorse XF Mitostress Test kit	Agilent Technologies	103015
Tagment DNA Enzyme and Buffer	Thermo Scientific	2034197
Acquity iClass UPLC connected to Xevo TQ-Se mass spectrometer	Waters	
BD FACSAria IIu and BD FACSAria Fusion instruments	BD Biosciences	
Curix60	Agfa	
EASY-nLC 1000 Liquid Chromatography system	Thermo Scientific	
EVOS FL AUTO	ThermoFisher Scientific	
Illumina HiSEq 4000	Illumina	
Illumina HiSEq 2500	Illumina	
JEM 2100 plus electron microscope	JEOL	
Laser-scanning confocal SP8-DLS and SP8-X	Leica	
Light Cycler 96	Roche Life Science	
Q Exactive Plus mass spectrometer	Illumina	
Trans-blot turbo blotting apparatus	Bio-Rad	
SeaHorse XF96 extracellular Flux Analyzer	Agilent Technologies	
Vanquish Horizon UHPLC coupled to Orbitrap Exploris 240 mass spectrometer	ThermoFischer Scientific	
ViCELL MetaFLEX instrument	Beckman Coulter	

© 2022 The Authors

Methods and Protocols

Mouse model, cell lines and experimental details

Cell lines

Commercial bone mesenchymal stem cells (Cyagen, MUBMX-01001) were cultured in normoxic or hypoxic conditions, as described below. HEK293T cells (ATCC) were cultured in normoxic conditions, as described below.

Mice

C57BL/6 N mice were bred and cared for in the mouse facility of the Max Planck Institute for Biology of Ageing. Mice were kept at a relative humidity of 50 ± 5%, a room temperature (RT) of 22 ± 2°C and a light/dark cycle of 12 h (6 a.m. to 6 p.m., with a 15-min twilight period).

For our experiments, we used exclusively wild-type male mice, 3–5 months old.

Endosteal MSCs isolation

To isolate MSCs from their endosteal niche, we followed a purification strategy based on a published protocol (Houlihan *et al*, 2012), and adapted the isolation strategy to acquire sufficient cells for our experiments. In brief, C57BL6/N mice were sacrificed by cervical dislocation. Skin and muscles around the hind limbs were removed, the legs were cut above the pelvic joints and then placed in ice-cold PBS, on ice. Tibias were then separated from femurs by dislocating the joints and the clean bones were placed back in ice-cold PBS. All the following steps were performed inside the hypoxia hood. Bones were crushed and cut into tiny pieces and bone chips were incubated at 37°C for 75 min, in MEM-Alpha medium containing 0.2% w/v collagenase (Sigma Aldrich), shaking at 200 rpm. To stop the collagenase reaction, sample tubes with the bone chips were placed on ice and washed with a control culture medium (CCM; MEM-Alpha medium supplemented with 10% FBS and 1% penicillin/streptomycin). We modified the published protocol by culturing the bone fragments together with the released cells; this allows the outgrowth of more MSCs from the bone *in vitro* before cell sorting by flow cytometry, increasing the cell yield we obtain. Bone chips were transferred into T25 flasks and were cultured under humidified conditions in hypoxia (2% O_2, 5% CO_2, 37°C). On day 3 of the cell culture, the medium was changed and on day 5, both the cells and the bone chips were passaged, using Trypsin/EDTA solution (Life Technologies). On day 8 of the cell culture, the bone chips were removed and on day 12, we performed cell sorting using flow cytometry.

Cell sorting by flow cytometry

To obtain a pure MSC population, we performed cell sorting by flow cytometry, using the 7-AAD viability dye and the following antibodies: Sca-1-FITC, CD140a-APC, CD45-PE and TER-119-PE (for details, please be referred to Reagents and Tools table). After harvesting, cells were washed with PBS and resuspended in Hank's Balanced Salt Solution (1× HBSS, 0.01 M HEPES, 2% FBS and 1% penicillin/streptomycin, all Life Technologies). After incubation with the antibodies for 45 min, cells were washed twice with HBSS+ and were filtered through 35 μm nylon mesh into 5 ml sample tubes. Cell sorting was performed using the BD FACSAria Fusion instrument (BD Biosciences) with the FACSDiva software (version 8.0.1). Cells were sorted at 4°C using a 100 μm nozzle and sheath pressure was set to 20 psi. The $CD45^-$/$TERR\text{-}119^-$/$CD140a^+$/$Sca\text{-}1^+$ population was sorted into CCM-containing Eppendorf tubes, at 4°C. Compensation was done using UltraComp compensation beads (#01222242, Invitrogen). Once sorted, MSCs were centrifuged at 300 *g* for 10 min at 4°C and resuspended in CCM. Cells were cultured under humidified conditions (5% CO_2, 37°C), in hypoxia (2% O_2) or normoxia (21% O_2), for 7 days.

qRT–PCR analysis

Cells were lysed with QIAzol (QIAGEN) and total RNA was extracted using the RNA extraction kit (Direct-zol RNA MiniPrep—Zymoresearch), following the manufacturer's protocol. This was followed by cDNA synthesis using MaximaTM H Minus cDNA synthesis master mix (Thermo Scientific), according to the manufacturer's instructions. Subsequent qRT–PCR was performed with 10 ng of cDNA, using SYBR-Green chemistry (Roche) on a Light Cycler 96 instrument (Roche). Data were analysed and further processed in Microsoft Excel and Prism9 software. Fold change in gene expression over control samples was calculated using the ΔΔCq method, where β-actin Cq values were used as an internal control. All reactions were run in three technical replicates and averaged. Experiments were performed three independent times and merged results are shown. Oligos were designed using Primer3 and Blast platforms.

Mitotracker staining

Cells were washed with PBS, harvested with Trypsin–EDTA and resuspended in the pre- warmed (37°C) staining solution containing the MitoTracker Deep Red FM probe (Thermo Fischer Scientific #M22426) in a final dilution 1:15,000 in assay medium (MEM-Alpha medium without FBS and phenol red). Cells were incubated with the staining solution for 30 min at 37°C. They were then washed twice with PBS, centrifuged at 500 *g* for 5 min and resuspended in the assay medium. After the addition of DAPI (Invitrogen) for dead-cell exclusion right before measurement, cells were analysed by flow cytometry (BD FACSCANTO II cytometer, BD Biosciences). Data were collected using the FACS-Diva software (version 8.0.1) and analysed using the FlowJo software (version 10.7.2).

Seahorse analyses of cellular energetic profile

The SeaHorse XF96 extracellular Flux Analyzer (Agilent Technologies) was used to determine oxygen consumption rate (OCR) in hypoxia- and normoxia-cultured MSCs. 2×10^4 cells were seeded in 96-well SeaHorse plates, after coating them with 10% gelatin and 90% poly-L-lysine solution for 1 h. Cells were incubated overnight with CCM in a humidified incubator. On the day of the experiment, cells were washed twice with assay medium (XFDMEM, 10 mM Glucose, 1 mM Pyruvate and 2 mM L-glutamine) and incubated for 1 h prior to loading into the XF Analyzer, in a non-CO_2-containing incubator. Following measurements of resting respiration, cells were injected subsequently with 20 μM oligomycin, 5 μM FCCP and 5 μM rotenone/antimycin (all drugs were from Agilent Technologies). Each measurement was taken over a 2-min interval followed by 2 min mixing and 2 min incubation. Three measurements were taken for the resting OCR: after oligomycin treatment, FCCP and rotenone/antimycin A treatment.

To determine the extracellular acidification rate (ECAR), 3×10^4 cells were seeded in 96-well SeaHorse plates, as described above.

© 2022 The Authors

On the day of the experiment, cells were washed twice with the assay medium (XF-DMEM, 2 mM L-glutamine) and incubated for 1 h in a non-CO_2-containing incubator, before loading into the XF analyser. The glycolytic activity was measured after injection with 10 μM glucose, 1 μM oligomycin and 50 μM 2-DG. Each measurement was taken over a 2 min interval followed by 2 min mixing and 2 min incubation.

In both assays, raw values were normalized to protein concentration, measured using Bradford kit, and were plotted using Wave 2.4 (version 2.4) and the Prism9 software programmes.

Differentiation assays

Differentiation to adipocytes For adipogenesis experiments, 2×10^3 cells were seeded in 96-well plates. Adipogenic differentiation was induced once cells reached confluency, by culturing them in CCM or adipogenic medium (AM; CCM supplemented with 1 μM dexamethasone, 1 μM IBMX, 10 μg/ml insulin and 100 μM indomethacin; all bought from Sigma Aldrich), for 8–10 days. Adipocytes were detected by Oil-Red-O (Sigma Aldrich) staining. Cells were washed with PBS and fixed in 3.7% formaldehyde (Roth) for 30 min, at RT. After fixation, cells were washed twice with ddH_2O and once with 60% isopropanol (Roth), with every washing step lasting 5 min. Cells were then stained with Oil-Red-O staining solution for 15 min at RT. After staining, cells were washed four times with ddH_2O and images were acquired with a bright-field microscope, using the 20× objective.

Differentiation to osteoblasts For osteogenesis experiments, 2×10^3 cells were seeded in 96-well plates. Osteogenesis was induced once cells reached confluency, by culturing them in CMM or osteogenic medium (OM; CCM supplemented with 100 nM dexamethasone, 10 mM betaglycerophosphate and 100 μM ascorbic acid; all bought from Sigma Aldrich), for 11 days. For sodium acetate treatment of normoxia-cultured cells, 5 mM of sodium acetate was added to the media 3 days prior to induction of differentiation and was then removed. To observe whether osteogenic defects under normoxic conditions are reversible, cells were cultured in normoxia for 7 days, were then shifted to hypoxia and osteogenesis was induced after 4 days, by culturing cells with OM.

Osteoblasts were detected by Alizarin Red S (Sigma Aldrich) staining performed 12 days after induction of osteogenesis. Cells were washed once with PBS and fixed in 3.7% formaldehyde for at least 30 min at RT. Fixation was followed by washing of cells with ddH_2O. Cells were then incubated with the Alizarin Red S staining solution (2% w/v Alizarin Red S in ddH_2O) for 45 min, protected from light. After staining, cells were washed with ddH_2O and images were acquired with a bright-field microscope, using the 20× objective.

Immunofluorescence experiments

For immunofluorescence experiments, 2×10^3 cells were seeded in 96-well plates with glass bottom (Greiner) and treated as indicated in each experiment. After treatments, cells were fixed for 15 min at 37°C with 3.7% v/v formaldehyde in CCM. Samples were washed with PBS twice, permeabilized with 0.1% TritonX-100 (Roth) in PBS for 10–15 min and blocked with 5% BSA (Roth) in PBS for 10 min. Samples were then incubated with the indicated primary antibodies diluted in 3% BSA-PBS, overnight at 4°C (for details, please be referred to Reagents and Tools table). Following this, samples were washed three times with PBS, with each washing step lasting 10 min. Samples were then incubated with the appropriate secondary fluorescent antibodies diluted 1:500 in 5% BSA-PBS for 45 min, protected from light. After three 10-min washing steps with PBSEN, cells were mounted using the Roti-Mount FluorCare mounting medium (HP20.1, ROTH), containing DAPI. Images were acquired using 40× and 63× objective lenses on SP8-X and SP8-DLS Leica confocal microscopes.

Nile Red staining

One millimetre of the Nile Red dye (Sigma Aldrich) was added to the cells, diluted in HBSS+ buffer. Cells were incubated with the dye for 15 min at 37°C. They were then washed three times with HBSS+ buffer and live cell imaging was performed using the SP8-DLS Leica confocal microscope, with a 40× objective lens.

Electron microscopy

Samples were fixed in fixation buffer (2% glutaraldehyde, 2.5% sucrose, 3 mM $CaCl_2$ and 100 mM HEPES pH 7.4) for 30 min at RT and 30 min at 4°C. They were then washed with 0.1 M sodium cacodylate buffer (1% osmium, 1.25% sucrose and 10 mg/ml K3 [Fe (CN)6] in 0.1 M sodium cacodylate), incubated for 1 h on ice OsO_4 and washed again with 0.1 M sodium cacodylate buffer. After washes with EtOH, samples were incubated with EPON and embedded. Sections (70 nm) were cut using the Leica Ultracut and put on negatively stained grids with 1.5% uranylacetate in water, at 37°C in the dark. Images were acquired using a JEM 2100 plus microscope (JEOL).

Western blot experiments

For all western blot experiments, cells were harvested and lysed with RIPA lysis buffer (150 mM NaCl, 1% TritonX-100, 0.5% sodium deoxycholate, 0.1% SDS and 50 mM Tris pH 8.0) supplemented with 5 mM sodium butyrate and 1× Protease Inhibitor Cocktail (Thermo Scientific). For efficient cell lysis, cells were incubated with RIPA lysis buffer at 4°C, for 30 min rotating, and then centrifuged for 10 min at 6,500 *g*. Protein concentration was determined using BCA protein assay kit (Thermo Fischer Scientific). Twenty to fifty microgram of total protein was loaded into each well and SDS–PAGE electrophoresis was performed at 150 V for 45 min. This was followed by transfer to a nitrocellulose membrane, using the Trans-Blot Turbo blotting apparatus and reagents, all provided by Bio-Rad. Protein transfer was confirmed by Ponceau S staining (Sigma Aldrich) for 1–2 min. The membranes were then blocked using 5% non-fat dry milk in Tris-buffered saline-0.1% Tween20 (TBS-T) for 1 h at RT. Membranes were incubated with the indicated primary antibodies (for details, please be referred to Reagents and Tools table), diluted in 5% Milk in TBS-T, at 4°C overnight, washed three times with TBS-T and incubated with the appropriate horseradish peroxidase (HRP)-conjugated secondary antibodies diluted 1:5,000 in 5% BSA in TBS-T, for 1 h at RT. After three 10-min washing steps in TBS-T, the desired proteins were visualized by providing fresh HRP substrate solution (Luminol Enhancer Solution/Peroxide Solution—Promega) and exposure of membranes for specific time periods to photographic film, using the Curix60 Instrument (Agfa).

© 2022 The Authors

Plasmid construction, generation of lentivirus and MSCs transduction
Construct pLVX-Puro-CiC was generated as described before (Pouikli *et al*, 2021) by cloning the CiC cDNA sequence fused to C-terminal FLAG epitope (synthesized by Geneart) into a pLVX-Puro vector (Takarabio) between the *Xho*I and *Xba*I restriction sites. Lentivirus was generated by co-transfection of HEK293 T cells, cultured in control DMEM medium (CDM; DMEM-GlutaMAX, 10% FBS and 1× penicillin/streptomycin) with the pLVX-Puro vector or the pLVX-Puro-Slc25a1-FLAG, pMD2.G and psPAX2 vectors (the pMD2.G and psPAX2 were a gift from D. Trono; Addgene plasmid catalogue nos. 12259 and 12260, respectively). Specifically, 2.8 million HEK cells were seeded in a 10 cm plate. The next day, a transfection mix of 500 ml 2 × HBS, 62 ml $CaCl_2$ 2 M, 10 mg pLVX, 5.2 mg pMD2.G and 5.2 mg psPAX2 was incubated for 30 min at RT and subsequently added to the cells. After 16 h, the medium was changed to 6 ml fresh CDM. After 72 h, the supernatant was collected, spun at 500 g for 5 min and filtered through a 0.45 μm filter.

A 1:1 virus:medium ratio along with 4 μg/ml polybrene was used to transduce MSCs. The medium was changed after 18 h. Puromycin (2 μg/ml) was added after 72 h to positively select transduced cells.

Measurement of glucose, lactate and pH
Measurement of glucose, lactate and pH in the media of MSCs was performed using the ViCELL MetaFLEX instrument (Beckman Coulter) according to manufacturer's instructions. All measurements were done in triplicates and averaged.

Targeted LC/MS analysis of acetyl-CoA
Metabolite extraction from each sample was performed using a mixture of 40:40:20 (v:v:v) of pre-chilled (−20°C) acetonitrile: methanol:water (OptimaTM LC/MS grade, Thermo Fisher Scientific), and protein concentration was used for normalization (BCA Protein Assay Kit, Thermo Fisher Scientific). For analysis of acetyl-CoA, the extracted metabolites were resuspended in UPLC-grade acetonitrile:water (80:20) (v:v) (OptimaTM LC–MS-grade, Thermo Fisher Scientific). The samples were analysed on an Acquity iClass UPLC (Waters), using a SeQuant ZIC-HILIC 5 μm polymer 100 × 2.1 mm column (Merck) connected to a Xevo TQ-S (Waters) triple quadrupole mass spectrometer. The resuspended metabolite sample extract was injected onto the column and separated using a flow rate of 500 μl/min of buffer A (10 mM ammonium acetate and 0.1% acetic acid) and buffer B (acetonitrile) using the following gradient: 0–0.5 min 20% A; 0.5–1.4 min 20–35% A; and 1.4–2.5 min 35–65% A. After 2.5 min, the system was set back to 20% A and re-equilibrated for 2.5 min.

The eluted metabolites were detected in positive ion mode using ESI MRM (multireaction monitoring) applying the following settings: capillary voltage 1.5 kV, desolvation temperature 550°C, desolvation gas flow rate 800 l/h and collision cell gas flow 0.15 ml/min. The following MRM transitions were used for relative compound quantification of acetyl-CoA *m/z* precursor mass (M+H+) 810, fragment mass (M+H+) *m/z* 303 using a cone voltage of 98 V and collision energy of 28 V. For each compound, two further fragments were monitored as qualitative controls for compound identity. Data analysis and peak integration were performed using the TargetLynx Software (Waters).

LC/MS metabolomic analyses after cellular fractionation or metabolic labelling

Cellular fractionation To isolate mitochondrial and cytosolic cellular fractions, we followed a previously published rapid subcellular fractionation method (Lee *et al*, 2019). Briefly, commercially available murine MSCs were cultured in T175 flasks under normoxic or hypoxic conditions for 7 days in CCM. On the day of the experiment, CMM was removed, cells were washed twice with ice-cold PBS and scraped from the flasks with 2 ml PBS. A ratio of 1:4 of cells was transferred to a tube and served as the whole-cell lysate (WCL). After a brief centrifugation step (10,000 *g*, 3 min, 4°C), the supernatant was removed and 300 μl of metabolite extraction solution (50% MetOH:30% acetonitrile:20% ultrapure water) was added to samples. Samples were incubated with the extraction buffer for 20 min in a dry-ice MetOH water bath and were then subjected to metabolite extraction. The rest 3:4 of cells was subjected to digitonin-based cellular fractionation. More precisely, after a brief centrifugation step (10,000 *g*, 10 s, 4°C), the supernatant was removed and the pellet was resuspended in 1 ml of ice-cold digitonin-PBS buffer (1 mg/ml). Following quick centrifugation (10,000 *g*, 10 s, 4°C), supernatant and pellet were collected as the cytosolic and mitochondrial fractions respectively. Four millilitre of 50% MetOH:30% acetonitrile was added in the cytosolic fractions to extract metabolites. Samples were incubated for 20 min in a dry-ice MetOH water bath and were then subjected to metabolite extraction. The mitochondrial fraction was resuspended in 100 μl metabolite extraction solution and incubated for 20 min in a dry-ice MetOH water bath and was then subjected to metabolite extraction.

Metabolite extraction The cell extract suspension was incubated for 15 min, at 4°C, shaking in a thermomixer at maximum speed. Samples were then centrifuged for 20 min at 4°C, at 13,000 rpm. The top 80% of the supernatant was collected and subjected to LC/MS analysis.

LC/MS analysis of extracted metabolites Chromatographic separation of metabolites was achieved using a Millipore Sequant ZIC-pHILIC analytical column (5 μm, 2.1 × 150 mm) equipped with a 2.1 × 20 mm guard column (both 5 mm particle size) with a binary solvent system. Solvent A was 20 mM ammonium carbonate and 0.05% ammonium hydroxide; Solvent B was acetonitrile. The column oven and autosampler tray were held at 40 and 4°C respectively. The chromatographic gradient was run at a flow rate of 0.200 ml/min as follows: 0–2 min: 80% B; 2–17 min: linear gradient from 80 to 20% B; 17–17.1 min: linear gradient from 20 to 80% B; and 17.1–23 min: hold at 80% B. Samples were randomized and the injection volume was 5 μl. A pooled quality control (QC) sample was generated from an equal mixture of all individual samples and analysed interspersed at regular intervals.

Metabolites were measured with Vanquish Horizon UHPLC coupled to an Orbitrap Exploris 240 mass spectrometer (both Thermo Fisher Scientific) via a heated electrospray ionization source. The spray voltages were set to +3.5 kV/−2.8 kV, RF lens value at 70, the heated capillary held at 320°C and the auxiliary gas heater held at 280°C. The flow rate for sheath gas, aux gas and sweep gas were set to 40, 15 and 0 respectively. For MS1 scans, mass range was set to *m/z* = 70–900, AGC target was set to standard and maximum

© 2022 The Authors

injection time (IT) was set to auto. Data acquisition for experimental samples used full-scan mode with polarity switching at an Orbitrap resolution of 120,000. Data acquisition for untargeted metabolite identification was performed using the AcquireX Deep Scan workflow, an iterative data-dependent acquisition (DDA) strategy using multiple injections of the pooled sample. DDA full-scan ddMS2 method for AcquireX workflow used the following parameters: full-scan resolution was set to 60,000, fragmentation resolution to 30,000 and fragmentation intensity threshold to 5.0e3. Dynamic exclusion was enabled after one time and the exclusion duration was 10 s. Mass tolerance was set to 5 ppm. The isolation window was set to 1.2 *m/z*. Normalized HCD collision energies were set to stepped mode with values at 30, 50 and 150. The fragmentation scan range was set to auto, AGC target at standard and max IT at auto. Mild trapping was enabled.

Metabolite identification was performed in the Compound Discoverer software (v 3.2, Thermo Fisher Scientific). Metabolite identities were confirmed using the following parameters: (i) precursor ion *m/z* was matched within 5 ppm of theoretical mass predicted by the chemical formula; (ii) fragment ions were matched within 5 pm to an in-house spectral library of authentic compound standards analysed with the same ddMS2 method with the best match score of over 70; and (iii) the retention time of metabolites was within 5% of the retention time of a purified standard run with the same chromatographic method. Chromatogram review and peak area integration were performed using the Tracefinder software (v 5.0, Thermo Fisher Scientific) and the peak area for each detected metabolite was normalized against the total ion count (TIC) of that sample to correct any variations introduced from the sample handling to instrument analysis. The normalized areas were used as variables for further statistical data analysis.

SILAC-MS analysis

SILAC labelling Commercially available murine MSCs were used as a standard for both hypoxia and normoxia cultures. To label commercial MSCs, cells were grown in DMEM HG (without lysine and arginine, Thermo Scientific, # 88420), supplemented with 10% dialysed FBS (Thermo Scientific, # 26400044), heavy lysine (146 mg/l; Sigma, # 608041) and arginine (28 mg/l; Sigma # 608033), for a total of six passages. The total incorporation of heavy amino acids was determined by mass spectrometry to be > 97%. Primary MSCs were cultured under hypoxic or normoxic conditions for 7 days after cell sorting, using the same medium, but with light lysine/arginine (Sigma, # L8662 and # A8094). Cells were subsequently mixed 1:1 and histones were isolated as described below.

Enrichment of histones A total of 10^7 cells in each replicate were harvested and spun down. Pellets were resuspended in 0.1 M H_2SO_4 and nutated for 2 h at 4°C. The solution was homogenized and subsequently centrifuged for 20 min at 3,500 rpm, at 4°C. Supernatant was neutralized with 1 M Tris–HCl pH 8.0 and the following reagents were added: 0.5 M NaCl, 2 mM EDTA, 0.25 mM PMSF and 1 mM DTT. Solution was added to a 2 ml SP sepharose, washed with wash buffer (50 mM Tris pH 8, 0.6 M NaCl, 2 mM EDTA, 1 mM DTT and 0.25 mM PMSF) and eluted in elution buffer (50 mM Tris pH 8, 0.2 M NaCl, 2 mM EDTA, 1 mM DTT and 0.25 mM PMSF). Proteins were precipitated with 4% perchloric acid overnight at 4°C and then spun at 14,000 rpm for 45 min at 4°C. Pellets were washed two times with 4% perchloric acid, two times with 0.2% HCl in acetone and two times with acetone. Pellets were dried and resuspended in ddH2O with 0.25 M PMSF.

Protein digestion Proteins were digested using partial FASP as previously described (Leidecker *et al*, 2016). Briefly, proteins were resuspended in 8 M urea in 0.1 M Tris–HCl pH 8.0, 10 mM tris (2carboxyethyl) phosphine (TCEP), 20 mM chloroacetamide and transferred to 10 kDa cut-off Vivacon® 500 flat filters. Samples were centrifuged at 14,000 *g* at 20°C for 20 min, followed by three washes with 50 mM ammonium bicarbonate (ABC). For partial FASP digestion, 1:2,000 trypsin gold-to-protein ratio was used for 20 min at 20°C. The digestion was stopped by the addition of formic acid to reduce the pH below 3. Peptides were collected by centrifugation at 14,000 *g* at 4°C for 10 min. Next, 50 mM ABC was added to the filter, and peptides were collected by centrifugation at 14,000 *g* at 4°C. This elution step was repeated. The retentate containing undigested proteins was further digested in 50 mM ABC overnight at 37°C, with 1:50 trypsin to protein ratio. Peptides were collected by centrifugation at 14,000 *g* at 4°C for 10 min, followed by a further elution with 50 mM ABC. Peptides were then desalted on either C18 cartridges (3 M Empore) or using in-house manufactured StageTips (Rappsilber *et al*, 2002), depending on the peptide amounts. Eluted peptides were dried down in Speedvac concentrator and resuspended in 0.1% FA prior to LC–MS/MS analysis.

LC–MS/MS analysis Liquid chromatography was performed on an EASY-nLC 1000 Liquid Chromatography System (Thermo Scientific) coupled to Q Exactive Plus mass spectrometer (Thermo Scientific) via modified NanoFlex sources (Thermo Scientific). Peptides were loaded onto 250 mm × 75 μm PicoFrit (C18 2 μm medium) analytical columns (New Objective) at a maximum pressure of 800 bar. Solutions A and B for the UPLCs were 0.1% formic acid in water and acetonitrile respectively. Samples were loaded in 0.1% formic acid in water to maximize the retention of highly hydrophilic peptides. Gradients varied slightly in length (90–150 min) and mixture, and may be extracted from the respective raw files. In general, they incorporated a linear gradient from very low or zero %B to 20 or 30% for 65–100 min, followed by a steeper phase and a wash. This length of gradient was maintained despite the relative simplicity of the protein mixture to improve the resolution and identification of as many modified peptide forms as possible, including those of low abundance. Full-scan MS spectra were acquired from over an *m/z* range 300–1,800 at 70,000 resolution, AGC targets were set to 3,000,000 ions and the maximum injection time was 100 ms. MS2 acquisition varied slightly in resolution, AGC target and maximum injection time, and may be extracted from the respective raw files. In general, up to five data-dependent HCD fragmentation, MS2 spectra were acquired at a resolution up to 70,000. AGC target for MS2 was set up to 1,000,000 ions. To reach this target, long MS2 injection times were allowed (up to 500 ms). Unassigned, singly charged or > +8-charged ions were rejected and the dynamic exclusion option was enabled (duration: up to 40 s).

Data analysis Raw files were analysed with MaxQuant proteomics suite of algorithms (version 1.5.3.17) (Cox & Mann, 2008), integrated with the search engine Andromeda (Cox *et al*, 2011). The

 © 2022 The Authors

data were searched against a mice proteome database (downloaded 09.10.2015 from UniProt) with the following parameters: the maximum allowed mass deviation was set to 4.5 ppm for precursor ions and 20 ppm for fragment ions; the minimum peptide length was set to 6 amino acids; the maximum number of missed cleavages was set to 5 with the maximum charge state 6; and multiplicity was set to 2 with Lys8/Arg10 as the Heavy Label and max. Labelled AAs were set to 7. Variable modifications included acetylation (protein N-term and K), methylation (KR), di-methylation (KR), tri-methylation (K) and phosphorylation (STY). FTMS top peaks per 100 Da were set to 20.

RNA-seq

Total RNA was isolated using the RNA extraction kit (Direct-zol RNA MiniPrep - Zymoresearch), following the manufacturer's protocol. Once the RNA quality and integrity were verified, RNA was submitted to library production at the Genomic Core Facility of the Max Planck Institute for Plant Breeding, Cologne, using the NEBNext Ultra II RNA Library Prep Kit. Libraries were sequenced as single-end 150 bp reads on Illumina HiSeq 4000. The sequenced reads of RNA-seq dataset were processed using zUMIs (version 2.2.1) (Parekh *et al*, 2018) with STAR (version 2.6.1a) (Dobin *et al*, 2013), samtools (version 1.9) (Li *et al*, 2009) and featureCounts from Rsubread (version 1.32.4) (Liao *et al*, 2014). The reads were mapped to the mouse genome (mm10) with the ensembl annotation version GRCm38.91. The generated count matrix was further analysed using R (version 3.5.1). Firstly, genes were filtered using "filterByExpr" function of edgeR (Robinson *et al*, 2010), with the minimum count of 5. The differential gene expression analysis between normoxic and hypoxic cells was carried out using limma trend (Ritchie *et al*, 2015) approach at the adjusted *P*-value of 0.05. Obtained sets of genes were further analysed through gene ontology (GO) enrichment analysis.

ATAC-seq

ATAC-seq was performed on 5×10^4 cells per sample, as described previously (Buenrostro *et al*, 2013). DNA concentration of the libraries was measured using Qubit, and library quality was assessed by running samples on the TapeStation. Libraries were sequenced on an Illumina HiSeq 2500. The fastq files of sequenced reads were mapped to the mouse genome (mm10) using local alignment with bowtie2 (Langmead & Salzberg, 2012) with parameters -x mm10 and -X 2000. The resulting BAM files were sorted, indexed using samtools (version 1.3.1) and duplicates were removed using MarkDuplicates of Picard Tools. The peaks were called using chromstaR (preprint: Taudt *et al*, 2016) R package in differential mode between normoxic and hypoxic cells with the bin size of 300, step size of 100 and 15 as minimum mapping quality threshold. The per-sample peak RPKM table was pulled out from the chromstaR model and differential accessibility analysis between normoxic and hypoxic cells was performed using edgeR (Robinson *et al*, 2010). The normalising factors were calculated using "RLE" method within "calcNormFactors," tagwise dispersion trend was estimated using the default parameters in "estimateDisp" function and a generalized linear model was then fit on the data using "glmQLFit" function in robust mode. The peaks called by chromstaR were then used in nucleoATAC (Schep *et al*, 2015) for nucleosome positioning. For visualization purposes, the replicates were merged using "samtools merge" and the bigwig files were generated using "baCoverage --normalizeUsing RPGC."

ChIP-seq

ChIP-seq was performed as described previously (Tessarz *et al*, 2014), using 10^5 MSCs per reaction. The fastq reads were mapped to the mouse genome (mm10) using bowtie2 (Langmead & Salzberg, 2012), and duplicates were then removed using MarkDuplicates program of Picard Tools. For mapping spike-in fragments to yeast, the --no-overlap --no-dovetail options were set and mapped to a repeat-masked version of the yeast genome (R64) to avoid cross-mapping of the mouse genome to that of the yeast genome. The peaks were then called using chromstaR (preprint: Taudt *et al*, 2016) package in differential mode between hypoxic and normoxic cells for H3K27ac and H3K4me3 with the bin size of 1,000, step size of 500 and 15 as minimum mapping quality threshold. The differential analysis between hypoxic and normoxic cells for H3K27ac mark was performed using edgeR (Langmead & Salzberg, 2012) as described under the ATAC-seq analysis section. For visualization purposes, the replicates were merged using "samtools merge" and the bigwig files were generated using "bamCoverage --normalizeUsing RPGC."

Image acquisition and processing

Bright-field images were acquired using the Evos FL Auto 2 microscope. Quantification of Alizarin Red S staining in Figs 1E and 5H was done in Fiji after applying thresholding criteria (0–32) similarly to all images and a fraction of the stained area (%area) is plotted. Immunofluorescent images were acquired using confocal SP8-X and SP8-DLS microscopes (Leica). All immunofluorescent images were processed identically in ImageJ; in particular, images are shown after background subtraction (rolling ball radius: 50) and noise despeckle. Quantification of Nile Red staining in Figs 5C and EV3B was done in Fiji after applying thresholding criteria (105–255) similarly to all images and a fraction of the stained area (%area) is plotted.

Quantification and statistical analysis

Except for epigenetic analyses, all other graphs were generated in GraphPad Prism9. For all bar graphs and violin plots, results are shown as mean ± SEM. For quantification of images where more than 10 cells were taken into account, the distribution of data points is shown as violin plots, where the mean is indicated by a solid line and quartiles are indicated with dotted lines. *P*-values for box plots of sequencing data were determined using a two-sided Wilcoxon test. Statistical significance was determined using a two-sided unpaired *t*-test when comparing two independent samples (e.g. normoxic vs. hypoxia). For multiple comparisons, ordinary one-way ANOVA was done using Holm–Sidak's multiple-comparisons test. The exact *P*-values are indicated on each plot. We performed GO enrichment analysis using Metascape, which calculates *P*-values on the basis of the accumulative hypergeometric distribution.

Data availability

The mass spectrometry proteomics raw data (MaxQuant output files) can be found in Dataset EV1. RNA-, ChIP- and ATAC-seq data are

© 2022 The Authors

available at GEO, accession numbers: GSE143580 (https://www.ncbi.nlm.nih.gov/geo/query/acc.cgi?acc=GSE143580), GSE200070 (https://www.ncbi.nlm.nih.gov/geo/query/acc.cgi?acc=GSE200070) and GSE200071 (https://www.ncbi.nlm.nih.gov/geo/query/acc.cgi?acc=GSE200071). Metabolomics data are available at Metabolomics Workbench (Sud *et al*, 2016), accession number: M8RD8W (http://doi.org/10.21228/M8RD8W).

Expanded View for this article is available online.

Acknowledgements

The authors would like to thank members of the Tessarz lab for the fruitful discussion on the impact of oxygen on cell physiology. Flow cytometry and fluorescent microscopy were performed at the FACS & Imaging Core Facility of the MPI for Biology of Ageing, Cologne. EM was performed at the Imaging facility of CECAD, University of Cologne. Sequencing was carried out at the Sequencing Core Facility of the MPI for Molecular Genetics, Berlin. This work was funded by the Max Planck Society (to IM and PT), the German Excellence Strategy (CECAD; EXC 2030-390661388, to IM and PT), a CRUK Programme Foundation award (C51061/A27453, to CF), an ERC Consolidator Grant (ONCOFUM, ERC819920, to CF) and by the Alexander von Humboldt Foundation in the framework of the Alexander von Humboldt Professorship endowed by the Federal Ministry of Education and Research (to CF). IM was supported by the EMBO Young Investigator Program, AP by an Onassis foundation graduate fellowship, MM by an Alexander von Humbodlt postdoctoral fellowship and JJB by a Marie Sklodowska-Curie grant. Models were drawn using Biorender. Open Access funding enabled and organized by Projekt DEAL.

Author contributions

Andromachi Pouikli: Conceptualization; investigation; methodology; writing – original draft; writing – review and editing. **Monika Maleszewska:** Conceptualization; investigation; methodology; writing – review and editing. **Swati Parekh:** Data curation; formal analysis; writing – review and editing. **Ming Yang:** Formal analysis; investigation; methodology; writing – review and editing. **Chrysa Nikopoulou:** Investigation; writing – review and editing. **Juan Jose Bonfiglio:** Formal analysis; investigation; methodology; writing – review and editing. **Constantine Mylonas:** Formal analysis; writing – review and editing. **Tonantzi Sandoval:** Investigation; writing – review and editing. **Anna-Lena Schumacher:** Investigation; writing – review and editing. **Yvonne Hinze:** Investigation; methodology; writing – review and editing. **Ivan Matic:** Supervision; funding acquisition; methodology; writing – review and editing. **Christian Frezza:** Supervision; funding acquisition; methodology; writing – review and editing. **Peter Tessarz:** Conceptualization; supervision; funding acquisition; writing – original draft; project administration; writing – review and editing.

Disclosure and competing interests statement

The authors declare that they have no conflict of interest.

References

Basciano L, Nemos C, Foliguet B, de Isla N, de Carvalho M, Tran N, Dalloul A (2011) Long term culture of mesenchymal stem cells in hypoxia promotes a genetic program maintaining their undifferentiated and multipotent status. *BMC Cell Biol* 12: 12

Batie M, Frost J, Shakir D, Rocha S (2022) Regulation of chromatin accessibility by hypoxia and HIF. *Biochem J* 479: 767–786

Buenrostro JD, Giresi PG, Zaba LC, Chang HY, Greenleaf WJ (2013) Transposition of native chromatin for fast and sensitive epigenomic profiling of open chromatin, DNA-binding proteins and nucleosome position. *Nat Methods* 10: 1213–1218

Buravkova LB, Andreeva ER, Gogvadze V, Zhivotovsky B (2014) Mesenchymal stem cells and hypoxia: where are we? *Mitochondrion* 19 Pt A: 105–112

Cox J, Mann M (2008) MaxQuant enables high peptide identification rates, individualized p.p.b.-range mass accuracies and proteome-wide protein quantification. *Nat Biotechnol* 26: 1367–1372

Cox J, Neuhauser N, Michalski A, Scheltema RA, Olsen JV, Mann M (2011) Andromeda: a peptide search engine integrated into the MaxQuant environment. *J Proteome Res* 10: 1794–1805

Dai Z, Ramesh V, Locasale JW (2020) The evolving metabolic landscape of chromatin biology and epigenetics. *Nat Rev Genet* 21: 737–753

Dobin A, Davis CA, Schlesinger F, Drenkow J, Zaleski C, Jha S, Batut P, Chaisson M, Gingeras TR (2013) STAR: ultrafast universal RNA-seq aligner. *Bioinformatics* 29: 15–21

Etchegaray J-P, Mostoslavsky R (2016) Interplay between metabolism and epigenetics: a nuclear adaptation to environmental changes. *Mol Cell* 62: 695–711

Fehrer C, Brunauer R, Laschober G, Unterluggauer H, Reitinger S, Kloss F, Gülly C, Gassner R, Lepperdinger G (2007) Reduced oxygen tension attenuates differentiation capacity of human mesenchymal stem cells and prolongs their lifespan. *Aging Cell* 6: 745–757

Georgi N, Cillero-Pastor B, Eijkel GB, Periyasamy PC, Kiss A, van Blitterswijk C, Post JN, Heeren RMA, Karperien M (2015) Differentiation of mesenchymal stem cells under hypoxia and normoxia: lipid profiles revealed by time-of-flight secondary ion mass spectrometry and multivariate analysis. *Anal Chem* 87: 3981–3988

Gomez-Salazar M, Gonzalez-Galofre ZN, Casamitjana J, Crisan M, James AW, Péault B (2020) Five decades later, are mesenchymal stem cells still relevant? *Front Bioeng Biotechnol* 8: 148

Houlihan DD, Mabuchi Y, Morikawa S, Niibe K, Araki D, Suzuki S, Okano H, Matsuzaki Y (2012) Isolation of mouse mesenchymal stem cells on the basis of expression of Sca-1 and PDGFR-α. *Nat Protoc* 7: 2103–2111

James AM, Hoogewijs K, Logan A, Hall AR, Ding S, Fearnley IM, Murphy MP (2017) Non-enzymatic N-acetylation of lysine residues by AcetylCoA often occurs via a proximal S-acetylated thiol intermediate sensitive to glyoxalase II. *Cell Rep* 18: 2105–2112

Langmead B, Salzberg SL (2012) Fast gapped-read alignment with Bowtie 2. *Nat Methods* 9: 357–359

Lee WD, Mukha D, Aizenshtein E, Shlomi T (2019) Spatial-fluxomics provides a subcellular-compartmentalized view of reductive glutamine metabolism in cancer cells. *Nat Commun* 10: 1351

Leidecker O, Bonfiglio JJ, Colby T, Zhang Q, Atanassov I, Zaja R, Palazzo L, Stockum A, Ahel I, Matic I (2016) Serine is a new target residue for endogenous ADP-ribosylation on histones. *Nat Chem Biol* 12: 998–1000

Leijten J, Georgi N, Moreira Teixeira L, van Blitterswijk CA, Post JN, Karperien M (2014) Metabolic programming of mesenchymal stromal cells by oxygen tension directs chondrogenic cell fate. *Proc Natl Acad Sci USA* 111: 13954–13959

Li H, Handsaker B, Wysoker A, Fennell T, Ruan J, Homer N, Marth G, Abecasis G, Durbin R, 1000 Genome Project Data Processing Subgroup (2009) The sequence alignment/map format and SAMtools. *Bioinformatics* 25: 2078–2079

 © 2022 The Authors

Li Y, Gruber JJ, Litzenburger UM, Zhou Y (2020) Acetate supplementation restores chromatin accessibility and promotes tumor cell differentiation under hypoxia. *Cell Death Dis* 11: 102

Liao Y, Smyth GK, Shi W (2014) featureCounts: an efficient general purpose program for assigning sequence reads to genomic features. *Bioinformatics* 30: 923 – 930

Lu C, Thompson CB (2012) Metabolic regulation of epigenetics. *Cell Metab* 16: 9 – 17

Mews P, Donahue G, Drake AM, Luczak V, Abel T, Berger SL (2017) AcetylCoA synthetase regulates histone acetylation and hippocampal memory. *Nature* 546: 381 – 386

Moore JE, Pratt HE, Purcaro MJ, Weng Z (2020) A curated benchmark of enhancer-gene interactions for evaluating enhancer-target gene prediction methods. *Genome Biol* 21: 17

Morganti C, Bonora M, Marchi S, Ferroni L, Gardin C, Wieckowski MR, Giorgi C, Pinton P, Zavan B (2020) Citrate mediates crosstalk between mitochondria and the nucleus to promote human mesenchymal stem cell in vitro osteogenesis. *Cells* 9: 1034

Morikawa S, Mabuchi Y, Kubota Y, Nagai Y, Niibe K, Hiratsu E, Suzuki S, MiyauchiHara C, Nagoshi N, Sunabori T *et al* (2009) Prospective identification, isolation, and systemic transplantation of multipotent mesenchymal stem cells in murine bone marrow. *J Exp Med* 206: 2483 – 2496

Palmieri EM, Spera I, Menga A, Infantino V, Porcelli V, Iacobazzi V, Pierri CL, Hooper DC, Palmieri F, Castegna A (2015) Acetylation of human mitochondrial citrate carrier modulates mitochondrial citrate/malate exchange activity to sustain NADPH production during macrophage activation. *Biochim Biophys Acta* 1847: 729 – 738

Parekh S, Ziegenhain C, Vieth B, Enard W, Hellmann I (2018) zUMIs - a fast and flexible pipeline to process RNA sequencing data with UMIs. *Gigascience* 7: giy059

Parekkadan B, Milwid JM (2010) Mesenchymal stem cells as therapeutics. *Annu Rev Biomed Eng* 12: 87 – 117

Parrinello S, Samper E, Krtolica A, Goldstein J, Melov S, Campisi J (2003) Oxygen sensitivity severely limits the replicative lifespan of murine fibroblasts. *Nat Cell Biol* 5: 741 – 747

Pattappa G, Thorpe SD, Jegard NC, Heywood HK, de Bruijn JD, Lee DA (2013) Continuous and uninterrupted oxygen tension influences the colony formation and oxidative metabolism of human mesenchymal stem cells. *Tissue Eng Part C Methods* 19: 68 – 79

Pouikli A, Parekh S, Maleszewska M, Nikopoulou C, Baghdadi M, Tripodi I, FolzDonahue K, Hinze Y, Mesaros A, Hoey D *et al* (2021) Chromatin remodeling due to degradation of citrate carrier impairs osteogenesis of aged mesenchymal stem cells. *Nat Aging* 1: 810 – 825

Rappsilber J, Ishihama Y, Mann M (2002) Stop and go extraction tips for matrix-assisted laser desorption/ionization, nanoelectrospray, and LC/MS sample pretreatment in proteomics. *Anal Chem* 75: 663 – 670

Ritchie ME, Phipson B, Wu D, Hu Y, Law CW, Shi W, Smyth GK (2015) Limma powers differential expression analyses for RNA-sequencing and microarray studies. *Nucleic Acids Res* 43: e47

Robinson MD, McCarthy DJ, Smyth GK (2010) edgeR: a bioconductor package for differential expression analysis of digital gene expression data. *Bioinformatics* 26: 139 – 140

Schep AN, Buenrostro JD, Denny SK, Schwartz K, Sherlock G, Greenleaf WJ (2015) Structured nucleosome fingerprints enable high-resolution mapping of chromatin architecture within regulatory regions. *Genome Res* 25: 1757 – 1770

Shyh-Chang N, Daley GQ, Cantley LC (2013) Stem cell metabolism in tissue development and aging. *Development* 140: 2535 – 2547

Spencer JA, Ferraro F, Roussakis E, Klein A, Wu J, Runnels JM, Zaher W, Mortensen LJ, Alt C, Turcotte R *et al* (2014) Direct measurement of local oxygen concentration in the bone marrow of live animals. *Nature* 508: 269 – 273

Sud M, Fahy E, Cotter D, Azam K, Vadivelu I, Burant C, Edison A, Fiehn O, Higashi R, Nair KS *et al* (2016) Metabolomics Workbench: An international repository for metabolomics data and metadata, metabolite standards, protocols, tutorials and training, and analysis tools. *Nucleic Acids Res* 44: D463 – D470

Taudt A, Nguyen MA, Heinig M, Johannes F, Colome-Tatche M (2016) chromstaR: tracking combinatorial chromatin state dynamics in space and time. *bioRxiv* https://doi.org/10.1101/038612 [PREPRINT]

Tessarz P, Kouzarides T (2014) Histone core modifications regulating nucleosome structure and dynamics. *Nat Rev Mol Cell Biol* 15: 703 – 708

Tessarz P, Santos-Rosa H, Robson SC, Sylvestersen KB, Nelson CJ, Nielsen ML, Kouzarides T (2014) Glutamine methylation in histone H2A is an RNA-polymerase-I dedicated modification. *Nature* 505: 564 – 568

Tsai C-F, Wang Y-T, Yen H-Y, Tsou C-C, Ku W-C, Lin P-Y, Chen H-Y, Nesvizhskii AI, Ishihama Y, Chen Y-J (2015) Large-scale determination of absolute phosphorylation stoichiometries in human cells by motif-targeting quantitative proteomics. *Nat Commun* 6: 6622

Weinert BT, Schölz C, Wagner SA, Iesmantavicius V, Su D, Daniel JA, Choudhary C (2013) Lysine succinylation is a frequently occurring modification in prokaryotes and eukaryotes and extensively overlaps with acetylation. *Cell Rep* 4: 842 – 851

Wellen KE, Hatzivassiliou G, Sachdeva UM, Bui TV, Cross JR, Thompson CB (2009) ATP-citrate lyase links cellular metabolism to histone acetylation. *Science* 324: 1076 – 1080

Yang Y-HK, Ogando CR, Wang See C, Chang T-Y, Barabino GA (2018) Changes in phenotype and differentiation potential of human mesenchymal stem cells aging *in vitro*. *Stem Cell Res Ther* 9: 131

License: This is an open access article under the terms of the Creative Commons Attribution License, which permits use, distribution and reproduction in any medium, provided the original work is properly cited.

Expanded View Figures

Figure EV1. Quality control measurements of sequencing libraries.

A, B Insert the size distribution of each ATAC-seq library (two libraries per oxygen condition—A) and mapping statistics for each individual replicate (B).
C, D Correlation matrices of H3K27ac ChIP-seq libraries (C) and H3K4me3 ChIP-seq libraries (D).
E Heatmap of H3K4me3 abundance between hypoxia- and normoxia-cultured MSCs.

Data information: n = 2 biologically independent experiments.

© 2022 The Authors

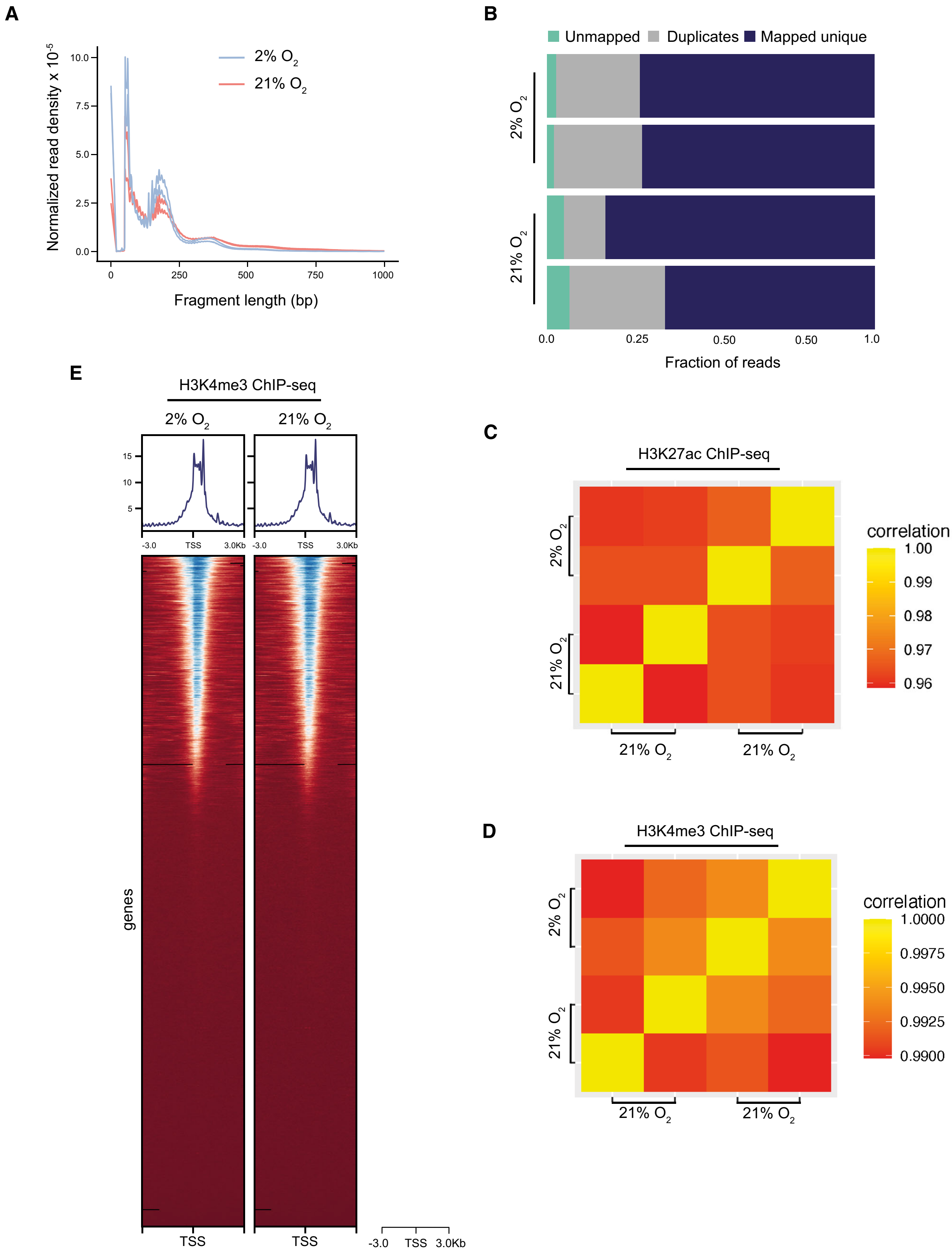
A
Normalized read density x 10-5
10.0
7.5
5.0
2.5
0.0
0
250
500
750
1000
Fragment length (bp)
2% O2
21% O2
B
Unmapped
Duplicates
Mapped unique
2% O2
21% O2
0.0
0.25
0.50
0.50
1.0
Fraction of reads
C
H3K27ac ChIP-seq
correlation
1.00
0.99
0.98
0.97
0.96
2% O2
21% O2
21% O2
21% O2
D
H3K4me3 ChIP-seq
correlation
1.0000
0.9975
0.9950
0.9925
0.9900
2% O2
21% O2
21% O2
21% O2
E
H3K4me3 ChIP-seq
2% O2
21% O2
15
10
5
-3.0
TSS
3.0Kb
genes
TSS
TSS
-3.0
TSS
3.0Kb

Figure EV1.

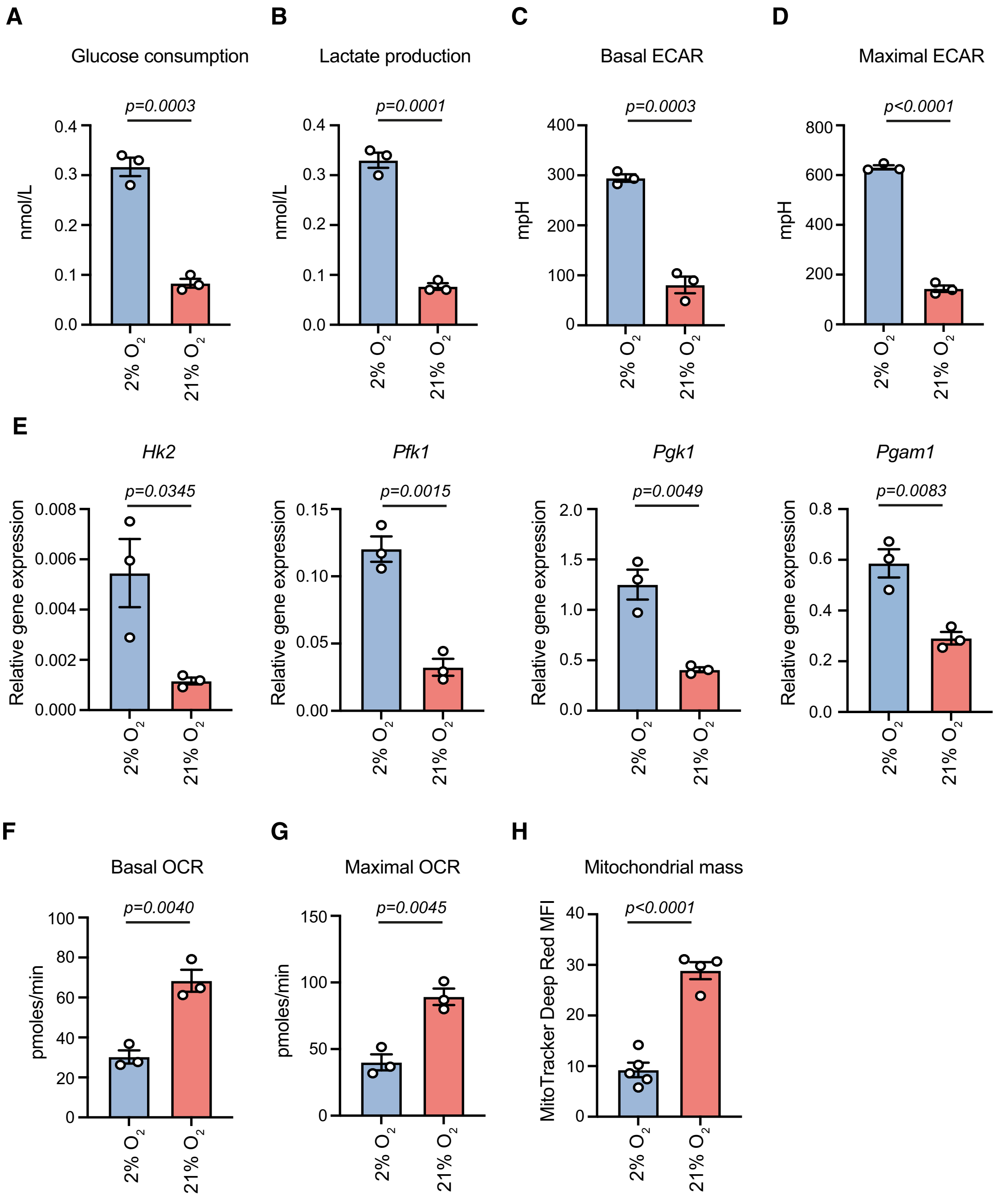

Figure EV2. Metabolic profiling of hypoxic and normoxic MSCs.

A, B Glucose consumption (A) and lactate production (B) were measured in the media of hypoxia- and normoxia-cultured cells using the Vi-Cell MetaFLEX instrument. $n = 3$ biologically independent experiments.

C, D Basal (C) and maximal (D) ECAR in hypoxia- and normoxia-cultured MSCs. $n = 3$ biologically independent experiments.

E qRT-PCR analysis of glycolytic genes in hypoxic and normoxic cells. *β-actin* was used as an internal control for normalization. $n = 3$ biologically independent experiments.

F, G Basal (F) and maximal (G) OCR in hypoxia- and normoxia-cultured MSCs. $n = 3$ biologically independent experiments.

H MFI of hypoxia- and normoxia-cultured cells after staining with the MitoTracker Deep Red FM dye. $n = 4$ biologically independent experiments.

Data information: Results are shown as mean ± SEM and statistical significance was determined using a two-sided unpaired *t*-test.
Source data are available online for this figure.

 © 2022 The Authors

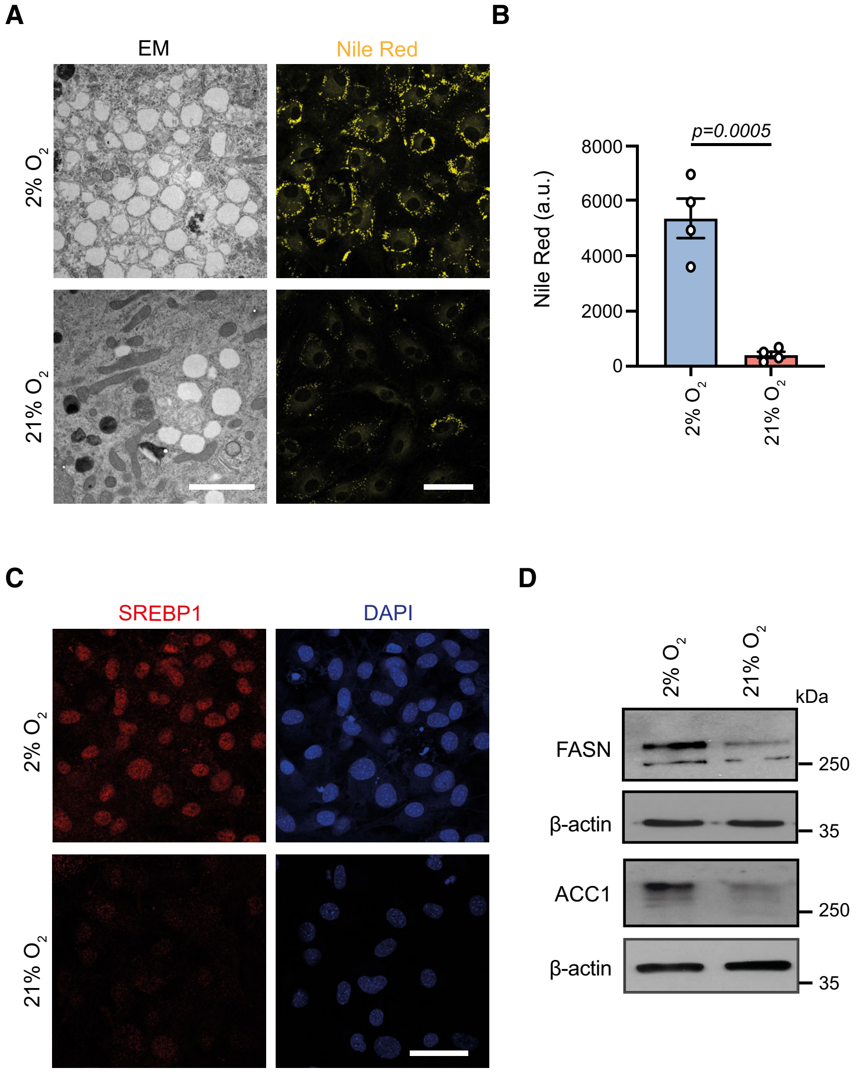

Figure EV3. Impaired lipogenesis of normoxia-cultured MSCs is not due to lower CiC levels.

A, B Representative images (A) and quantification of lipid droplets after observing cells under the electron microscope (left) and after staining lipids with Nile Red (right-B). Scale bars, 2 μm for electron microscopy images and 50 μm for confocal images. $n = 4$ biologically independent experiments and merged results are shown in (B). Results are shown as mean ± SEM and statistical significance was determined using a two-sided unpaired *t*-test.

C Representative images of hypoxia- and normoxia-cultured cells after immunostaining against SREBP1. Scale bar, 50 μm.

D Representative immunoblots against FASN and ACC1 in hypoxia- and normoxia-cultured cells. β-actin was used as a loading control. $n = 3$ biologically independent experiments.

Source data are available online for this figure.

© 2022 The Authors

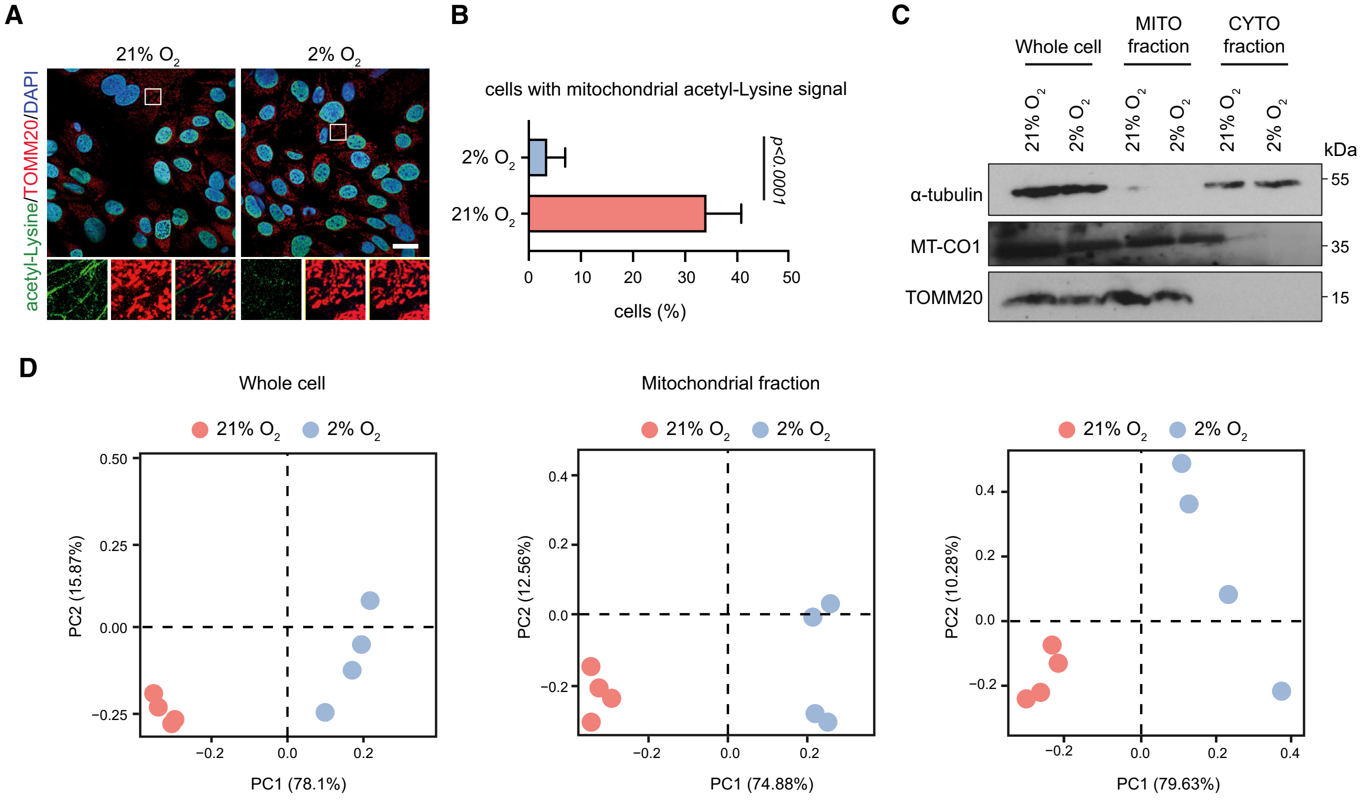

Figure EV4. Quality control measurements of commercial MSCs fractionation.

A, B Representative images after immunostaining of normoxic and hypoxic commercially available MSCs against acetyl-lysine and TOMM20 (A) and assessment of cells (%) with mitochondrial acetyl-lysine signal localization (B). Nuclei were stained with DAPI. Quantification of cells (%) with mitochondrial acetyl-lysine signal in (B) from $n = 201$ hypoxic and $n = 205$ normoxic individual cells from a representative experiment of two biologically independent experiments is shown in (B). Results are shown as mean ± SEM and statistical significance was determined using a two-sided unpaired t-test. In magnified insets, the intensity of the acetyl-lysine signal was adjusted similarly to all samples for visualization purposes. Scale bar, 25 μm.

C Representative immunoblots against the mitochondrial proteins TOMM20 and MT-CO1 and the cytosolic protein α-tubulin in whole lysates, mitochondrial fractions and cytosolic fractions of normoxia- and hypoxia-cultured MSCs. $n = 4$ biologically independent experiments.

D Principal component analysis (PCA) plot showing clustering of normoxia- and hypoxia-cultured cells after metabolite extraction from the whole cell, mitochondrial and cytosolic fractions. $n = 4$ biologically independent experiments.

Source data are available online for this figure.

 © 2022 The Authors

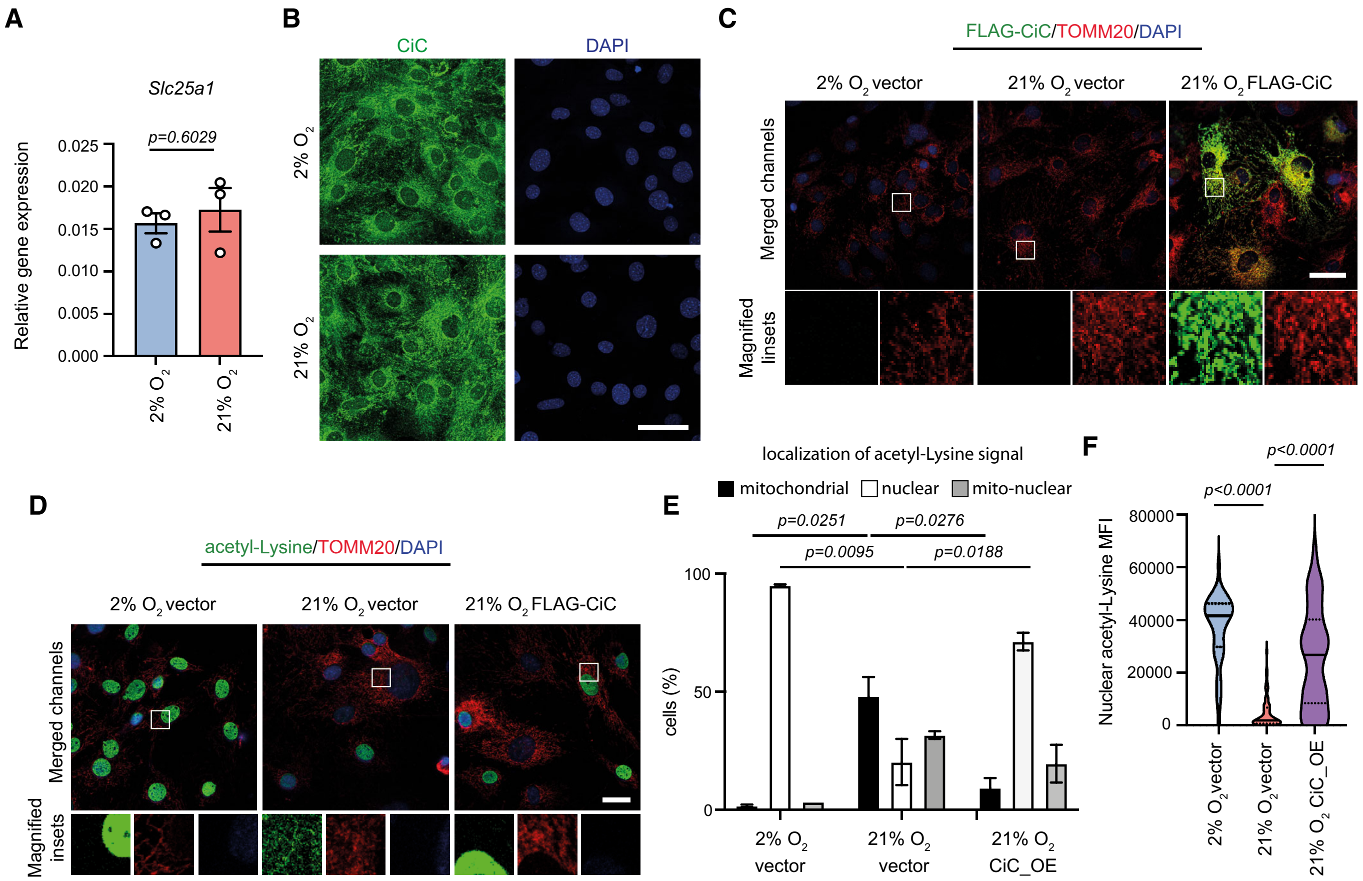

Figure EV5. Impaired CiC function in normoxic MSCs.

A qRT-PCR analysis of *Slc25a1*, which encodes citrate carrier. *β-actin* was used as an internal control for normalization. $n = 3$ biologically independent experiments. Results are shown as mean ± SEM and statistical significance was determined using a two-sided unpaired *t*-test.

B Representative images of hypoxia- and normoxia-cultured cells after immunostaining against CiC. Scale bar, 50 μm.

C Representative images after staining hypoxic and normoxic cells using an anti-FLAG antibody. Cells were transfected with either a vector plasmid or a FLAG-CiC-expressing plasmid. TOMM20 was used as a counterstain for mitochondria to confirm proper localization of the exogenously expressed CiC-FLAG protein, as shown in the magnified inset. Transfection and all downstream experiments were done for $n = 2$ biologically independent experiments. Scale bar; 50 μm.

D–F Representative images after staining cells used in (C) against acetyl-lysine and TOMM20 (D), assessment of the localization, as described above (E), and quantification of nuclear acetyl-lysine signal MFI (F). Nuclei were stained with DAPI. Quantification of nuclear acetyl-lysine MFI from $n = 43$ hypoxic, $n = 63$ normoxic and $n = 38$ normoxic_CiC OE individual cells from a representative experiment of two biologically independent experiments is shown in (F). Results are shown as mean ± SEM and statistical significance was determined with ordinary one-way ANOVA, using the Holm–Sidak's multiple-comparisons test in Panels (E) and (F). The distribution of data points in (F) is shown as a violin plot, where the mean is indicated by a solid line and the quartiles are indicated with dashed lines. In magnified insets, the intensity of the acetyl-lysine signal was adjusted similarly to all samples, for visualization purposes. Scale bar, 25 μm.

Source data are available online for this figure.
