# Supplementary material for: Hypoxia promotes osteogenesis by facilitating acetyl‐CoA‐mediated mitochondrial–nuclear communication
Source: EMBO J. 2022 Oct 24;41(23):e111239. doi: 10.15252/embj.2022111239 (PMC9713713; doi:10.15252/embj.2022111239)
Supplement: Supplementary file 9 [file EMBJ-41-e111239-s001.pdf]

THE EMBO JOURNAL

Hypoxia promotes osteogenesis by ensuring acetyl-CoA-mediated mitochondrial-nuclear communication

Andromachi Pouikli, Monika Maleszewska, Swati Parekh, Ming Yang, Chrysa Nikopoulou, Juan Jose Bonfiglio, Constantine Mylonas, Tonantzi Sandoval, Anna-Lena Schumacher, Yvonne Hinze, Ivan Matic, Christian Frezza, and Peter Tessarz **DOI: 10.15252/embj.2022111239**

Corresponding author: Peter Tessarz (ptessarz@age.mpg.de)

Review Timeline:

Submission Date:	22nd Mar 22
Editorial Decision:	27th Apr 22
Revision Received:	28th Jul 22
Editorial Decision:	8th Sep 22
Revision Received:	17th Sep 22
Accepted:	23rd Sep 22

Editor: Kelly Anderson

Transaction Report:

(Note: With the exception of the correction of typographical or spelling errors that could be a source of ambiguity, letters and reports are not edited. Depending on transfer agreements, referee reports obtained elsewhere may or may not be included in this compilation. Referee reports are anonymous unless the Referee chooses to sign their reports.)

Dear Dr. Tessarz,

Thank you for submitting your manuscript for consideration by the EMBO Journal. It has now been seen by three referees whose comments are shown below. As you will see from the reports, there is general interest in your findings. That said, there were significant concerns raised regarding whether the proposed mechanism (changes in CiC activity) was sufficiently supported by the data in the current version of the manuscript.

Should you be able to address these criticisms in full, we would consider a revised manuscript. It is EMBO Journal policy to allow a single round of revision only and therefore, acceptance or rejection of the manuscript will depend upon the completeness of your responses in this revised version. It would be good to discuss your plan for revision and I am available to do so via email or zoom in the next few weeks.

In your revised manuscript, please include a detailed point-by-point response to the referees' comments. Also, please bear in mind that this will form part of the Review Process File, and will therefore be available online to the community. For more details on our Transparent Editorial Process, please visit our website: https://www.embo.org/embo-press

We generally allow three months as standard revision time. As a matter of policy, competing manuscripts published during this period will not negatively impact our assessment of the conceptual advance presented by your study. However, we request that you contact the editor as soon as possible upon publication of any related work, to discuss how to proceed. Should you foresee a problem in meeting this three-month deadline, please let us know in advance and we may be able to grant an extension. I have attached a guide for revisions for your convenience, but more information can be found in our guide to authors (link below).

Thank you for the opportunity to consider your work for publication. I look forward to your revision.

Yours sincerely,
Kelly

Kelly M Anderson, PhD
Editor
The EMBO Journal
k.anderson@embojournal.org

Referee #1:

This work compared the effect of 21% and 2% O2 on bone marrow MSCs in vitro. The Authors observed that incubation at 21% O2 decreased osteogenic differentiation, expression of osteogenic marker genes, and reduced chromatin accessibility on osteogenic gene promoters. This was accompanied by reduced histone acetylation even though Ac-CoA, a substrate for acetylation reactions, was higher at 21% O2-exposed cells. They suggested that this was due to decreased activity of CiC (citrate carrier) in mitochondria and trapping of Ac-CoA in mitochondria. The Authors claim that the evidence for that is higher staining of ac-Lysine in mitochondria.
The strength of this work is the robust analysis of chromatin architecture and epigenome in MSCs under various O2 levels. With regards to general impact of this work, it is somewhat limited as this relates mostly to incubation of cells in vitro. In vivo scenario where O2 is at 21% is not realistic. Rather, this may be important for optimization of MSCs culture intended for stem cell therapy. My main concern is the conclusion that the observed effects are due to trapping of AcCoA in mito due to lower CiC activity. First, AcCoA in mito was not directly measured. Higher observed mito Ac-Lys signal could as also be due to lower activity of mito deacetylases, such as Sirt3, or higher activity of acetyl transferases. AcCoA can be measured directly in mito fraction vs cytosolic fraction. Second, CiC activity was not directly measured either. This could be done by giving cells labeled TCA cycle substrate and then detecting presence of cytosolic labeled citrate or AcCoA. Digitonin used at low concentrations would allow for quick release of cytosolic metabolites for the assay without affecting mito metabolites. Third, there could be other reasons for citrate trapping in mito, e.g. lower activity of ACLY. This could lead to accumulation of citrate in the cytosol and inhibition of its efflux from mito.

The above concerns need to be addressed before this paper is acceptable for publication.

Referee #2:

The manuscript focused on the flux of acetyl-CoA from mitochondria to the nucleus, and oxygen level changes in the flow of acetyl-CoA affect stem cell function. Upon shifting cells from low to high oxygen, there is a switch in the subcellular localization of acetyl-CoA, which affects the epigenetic landscape. CiC was regard as a novel, oxygen-sensitive regulator of MSC function. The theme discussed is novelty, and the conclusions is feasible. Thus, this reviewer suggests to accept the manuscript.

Referee #3:

Pouikli et al. reported hypoxic condition promoted osteogenesis via accumulation of acetyl-CoA and increasing histone acetylation. Although this work was novel and interesting, there are still numbers of questions that the authors need to address.

The main concern is that this paper did not address how hypoxia facilitates acetyl-CoA escaping the trap from mitochondria compared to normoxia. The majority of the bio-informatic data provided good relevance between the differentiation of MSCs and hypoxic condition, through histone modification. However, the biological evidence was not convincing.

1. Based on the title, the authors want to emphasize the communication between mitochondria and nucleus, which direct me want to explore more than only acetyl-CoA. This paper mainly focused on the distribution and contribution of acetyl-CoA in osteogenesis in response to hypoxia. Why not just point it out in the title?

2. In this paper, the authors used BM-MSCs as the cell type. Is that necessary to remove the bone marrow based on the schematic representation in figure 1a? If only the MSCs derived from bone tissue were used in the experiments, the term BM-MSCs was not correct.

3. In figure 1 d and e, I assume these cells were incubated with an osteogenic medium, and data were collected after the staining. What's the exact meaning of "Area" here? There is barely any staining in normoxic condition even after a 7-days induction. Is that repeatable? Normally, the MSCs cultured in normoxia still gain the osteogenic compacity when you treat them with an osteogenic medium. Why here is it not?

4. In figure 2, the chromatin accessibility changed by hypoxia or normoxia was not surprising. Emerging evidence has been proving it.

5. In figure 3, panel b, please explain the more mechanism of methylation alteration compared between hypoxia and normoxia. Or at least discuss in details.

6. Figure 3i, did the authors measure the total acetyl-CoA or the one isolated from the nucleus? The text matched panel J well, but not panel I in the manuscript.

7. Figure 4, what is the mechanism that acetyl-CoA was trapped in the mitochondria? And how is it achieved in normoxia? The changes of CiC activity cannot be the whole story.

8. Minor comment:
Some info about the chemical compound was missing such as the commercial name and manufacture of BTA.

We would like to thank the reviewers for the constructive and insightful comments on our manuscript. We have now carefully addressed all of their concerns and we trust that these changes have significantly improved our manuscript and strengthened our conclusions. In particular, we now i) provide new evidence to corroborate the mitochondrial acetyl-CoA accumulation in normoxia-cultured MSCs, ii) clarify the role of CiC in this process, and iii) expand our Materials and Methods section, so that it includes more information about the overall experimental setup and more details about the cell systems we used.

To facilitate the evaluation of our revised manuscript, we reference the old and new figures and panel numbers as well as the line numbers in the new version of the text, in all instances.

Please find the point-by-point responses below (reviewers' comments in *italic*).

Referee #1:

This work compared the effect of 21% and 2% O2 on bone marrow MSCs in vitro. The Authors observed that incubation at 21% O2 decreased osteogenic differentiation, expression of osteogenic marker genes, and reduced chromatin accessibility on osteogenic gene promoters. This was accompanied by reduced histone acetylation even though Ac-CoA, a substrate for acetylation reactions, was higher at 21% O2-exposed cells. They suggested that this was due to decreased activity of CiC (citrate carrier) in mitochondria and trapping of Ac-CoA in mitochondria. The Authors claim that the evidence for that is higher staining of ac-Lysine in mitochondria. The strength of this work is the robust analysis of chromatin architecture and epigenome in MSCs under various O_2 *levels.*
With regards to general impact of this work, it is somewhat limited as this relates mostly to incubation of cells in vitro. In vivo scenario where O_2 *is at 21% is not realistic. Rather, this may be important for optimization of MSCs culture intended for stem cell therapy.*

Response: We would like to thank the reviewer for appreciating our effort to comprehensively characterize the epigenetic changes that occur in MSCs, in response to culture under high oxygen levels. We fully agree with the reviewer that an *in vivo*

environment characterized by the presence of 21% O_2 does not exist. However, MSCs are extensively used in the field of regenerative medicine and maintenance of their differentiation capacity is crucial for the success of stem cell therapies and tissue engineering approaches. In such therapeutic approaches, MSCs are isolated form their *in vivo* hypoxic environment and are expanded *in vitro* for many population doublings, under atmospheric oxygen conditions. With our study, we shed light on the profound and irreversible oxygen-induced changes in metabolism, chromatin and stem cell function, which should be taken into account both during the clinical use of this stem cell population but also in research, when exposing cells to different oxygen conditions. Therefore, we strongly believe that our manuscript will be of great interest both to the stem cell community, and to a wider cell and molecular biology readership.

My main concern is the conclusion that the observed effects are due to trapping of AcCoA in mito due to lower CiC activity. First, AcCoA in mito was not directly measured. Higher observed mito Ac-Lys signal could as also be due to lower activity of mito deacetylases, such as Sirt3, or higher activity of acetyl transferases. AcCoA can be measured directly in mito fraction vs cytosolic fraction. Second, CiC activity was not directly measured either. This could be done by giving cells labeled TCA cycle substrate and then detecting presence of cytosolic labeled citrate or AcCoA. Digitonin used at low concentrations would allow for quick release of cytosolic metabolites for the assay without affecting mito metabolites. Third, there could be other reasons for citrate trapping in mito, e.g. lower activity of ACLY. This could lead to accumulation of citrate in the cytosol and inhibition of its efflux from mito. The above concerns need to be addressed before this paper is acceptable for publication.

<u>Response:</u> We agree with the reviewer that other processes, including lower activity of mitochondrial deacetylases or higher activity of acetyltransferases, could be contributing to the phenotype we described in our study. To address this point and the concerns raised by the reviewer, we now provide new data which, in combination with our previous results, further support our model regarding impaired CiC function leading to mitochondrial accumulation of acetyl-CoA, thus reduced histone acetylation.

First, as suggested by the referee, we performed metabolomic experiments to measure the intracellular distribution of acetyl-CoA. Since primary MSCs represent a rare stem cell population and we are only able to isolate a maximum of ~100k pure MSCs per three mice, which renders big-scale metabolomic experiments unfeasible, we resorted to a commercially available cell line of MSCs (for details about this cell line please be referred to the 'Materials and Method' section of our revised manuscript and the 'Reagents_Tool' table submitted with our manuscript). These cells have been expanded for many passages under high oxygen conditions, thus they have adapted to normoxia. Upon exposure and culture in hypoxia, they exhibited epigenetic and metabolic changes that reflect some of the observations made in primary cells, such as the restored flux of acetyl-CoA from mitochondria to the cytosol/nucleus (new **Figure EV4A-EV4B**). Using these cells, we measured the concentration of acetyl-CoA and citrate in normoxic and hypoxic cells, following a published fractionation protocol based on digitonin (PMID: 30903027). Confirming our previous results from experiments with primary cells, we found that normoxia-cultured MSCs contained: i) increased total levels of acetyl-CoA and citrate, ii) higher levels of mitochondrial acetyl-CoA and citrate, and iii) lower levels of cytosolic acetyl-CoA and citrate (new **Figure 4C-4D**). These results confirm that in normoxia-cultured MSCs, acetyl-CoA and citrate accumulate inside mitochondria and that there is an impaired flux of these metabolites to the cytosol.

Second, to corroborate our model, where acetyl-CoA trapping inside mitochondria occurs due to lower CiC function, we performed tracing experiments and labelled normoxic and hypoxic cells with the [4-^{2}H]Glucose tracer (for details about the labelling strategy please be referred to PMID:35264789). Mitochondria-produced citrate is exported to the cytosol via the CiC and generates malate. Cytosolic citrate produces malate via the co-ordinated action of two enzymes: i) ATP-citrate-lyase (ACLY) cleaves citrate to acetyl-CoA and oxaloacetate (OAA), and ii) malate dehydrogenase (MDH) converts OAA to malate. The conversion of OAA to malate requires hydride donation from cytosolic NADH. Glycolytic processing of [4-^{2}H]Glucose in the cytosol leads to deuterated cytosolic NADH, which when used by MDH produces deuterated malate (hereafter indicated as malate m+1) (**R-Figure 1**). Therefore, levels of malate m+1 are proportional to the levels of citrate exported from mitochondria to the cytosol. Given that hypoxia alters dramatically the energetic profile and the abundance

of intracellular metabolites, to correct for potential changes in total cellular citrate between the two conditions, we normalized the malate m+1 levels to the total citrate levels. This comparison revealed **lower malate m+1:citrate ratio in normoxia-cultured MSCs** further supporting the reduced citrate export from mitochondria, hence impaired CiC activity in these conditions (**R-Figure 1**).

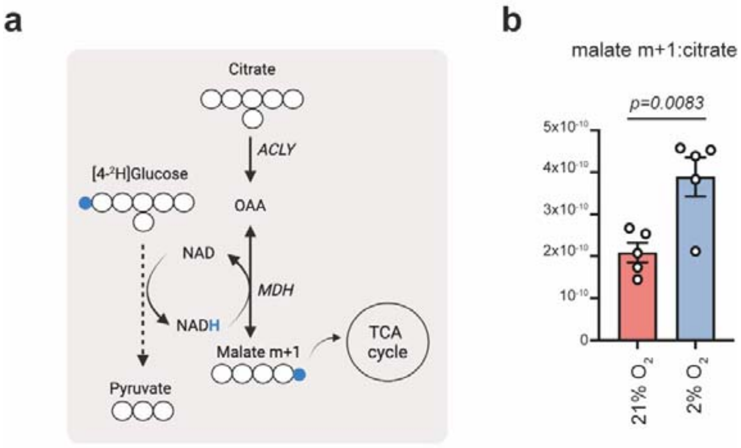

R-Figure 1: Metabolic labeling using the [4-^{2}H]Glucose tracer. (a) Schematic representation illustrating the transfer of labelled deuterium from glucose to NADH during glycolysis and to malate during OAA processing by MDH. (**b**) Ratio of [4-2H]-labelled malate m+1:citrate in normoxic and hypoxic cells; n=5 biologically independent replicates and results are shown as mean ± S.E.M.

In addition, as an independent and direct approach to strengthen our conclusion that impaired CiC function is the main mechanism leading to accumulation of acetyl-CoA inside mitochondria, we used lentiviral transduction to exogenously express CiC in normoxic MSCs. After confirming that the over-expressed CiC protein localized properly to mitochondria (new **Figure EV5C**), we next used these cells to repeat the acetyl-Lysine/TOMM20 immunostaining experiment. We found that elevated CiC levels redistributed acetyl-CoA from mitochondria to the nucleus, as evidenced by the decreased acetylation of mitochondrial proteins and the concomitantly enhanced nuclear/histone acetylation, similarly to the behaviour of hypoxic MSCs (new **Figure EV5D-EV5F**). Thus, exogenous CiC over-expression restores acetyl-CoA flux from mitochondria to cytosol/nucleus. Consistent with this, we previously reported that pharmacological inhibition of CiC activity using BTA directly influenced the mitochondrial/cytosolic abundance of acetyl-CoA in hypoxic cells, with BTA treatment leading to higher acetylation of mitochondrial proteins and lower acetylation of nuclear proteins, thus increased mitochondrial acetyl-CoA localization (**Figure**

5D-5F). The phenotype of the BTA-treated hypoxic MSCs resembles that of normoxic MSCs. Since BTA is a specific inhibitor of CiC and inhibition of CiC function alone is able and sufficient to make hypoxic cells behave like the normoxic, at least with regards to the distribution of acetyl-CoA, these results validate that accumulation of acetyl-CoA within mitochondria is due to impaired CiC function and highlight the causal relation between low CiC function and mitochondrial acetyl-CoA accumulation.

Given the low cell numbers that we can work with, we cannot perform classical activity assays for HAT/HDAC. However, to approach this concern, we compared protein levels of major acetyltransferases and de-acetylases, such as CBP, GCN5 and SIRT3, between normoxia- and hypoxia-cultured primary MSCs. We did not observe any change in the protein levels of the SIRT3 mitochondrial deacetylase or the levels of the GCN5 histone acetyltransferase, whereas levels of the CBP histone acetyltransferase were higher in normoxia-cultured MSCs compared to hypoxic cells (**R-Figure 2**). Although these findings do not exclude potential oxygen-induced changes in the activity of these proteins (which would be infeasible to address in our stem cell system), our results suggest that the observed changes are likely-independent of the acetyltransferases and de-acetylases.

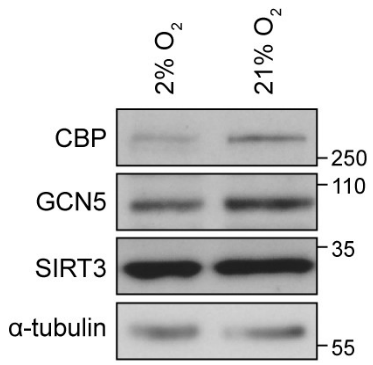

R-Figure 2: Assessment of levels of histone acetyltransferases and deacetylases. Representative immunoblots for CBP, GCN5 and SIRT3. α-tubulin was used as a loading control; n=3 biological replicates.

Collectively, our data favor a model where impaired export of acetyl-CoA and citrate from mitochondria to the cytosol due to lower CiC activity leads to trapping of acetyl-CoA inside

mitochondria, higher acetylation of mitochondrial proteins and lower acetylation of nuclear proteins.

Referee #2

The manuscript focused on the flux of acetyl-CoA from mitochondria to the nucleus, and oxygen level changes in the flow of acetyl-CoA affect stem cell function. Upon shifting cells from low to high oxygen, there is a switch in the subcellular localization of acetyl-CoA, which affects the epigenetic landscape. CiC was regard as a novel, oxygen-sensitive regulator of MSC function. The theme discussed is novelty, and the conclusions is feasible. Thus, this reviewer suggests to accept the manuscript.

Response: We would like to thank the reviewer for the positive evaluation of our work.

Referee #3:

Pouikli et al. reported hypoxic condition promoted osteogenesis via accumulation of acetyl-CoA and increasing histone acetylation. Although this work was novel and interesting, there are still numbers of questions that the authors need to address.
The main concern is that this paper did not address how hypoxia facilitates acetyl-CoA escaping the trap from mitochondria compared to normoxia. The majority of the bio-informatic data provided good relevance between the differentiation of MSCs and hypoxic condition, through histone modification. However, the biological evidence was not convincing.

Response: We would like to thank the reviewer for the positive assessment of our study. To provide stronger biological evidence, we performed new experiments and our work now presents thorough mechanistic insights on the CiC-mediated regulation of the chromatin landscape, in response to high oxygen levels.

1. Based on the title, the authors want to emphasize the communication between mitochondria and nucleus, which direct me want to explore more than only acetyl-CoA. This

paper mainly focused on the distribution and contribution of acetyl-CoA in osteogenesis in response to hypoxia. Why not just point it out in the title?

Response: Following the reviewer's suggestion we modified the title of our manuscript to specify that we focused on acetyl-CoA. Our new title is 'Hypoxia promotes osteogenesis via regulating the acetyl-CoA-mediated mito-nuclear communication'.

2. In this paper, the authors used BM-MSCs as the cell type. Is that necessary to remove the bone marrow based on the schematic representation in figure 1a? If only the MSCs derived from bone tissue were used in the experiments, the term BM-MSCs was not correct.

Response: Depletion of the bone marrow is indeed a critical step during the process of MSC isolation from the murine bone endosteum, since it minimizes the contamination by hematopoietic cells (PMID: 23154782). Therefore, removal of the bone marrow ensures high purity and homogeneity of isolated MSCs. As per the reviewer's request, we replaced the term 'BM-MSCs' with 'bone-MSCs', to better reflect the origin of the stem cell population that we used in our study.

3. In figure 1 d and e, I assume these cells were incubated with an osteogenic medium, and data were collected after the staining. What's the exact meaning of "Area" here? There is barely any staining in normoxic condition even after a 7-days induction. Is that repeatable? Normally, the MSCs cultured in normoxia still gain the osteogenic compacity when you treat them with an osteogenic medium. Why here is it not?

Response: For the experiments shown in Figures 1D and 1E (as well as for those in Figures 5G and 5H), cells were cultured in 96-well plates under hypoxic or normoxic conditions in control (MEM-Alpha supplemented with 10% FBS and 1% penicillin/streptomycin) or osteogenesis medium (control medium supplemented with 100 nM dexamethasone, 10 mM betaglycerophosphate and 100 µM ascorbic acid), for 11 days. Osteogenic differentiation was confirmed after staining cells with Alizarin Red S and imaging was done by randomly choosing 3-5 fields in every single sample. Quantification of Alizarin Red staining was done in ImageJ, as described in the 'Image acquisition and processing' section, of our revised

manuscript (lines 800-802), after applying image thresholding criteria similarly to all samples and results are shown in Figure 1e. We would like to highlight that although quantification of the Alizarin Red S staining can be also performed by acetic-extraction of the dye, this method yielded unreliable results in our hands, most likely due to technical issues related to the insufficient extraction of the dye from 96-well plates.

The profound loss of the osteogenic differentiation capacity upon exposure of cells to normoxic conditions was highly reproducible and observed each time the experiment was repeated (n=3 biological replicates for hypoxic samples; n=5 biological replicates for normoxic and reversed-hypoxic samples). The dramatic loss of the osteogenic differentiation capacity that we observed in normoxic cells, is in line with previously published reports (e.g., PMID: 23029528, PMID: 31827540, PMID: 25109357) demonstrating strong defects in osteogenesis of normoxia-cultured MSCs. However, we agree with the reviewer that some studies have shown osteogenic differentiation capacity under normoxic conditions. We speculate that the profound loss of osteogenic potential that we report here might be explained by:
i) the unique properties of the specific stem cell population used in each study. Here we used a very stringent, FACS-based approach to isolate pure MSCs, whereas other studies are based on the ‘plastic-adherence’ method that results in a ‘contaminated’ MSC population,
ii) the exact oxygen conditions under which the cells were cultured. Although we cultured MSCs under 2% O_2, which resembles the oxygen concentration found in their niche, in several reports the authors culture MSCs under extreme hypoxic conditions, i.e., less than 1% O_2, which might affect differently the stem cell behavior,
iii) the duration of the exposure to different oxygen conditions. Long- or short-term exposure to high oxygen tension results in different phenotypes and affects differently the stem cell function, as has been previously reported (e.g., PMID: 25034305),
iv) the direction of the shift in the oxygen tension. Although MSCs in several studies are cultured under high oxygen levels and are then moved to hypoxic conditions, here we followed the opposite approach. Thus, we initially expanded these cells under hypoxia, to mimic the oxygen tension found in their physiological niche, and we then shifted them to normoxia, and

v) the exact timepoint at which the osteogenic differentiation was assessed. In most studies the osteogenic differentiation is evaluated 21 days after induction of differentiation. However, we focused on an earlier timepoint and assessed differences in osteogenesis 11 days after inducing differentiation.

All these parameters could dramatically alter MSC activity, contributing to the profound decrease in the osteogenic differentiation capacity that we observed in normoxic MSCs.

4. In figure 2, the chromatin accessibility changed by hypoxia or normoxia was not surprising. Emerging evidence has been proving it.

Response: Indeed, there have been few reports suggesting that changes in the oxygen concentration impact the chromatin landscape (e.g., PMID: 32029721, PMID: 35258521), to which we refer in the revised version of our manuscript (lines 125-127). However, we would like to emphasize that there is no consensus on the direction of the change in chromatin accessibility upon switch in the levels of O_2, with these two studies demonstrating opposite effects. This suggests that the oxygen-induced alterations in the chromatin architecture might be cell-type specific. Since there is no published data on MSCs, we strongly believe that our study provides novel insights on the effects of oxygen in the regulation of chromatin accessibility in bone-derived MSCs.

5. In figure 3, panel b, please explain the more mechanism of methylation alteration compared between hypoxia and normoxia. Or at least discuss in details.

Response: We agree with the reviewer that the increased histone methylation in normoxia-cultured MSCs is an interesting observation. However, since in our study we focused exclusively on the acetyl-CoA-histone acetylation axis (as also indicated in our updated manuscript title), we haven’t investigated this in more detail. Nevertheless, one potential explanation could be the trend towards lower cytosolic levels of α-KG in normoxic cells (**R_Figure 3**). Lower α-KG levels could lead to reduced activity of histone demethylases, thus, explaining the increased histone methylation we observed in our system.

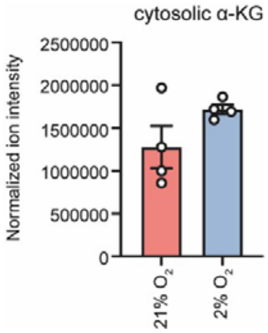

R-Figure 3: α-KG levels in normoxic and hypoxic MSCs. LC/MS analysis of cytosolic α-KG levels in normoxia- and hypoxia-cultured MSCs, after cell fractionation. n=4 biological replicates. Data are presented as mean ± SEM.

Notably, a recent study also reported a similar increase in histone methylation, and especially in the H3K9me3 mark, in MSCs with impaired CiC activity, directly linking mitochondrial acetyl-CoA to histone methylation and osteogenesis (PMID: 32326298). We now comment on this further in the revised version of the manuscript (lines 165-167 and 330-334). In line with this, we have previously reported a similar phenotype in aged MSCs, characterized by mitochondrial acetyl-CoA accumulation and increased H3K27me3 levels (Pouikli et al., 2021: https://www.nature.com/articles/s43587-021-00105-8).

6. Figure 3i, did the authors measure the total acetyl-CoA or the one isolated from the nucleus? The text matched panel J well, but not panel I in the manuscript.

Response: In the initial version of our manuscript, we had measured total acetyl-CoA levels using primary MSCs (**Figure 3I).** In addition to this, we have now measured acetyl-CoA (and citrate) levels both in the mitochondrial and in the cytosolic fractions of commercially available MSCs, cultured in normoxia or hypoxia for 7 days (new **Figure 4D**).

7. Figure 4, what is the mechanism that acetyl-CoA was trapped in the mitochondria? And how is it achieved in normoxia? The changes of CiC activity cannot be the whole story.

Response: We agree with the reviewer that it would be interesting to identify how CiC activity is regulated in response to high oxygen in order to better understand how acetyl-CoA trapping inside mitochondria is achieved. As discussed in the respective field of our

manuscript (lines 340-353), CiC function can be regulated by post-translational modifications (PTMs). Therefore, we speculate that specific PTMs are involved in the regulation of CiC activity, in response to high oxygen levels. However, given the low abundance of bone-derived MSCs, a structure-function approach, or the identification of the exact PTMs that could be responsible for the compromised CiC function in normoxic cells is infeasible in this system and also beyond the scope of this study. Nevertheless, we performed new experiments, which together with the previously shown results demonstrate that the major reason for the accumulation of acetyl-CoA within the mitochondria of normoxic cells is the reduced/impaired activity of CiC. In particular, we show now that lower CiC function is causally related to mitochondrial acetyl-CoA accumulation in normoxic MSCs, because:

i) Pharmacological inhibition of the CiC function using the BTA inhibitor resulted in trapping of acetyl-CoA within mitochondria, as evidenced by enhanced mitochondrial acetyl-Lysine signal and reduced nuclear acetyl-Lysine signal (**Figure 5D-5F**),

ii) Supplementation of normoxic cells with sodium acetate, to circumvent impaired CiC activity, restored the cytosolic/nuclear availability of acetyl-CoA (**Figure 5D-5F**),

iii) Exogenous CiC expression in normoxia-cultured MSCs reduced the acetylation of mitochondrial proteins and re-distributed the acetyl-Lysine signal into the cytosol/nucleus (**Figure EV5D-EV5F**),

iv) Along with the changes in the acetyl-Lysine signal, pharmacological perturbations in CiC function directly influenced the lipid biogenesis pathway, indicating that manipulating CiC activity impacts the total cytosolic acetyl-CoA pool, and not just the establishment of histone acetylation (**Figure 5B-5C**),

v) The reduced malate m+1:citrate ratio in normoxic cells, indicated that it is the impaired CiC activity which leads to acetyl-CoA trapping within the mitochondria of the normoxic cells (**R-Figure 1**). As mentioned above (please see response to reviewer #1) the malate m+1:citrate ratio is indicative of the CiC function and levels of the malate m+1 strongly depend on CiC exporting activity.

Together, our data highlight the central role of CiC in the regulation of the mitochondrial and cytosolic/nuclear acetyl-CoA pools. However, we agree with the reviewer that other factors could be also involved in the impaired export of acetyl-CoA to the cytosol and the

concomitant changes in the osteogenic potential, since high oxygen levels elicit profound changes in various biological processes, including cell metabolism, that could also impact stem cell fate decisions. We comment on this now in the discussion of the revised version of our manuscript (lines 350-353).

8. Minor comment:

Some info about the chemical compound was missing such as the commercial name and manufacture of BTA.

Response: As per the reviewer's request, this information has been added in our revised manuscript for all the compounds/reagents used in this study (please see the 'Reagents_Tool' table submitted with our manuscript).

1st Revision - Editorial Decision 8th Sep 2022

Dear Peter,

Congratulations on a great revision! Overall, the referees have been positive. However, as we previously discussed, please include the Cox1 blot to demonstrate the quality of mitochondria and provide responses to the other comments in a new point-by-point response.

When you submit your revised version, please also take care of the following editorial items and add this also to your point-by-point response:

1. Please review our new policy on conflict on interests on the EMBO author guide website and update the title of this section to: Disclosure and competing interest statement

2. Please remove author credit/contributions section from the manuscript file

3. There is no reference to figure EV1D or E in the manuscript, please include them in the correct order.

4. The EV table should be renamed to Dataset EV1 as it has too many columns. Please also update the callout of the figure in the manuscript.

5. Thank you for providing source data. However, please instead provide them as one PDF file per figure labeled with the appropriate figure/panel number with them ZIPed together.

6. "Methods" should be corrected to Materials and Methods

7. The heading "Expanded View Figure Legends" should be added.

8. The EV table legends should be removed from the manuscript file.

Thank you for the opportunity to consider your work for publication. I look forward to your revision.

Kind regards,
Kelly

Kelly M Anderson, PhD
Editor
The EMBO Journal
k.anderson@embojournal.org

Referee #1:

In this revision, Pouikli et al presented new data to confirm entrapment of AcCoA in mitochondria (mito) due to CiC inhibition as an explanation for the observed acetylation changes and chromatin accessibility in BMSCs under 21% vs 2% O2.
In the previous submission, the question was raised of why their cells are not osteoinduced at 21% as well as at 2% O2 while many other studies show efficient OB differentiation at 21% O2. Why this is different from a variety of other studies that do not report any inhibition of osteogenic differentiation at normoxia is still not well explained. It may be due to the fact that the Authors here look at a subset of BMSCs as not all of them are Sca1 and 140a positive according to many previous reports e.g. from D Scadden group. This should be discussed in more detail as a limitation of this study. Moreover, when the Authors show Nile Red staining in these cells to prove changes in lipid metabolism, these cells look like adipocytes even without adipogenic induction. The explanation here can be that 2% hypoxia pushes them towards adipogenic lineage in uninduced conditions. What is the level of adipogenic markers in these cells at baseline in regular media?
Another concern was raised before, that there was no direct evidence of changes of AcCoA pool in mito and cytosol. It was suggested to use digitonin permeabilization technique to obtain mito and cytosolic fractions. For cell fractionation with digitonin,

the Authors followed a previously published protocol. However, why that particular protocol was chosen is not clear. First, they used PBS in their permeabilization media which is not appropriate media for mitochondria. Pls see a review by Brand and Nicholls (doi:10.1042/BJ20110162) for appropriate media. It should be potassium-based and have various other components necessary for mitochondria. Moreover, 1 mg/ml digitonin, which is 0.1% solution is quite high and can permeabilize mitochondrial membrane. Usually 0.01% is sufficient to permeabilize plasma membrane. Mitotracker retention used in the paper by Lee et al is not an appropriate method to assess mitochondrial integrity after treatment with digitonin. Once Mitotracker is accumulated in mito, it forms disulfide bonds to proteins and stays there even if mito integrity is compromised (see above review for details). Blotting for tubulin and TOMM20 used by the Authors, does not guarantee 'purity' of fractions since tubulin is a structural protein, not capable of moving freely into the mito while Tomm20 is a mito membrane protein, that is not expected to be released into cytosol even if the mito membrane is permeabilized. Moreover, permeabilization may not be sufficient to release proteins but just small metabolites. Therefore, fig 4 experiments and other data dependent on this technique remain questionable.
Again, ac-lysine immunofluorescence was used as a marker of increased acetylation and AcCoA trapping in mito as shown in Fig 5. In fact, the magnified regions in fig 5D show nuclear and non-nuclear distribution of ac-lysine depending on the condition. How mitochondrial signal is defined is not clear as there is very little co-localization of TOMM20 and ac-lysine signals. Therefore, the conclusion that there is increased ac-lysine in mito and trapping of AcCoA in mito is questionable.
In sum, the data on chromatin accessibility in 2 vs 21% O2 in BMSCs is very interesting, however the mechanistic explanation for the observed differences is questionable. Moreover, it may also be applicable only to a subset of BMSCs (Sca1+ cd140+).

Referee #3:

Pouikli et al. reported hypoxic conditions promoted osteogenesis via accumulation of acetyl-CoA and increasing histone acetylation, which is intriguing and vital in the field. The revised vision answered all the questions raised previously. We recommend this work should be accepted.

We would like to thank the reviewers for evaluating our revised manuscript and we are happy that they appreciate our efforts to address the concerns raised.

To facilitate the evaluation of our revised manuscript, we reference the old and new figures and panel numbers as well as the line numbers in the new version of the manuscript, in all instances.

Please find the point-by-point responses below (reviewer's comments in italic).

Referee#1:

In this revision, Pouikli et al presented new data to confirm entrapment of AcCoA in mitochondria (mito) due to CiC inhibition as an explanation for the observed acetylation changes and chromatin accessibility in BMSCs under 21% vs 2% O2. In the previous submission, the question was raised of why their cells are not osteoinduced at 21% as well as at 2% O2 while many other studies show efficient OB differentiation at 21% O2. Why this is different from a variety of other studies that do not report any inhibition of osteogenic differentiation at normoxia is still not well explained. It may be due to the fact that the Authors here look at a subset of BMSCs as not all of them are Sca1 and 140a positive according to many previous reports e.g. from D Scadden group. This should be discussed in more detail as a limitation of this study. Moreover, when the Authors show Nile Red staining in these cells to prove changes in lipid metabolism, these cells look like adipocytes even without adipogenic induction. The explanation here can be that 2% hypoxia pushes them towards adipogenic lineage in uninduced conditions. What is the level of adipogenic markers in these cells at baseline in regular media?

<u>Response</u>: We have previously addressed this concern in response to a similar comment made by Reviewer #3 (comment 3). For the reviewer's convenience we copy our response below:

'The profound loss of the osteogenic differentiation capacity upon exposure of cells to normoxic conditions was highly reproducible and observed each time the experiment was repeated (n=3 biological replicates for hypoxic samples; n=5 biological replicates for normoxic and reversed-hypoxic samples). The dramatic loss of the osteogenic differentiation capacity that we observed in normoxic cells, is in line with previously

published reports (e.g., PMID: 23029528, PMID: 31827540, PMID: 25109357) demonstrating strong defects in osteogenesis of normoxia-cultured MSCs. However, we agree with the reviewer that some studies have shown osteogenic differentiation capacity under normoxic conditions. We speculate that the profound loss of osteogenic potential that we report here might be explained by:

i) the unique properties of the specific stem cell population used in each study. Here we used a very stringent, FACS-based approach to isolate pure MSCs, whereas other studies are based on the 'plastic-adherence' method that results in a 'contaminated' MSC population.

ii) the exact oxygen conditions under which the cells were cultured. Although we cultured MSCs under 2% O_2, which resembles the oxygen concentration found in their niche, in several reports the authors culture MSCs under extreme hypoxic conditions, i.e., less than 1% O_2, which might affect differently the stem cell behavior,

iii) the duration of the exposure to different oxygen conditions. Long- or short-term exposure to high oxygen tension results in different phenotypes and affects differently the stem cell function, as has been previously reported (e.g., PMID: 25034305),

iv) the direction of the shift in the oxygen tension. Although MSCs in several studies are cultured under high oxygen levels and are then moved to hypoxic conditions, here we followed the opposite approach. Thus, we initially expanded these cells under hypoxia, to mimic the oxygen tension found in their physiological niche, and we then shifted them to normoxia, and

v) the exact timepoint at which the osteogenic differentiation was assessed. In most studies the osteogenic differentiation is evaluated 21 days after induction of differentiation. However, we focused on an earlier timepoint and assessed differences in osteogenesis 11 days after inducing differentiation.

All these parameters could dramatically alter the MSC activity, contributing to the profound decrease in the osteogenic differentiation capacity that we observed in normoxic MSCs'.

We would also like to emphasize that, opposite to Reviewer #1, we strongly believe that the stringent isolation criteria we followed here and the unique properties of the purified MSC population is not a limitation, but rather a major advantage, of our study. In fact, other stem cell studies are often based on a mixed population of mesenchymal cells. We are convinced

that a population as pure as possible is critical in understanding epigenetic contribution to differentiation potential as epigenetic signals represent an average of various cell states in mixed populations.

Additionally, as shown in **R-Fig.1**, isolated MSCs cultured under hypoxic or normoxic conditions in control (regular, unstimulated) medium, exhibit similar expression levels of the *Fabp4* adipogenic gene, suggesting that the difference in the lipid content **(Fig. EV3)** is not due to commitment of hypoxic cells towards adipocytes under basal culture conditions. In line with this, in our recent study, we confirmed with and RNA-seq and FACS-based analyses that MSCs isolated both from young and old mice maintained their mesenchymal identity throughout the culture period in hypoxic conditions, arguing against cell commitment to adipocytes (Pouikli *et al*., 2021).

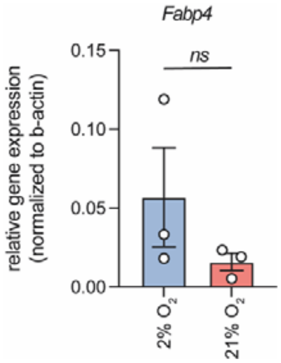

R-Fig.1. qPCR analysis of the expression levels of the Fabp4 adipogenic gene. β-actin expression levels were used as an internal control for normalization (n=3 biological replicates).

Another concern was raised before, that there was no direct evidence of changes of AcCoA pool in mito and cytosol. It was suggested to use digitonin permeabilization technique to obtain mito and cytosolic fractions. For cell fractionation with digitonin, the Authors followed a previously published protocol. However, why that particular protocol was chosen is not clear. First, they used PBS in their permeabilization media which is not appropriate media for mitochondria. Pls see a review by Brand and Nicholls (doi:10.1042/BJ20110162) for appropriate media. It should be potassium-based and have various other components necessary for mitochondria. Moreover, 1 mg/ml digitonin, which is 0.1% solution is quite high and can permeabilize mitochondrial membrane. Usually 0.01% is sufficient to permeabilize plasma membrane. Mitotracker retention used in the paper by Lee et al is not an appropriate method to assess mitochondrial integrity after treatment with digitonin. Once Mitotracker is

accumulated in mito, it forms disulfide bonds to proteins and stays there even if mito integrity is compromised (see above review for details). Blotting for tubulin and TOMM20 used by the Authors, does not guarantee 'purity' of fractions since tubulin is a structural protein, not capable of moving freely into the mito while Tomm20 is a mito membrane protein, that is not expected to be released into cytosol even if the mito membrane is permeabilized. Moreover, permeabilization may not be sufficient to release proteins but just small metabolites. Therefore, fig 4 experiments and other data dependent on this technique remain questionable.

Response: The main reason for choosing the above-mentioned protocol for our cell fractionation experiment that was coupled to metabolomic analysis is that it is the fastest protocol available for cell fractionation. Given that we were interested in measuring levels of citrate and acetyl-CoA, both of which are labile molecules, it was crucial for us to follow an approach that ensures rapid, yet efficient, isolation of the mitochondrial and cytosolic fractions, maintaining intact the pool of the metabolites in each compartment. The digitonin-based approach that Lee and colleagues developed, allows for such a quick subcellular fractionation. In fact, in the original publication Lee *et al*. report that "delaying the metabolite extraction from the mitochondrial fraction by a few minutes resulted in diffusion of metabolites from the mitochondrial matrix", highlighting the necessity for a rapid fractionation protocol to preserve the metabolite pool in the two sub-cellular compartments (PMID: 30903027, Fig. 1f). Thus, this protocol is the most suitable for the detection of citrate and acetyl-CoA, which could be lost/diffused during the long centrifugation steps of sucrose-based protocols. Furthermore, the authors also used various genetic, which caused the expected changes in the levels of the isolated metabolites measured in the different sub-cellular compartments, thus validating that their protocol accurately captures changes in the metabolite pools. Despite the fact that this manuscript was just published in 2019, it has already been cited 40 times (https://nature.altmetric.com/details/57727302/citations), indicating that the method is well accepted in the field.

To show the mitochondrial integrity more directly, we have now included an additional control and we present western blot data for the cytochrome c oxidase (MT-CO1). As shown in **Fig. EV4C**, the MT-CO1 protein was detected in the whole-cell and mitochondrial fractions, whereas it was undetectable in the cytosolic fractions, confirming that digitonin

treatment did not impact the integrity of the isolated mitochondria. Additionally, we would also like to clarify here that the isolated mitochondria were only used for metabolite extraction immediately after subcellular fractionation; thus, functionality of the isolated mitochondria is not relevant for this study.

We have now explained more clearly in the manuscript the reasons for choosing this method (lines 246-250) and we have included the MT-CO1 blot in the **Fig. EV4**.

Again, ac-lysine immunofluorescence was used as a marker of increased acetylation and AcCoA trapping in mito as shown in Fig 5. In fact, the magnified regions in fig 5D show nuclear and non-nuclear distribution of ac-lysine depending on the condition. How mitochondrial signal is defined is not clear as there is very little co-localization of TOMM20 and ac-lysine signals. Therefore, the conclusion that there is increased ac-lysine in mito and trapping of AcCoA in mito is questionable. In sum, the data on chromatin accessibility in 2 vs 21% O2 in BMSCs is very interesting, however the mechanistic explanation for the observed differences is questionable. Moreover, it may also be applicable only to a subset of BMSCs (Sca1+ cd140+).

Response: In **Fig. 5D** the mitochondrial signal of the acetyl-Lysine antibody is defined based on its co-localization with the mitochondrial protein TOMM20. As explained in the figure legend, we manually assigned the localization of the acetyl-Lysine signal into three categories: exclusively nuclear (when it was found only in the nucleus), exclusively mitochondrial (when it co-localized with TOMM20 and was absent from the nucleus) and both mitochondrial/nuclear (when was present in the nucleus and co-localized with TOMM20). In **Fig. 5E** we plotted the percentage of cells in each condition that exhibited exclusively nuclear, exclusively mitochondrial or mito/nuclear localization of the acetyl-Lysine signal, following the quantitative criteria described above. As shown in **Fig. 5E**, the percentage of cells exhibiting co-localization between TOMM20 and acetyl-Lysine signal (thus, increased mitochondrial acetyl-CoA localization) is higher in normoxic and BTA-treated hypoxic cells. By contrast, the mean fluorescent intensity of the nuclear acetyl-Lysine signal (indicative of histone acetylation) is higher in hypoxic and acetate-treated normoxic cells **(Fig. 5F).**

Due to differences in the signal distribution and the intensities of the two antibodies (i.e., the TOMM20 antibody has a clear, mitochondrial localization and high signal intensity; by contrast, the acetyl-Lysine antibody exhibits strong nuclear signal but weaker extra-nuclear/mitochondrial signal) the co-localization between TOMM20 and acetyl-Lysine is not always visible in the un-magnified images. Therefore, as described in the figure legend, we provide magnified insets, where we adjusted similarly in all images the intensity of the acetyl-Lysine signal, for visualization purposes.

Referee#3

Pouikli et al. reported hypoxic conditions promoted osteogenesis via accumulation of acetyl-CoA and increasing histone acetylation, which is intriguing and vital in the field. The revised vision answered all the questions raised previously. We recommend this work should be accepted.

Response: We thank the reviewer for the positive evaluation of our work.

References

1. Lee WD, Mukha D, Aizenshtein E & Shlomi T (2019) Spatial-fluxomics provides a subcellular-compartmentalized view of reductive glutamine metabolism in cancer cells. *Nat Commun* 10: 1351.

2. Pouikli, A., Parekh, S., Maleszewska, M., Nikopoulou, C., Baghdadi, M., Tripodi, I., FolzDonahue, K., Hinze, Y., Mesaros, A., Hoey, D., et al. (2021). Chromatin remodeling due to degradation of citrate carrier impairs osteogenesis of aged mesenchymal stem cells. Nature Aging *1*, 810–825.

2nd Revision - Editorial Decision 23rd Sep 2022

Dear Peter,

Congratulations on an excellent manuscript, I am pleased to inform you that your manuscript has been accepted for publication in The EMBO Journal. Thank you for your comprehensive response to the referee concerns. It has been a pleasure to work with you to get this to the acceptance stage.

I will begin the final checks on your manuscript before submitting to the publisher next week. Once at the publisher, it will take about 3 weeks for your manuscript to be published online. As a reminder, the entire review process, including referee concerns and your point-by-point response, will be available to readers.

I will be in touch throughout the final editorial process until publication. In the meantime, I hope you find time to celebrate!

If you have any questions, please do not hesitate to call or email the Editorial Office. Thank you for your contribution to The EMBO Journal.

Yours sincerely,

Kelly

Kelly M Anderson, PhD
Editor
The EMBO Journal
k.anderson@embojournal.org

Please note that it is EMBO Journal policy for the transcript of the editorial process (containing referee reports and your response letter) to be published as an online supplement to each paper. If you do NOT want this, you will need to inform the Editorial Office via email immediately. More information is available here:
https://www.embopress.org/page/journal/14602075/authorguide#transparentprocess

Your manuscript will be processed for publication in the journal by EMBO Press. Manuscripts in the PDF and electronic editions of The EMBO Journal will be copy edited, and you will be provided with page proofs prior to publication. Please note that supplementary information is not included in the proofs.

You will be contacted by Wiley Author Services to complete licensing and payment information. The required 'Page Charges Authorization Form' is available here: https://www.embopress.org/pb-assets/embo-site/tej_apc.pdf - please download and complete the form and return to embopressproduction@wiley.com

EMBO Press participates in many Publish and Read agreements that allow authors to publish Open Access with reduced/no publication charges. Check your eligibility: https://authorservices.wiley.com/author-resources/Journal-Authors/open-access/affiliation-policies-payments/index.html

Should you be planning a Press Release on your article, please get in contact with embojournal@wiley.com as early as possible, in order to coordinate publication and release dates.

EMBO Press Author Checklist

Corresponding Author Name: Peter Tessarz
Journal Submitted to: The EMBO Journal
Manuscript Number: EMBOJ-2022-111239

USEFUL LINKS FOR COMPLETING THIS FORM
The EMBO Journal - Author Guidelines
EMBO Reports - Author Guidelines
Molecular Systems Biology - Author Guidelines
EMBO Molecular Medicine - Author Guidelines

Reporting Checklist for Life Science Articles (updated January 2022)
This checklist is adapted from Materials Design Analysis Reporting (MDAR) Checklist for Authors. MDAR establishes a minimum set of requirements in transparent reporting in the life sciences (see Statement of Task: 10.31222/osf.io/9sm4x). Please follow the journal's guidelines in preparing your manuscript.
Please note that a copy of this checklist will be published alongside your article.

Abridged guidelines for figures
1. Data
The data shown in figures should satisfy the following conditions:
- the data were obtained and processed according to the field's best practice and are presented to reflect the results of the experiments in an accurate and unbiased manner.
- ideally, figure panels should include only measurements that are directly comparable to each other and obtained with the same assay.
- plots include clearly labeled error bars for independent experiments and sample sizes. Unless justified, error bars should not be shown for technical replicates.
- if n<5, the individual data points from each experiment should be plotted. Any statistical test employed should be justified.
- Source Data should be included to report the data underlying figures according to the guidelines set out in the authorship guidelines on Data Presentation.

2. Captions
Each figure caption should contain the following information, for each panel where they are relevant:
- a specification of the experimental system investigated (eg cell line, species name).
- the assay(s) and method(s) used to carry out the reported observations and measurements.
- an explicit mention of the biological and chemical entity(ies) that are being measured.
- an explicit mention of the biological and chemical entity(ies) that are altered/varied/perturbed in a controlled manner.
- the exact sample size (n) for each experimental group/condition, given as a number, not a range;
- a description of the sample collection allowing the reader to understand whether the samples represent technical or biological replicates (including how many animals, litters, cultures, etc.).
- a statement of how many times the experiment shown was independently replicated in the laboratory.
- definitions of statistical methods and measures:
  - common tests, such as t-test (please specify whether paired vs. unpaired), simple χ2 tests, Wilcoxon and Mann-Whitney tests, can be unambiguously identified by name only, but more complex techniques should be described in the methods section;
  - are tests one-sided or two-sided?
  - are there adjustments for multiple comparisons?
  - exact statistical test results, e.g., P values = x but not P values < x;
  - definition of 'center values' as median or average;
  - definition of error bars as s.d. or s.e.m.

Please complete ALL of the questions below.
Select "Not Applicable" only when the requested information is not relevant for your study.

Materials

Newly Created Materials	Information included in the manuscript?	In which section is the information available? (Reagents and Tools Table, Materials and Methods, Figures, Data Availability Section)
New materials and reagents need to be available; do any restrictions apply?	Yes	Materials and Methods, Data Availability Section, Supplementary Information

Antibodies	Information included in the manuscript?	In which section is the information available? (Reagents and Tools Table, Materials and Methods, Figures, Data Availability Section)
For **antibodies** provide the following information:<br>- Commercial antibodies: RRID (if possible) or supplier name, catalogue number and or/clone number<br>- Non-commercial: RRID or citation	Yes	Materials and Methods

DNA and RNA sequences	Information included in the manuscript?	In which section is the information available? (Reagents and Tools Table, Materials and Methods, Figures, Data Availability Section)
**Short novel DNA or RNA including primers, probes**: provide the sequences.	Not Applicable	

Cell materials	Information included in the manuscript?	In which section is the information available? (Reagents and Tools Table, Materials and Methods, Figures, Data Availability Section)
**Cell lines:** Provide species information, strain. Provide accession number in repository **OR** supplier name, catalog number, clone number, and/**OR** RRID.	Yes	Materials and Methods
**Primary cultures:** Provide species, strain, sex of origin, genetic modification status.	Yes	Materials and Methods
Report if the cell lines were recently **authenticated** (e.g., by STR profiling) and tested for mycoplasma contamination.	Yes	Materials and Methods

Experimental animals	Information included in the manuscript?	In which section is the information available? (Reagents and Tools Table, Materials and Methods, Figures, Data Availability Section)
**Laboratory animals or Model organisms:** Provide species, strain, sex, age, genetic modification status. Provide accession number in repository **OR** supplier name, catalog number, clone number, **OR** RRID.	Yes	Materials and Methods
**Animal observed in or captured from the field:** Provide species, sex, and age where possible.	Not Applicable	
Please detail **housing and husbandry conditions**.	Yes	Materials and Methods

Plants and microbes	Information included in the manuscript?	In which section is the information available? (Reagents and Tools Table, Materials and Methods, Figures, Data Availability Section)
**Plants:** provide species and strain, ecotype and cultivar where relevant, unique accession number if available, and source (including location for collected wild specimens).	Not Applicable	
**Microbes:** provide species and strain, unique accession number if available, and source.	Not Applicable	

Human research participants	Information included in the manuscript?	In which section is the information available? (Reagents and Tools Table, Materials and Methods, Figures, Data Availability Section)
If collected and within the bounds of privacy constraints report on age, sex and gender or ethnicity for all study participants.	Not Applicable	

Core facilities	Information included in the manuscript?	In which section is the information available? (Reagents and Tools Table, Materials and Methods, Figures, Data Availability Section)
If your work benefited from core facilities, was their service mentioned in the acknowledgments section?	Yes	Acknowledgements

Design

Study protocol	Information included in the manuscript?	In which section is the information available? (Reagents and Tools Table, Materials and Methods, Figures, Data Availability Section)
If study protocol has been **pre-registered, provide DOI in the manuscript**. For clinical trials, provide the trial registration number **OR** cite DOI.	Not Applicable	
Report the **clinical trial registration number** (at ClinicalTrials.gov or equivalent), where applicable.	Not Applicable	

Laboratory protocol	Information included in the manuscript?	In which section is the information available? (Reagents and Tools Table, Materials and Methods, Figures, Data Availability Section)
Provide DOI OR other citation details if **external detailed step-by-step protocols** are available.	Not Applicable	

Experimental study design and statistics	Information included in the manuscript?	In which section is the information available? (Reagents and Tools Table, Materials and Methods, Figures, Data Availability Section)
Include a statement about **sample size** estimate even if no statistical methods were used.	Yes	Materials and Methods and respective Figure legends
Were any steps taken to minimize the effects of subjective bias when allocating animals/samples to treatment (e.g. **randomization procedure**)? If yes, have they been described?	Not Applicable	
Include a statement about **blinding** even if no blinding was done.	Not Applicable	
Describe **inclusion/exclusion criteria** if samples or animals were excluded from the analysis. Were the criteria pre-established?<br><br>If sample or data points were omitted from analysis, report if this was due to attrition or intentional exclusion and provide justification.	Not Applicable	
For every figure, are **statistical tests** justified as appropriate? Do the data meet the assumptions of the tests (e.g., normal distribution)? Describe any methods used to assess it. Is there an estimate of variation within each group of data? Is the variance similar between the groups that are being statistically compared?	Yes	Figure legends

Sample definition and in-laboratory replication	Information included in the manuscript?	In which section is the information available? (Reagents and Tools Table, Materials and Methods, Figures, Data Availability Section)
In the figure legends: state number of times the experiment was **replicated** in laboratory.	Yes	Figure legends
In the figure legends: define whether data describe **technical or biological replicates**.	Yes	Figure legends

Ethics

Ethics	Information included in the manuscript?	In which section is the information available? (Reagents and Tools Table, Materials and Methods, Figures, Data Availability Section)
Studies involving **human participants**: State details of **authority granting ethics approval** (IRB or equivalent committee(s), provide reference number for approval.	Not Applicable	
Studies involving **human participants**: Include a statement confirming that **informed consent** was obtained from all subjects and that the experiments conformed to the principles set out in the WMA Declaration of Helsinki and the Department of Health and Human Services Belmont Report.	Not Applicable	
Studies involving **human participants:** For publication of **patient photos**, include a statement confirming that consent to publish was obtained.	Not Applicable	
Studies involving experimental **animals**: State details of **authority granting ethics approval** (IRB or equivalent committee(s), provide reference number for approval. Include a statement of compliance with ethical regulations.	Yes	Materials and Methods
Studies involving **specimen and field samples:** State if relevant **permits** obtained, provide details of authority approving study; if none were required, explain why.	Not Applicable	

Dual Use Research of Concern (DURC)	Information included in the manuscript?	In which section is the information available? (Reagents and Tools Table, Materials and Methods, Figures, Data Availability Section)
Could your study fall under dual use research restrictions? Please check biosecurity documents and list of **select agents and toxins** (CDC): https://www.selectagents.gov/sat/list.htm	Not Applicable	
If you used a select agent, is the security level of the lab appropriate and reported in the manuscript?	Not Applicable	
If a study is subject to dual use research of concern regulations, is the name of the **authority granting approval and reference number** for the regulatory approval provided in the manuscript?	Not Applicable	

Reporting

The MDAR framework recommends adoption of discipline-specific guidelines, established and endorsed through community initiatives. Journals have their own policy about requiring specific guidelines and recommendations to complement MDAR.

Adherence to community standards	Information included in the manuscript?	In which section is the information available? (Reagents and Tools Table, Materials and Methods, Figures, Data Availability Section)
State if relevant guidelines or checklists (e.g., **ICMJE, MIBBI, ARRIVE, PRISMA**) have been followed or provided.	Not Applicable	
For **tumor marker prognostic studies,** we recommend that you follow the **REMARK** reporting guidelines (see link list at top right). See author guidelines, under 'Reporting Guidelines'. Please confirm you have followed these guidelines.	Not Applicable	
For **phase II and III randomized controlled trials**, please refer to the **CONSORT** flow diagram (see link list at top right) and submit the CONSORT checklist (see link list at top right) with your submission. See author guidelines, under 'Reporting Guidelines'. Please confirm you have submitted this list.	Not Applicable	

Data Availability

Data availability	Information included in the manuscript?	In which section is the information available? (Reagents and Tools Table, Materials and Methods, Figures, Data Availability Section)
Have **primary datasets** been deposited according to the journal's guidelines (see 'Data Deposition' section) and the respective accession numbers provided in the Data Availability Section?	Yes	Data Availability Section
Were **human clinical and genomic datasets** deposited in a public access-controlled repository in accordance to ethical obligations to the patients and to the applicable consent agreement?	Not Applicable	
Are **computational models** that are central and integral to a study available without restrictions in a machine-readable form? Were the relevant accession numbers or links provided?	Not Applicable	
If publicly available data were reused, provide the respective **data citations in the reference list**.	Not Applicable	
